# A generalized regression model of arsenic variations in the shallow groundwater of Bangladesh

**DOI:** 10.1002/2013WR014572

**Published:** 2015-01-28

**Authors:** Mohammad Shamsudduha, Richard G. Taylor, Richard E. Chandler

**Affiliations:** ^1^ Institute for Risk and Disaster Reduction, University College LondonLondonUK; ^2^ Department of GeographyUniversity College LondonLondonUK; ^3^ Department of Statistical ScienceUniversity College LondonLondonUK

**Keywords:** arsenic, groundwater‐fed irrigation, recharge, regression model, Bangladesh

## Abstract

Localized studies of arsenic (As) in Bangladesh have reached disparate conclusions regarding the impact of irrigation‐induced recharge on As concentrations in shallow (≤50 m below ground level) groundwater. We construct generalized regression models (GRMs) to describe observed spatial variations in As concentrations in shallow groundwater both (i) nationally, and (ii) regionally within Holocene deposits where As concentrations in groundwater are generally high (>10 μg L^−1^). At these scales, the GRMs reveal statistically significant inverse associations between observed As concentrations and two covariates: (1) hydraulic conductivity of the shallow aquifer and (2) net increase in mean recharge between predeveloped and developed groundwater‐fed irrigation periods. Further, the GRMs show that the spatial variation of groundwater As concentrations is well explained by not only surface geology but also statistical interactions (i.e., combined effects) between surface geology and mean groundwater recharge, thickness of surficial silt and clay, and well depth. Net increases in recharge result from intensive groundwater abstraction for irrigation, which induces additional recharge where it is enabled by a permeable surface geology. Collectively, these statistical associations indicate that irrigation‐induced recharge serves to flush mobile As from shallow groundwater.

## Key Points

- GRMs explain the spatial variation in As concentrations in Bangladesh
- Inverse associations between As, recharge, hydraulic conductivity, and irrigation
- GRMs indicate that recharge serves to flush mobile As from groundwater

## 1 Introduction

Biogeochemical controls on aqueous arsenic (As) concentrations in very shallow (≤50 m below ground level) groundwater in the Bengal Basin have been studied extensively over the last two decades [*Nickson et al*., 1998; *McArthur et al*., 2004; *Zheng et al*., 2005; *Harvey et al*., 2006; *Mukherjee et al*., 2008; *Chowdhury et al*., 2012]. This research has primarily been based upon localized observations in Bangladesh and West Bengal where As concentrations in shallow groundwater are generally high (exceeding the WHO standard of 10 μg L^−1^). The general consensus from this work is that As derives from the microbially mediated, reductive dissolution of iron‐oxyhydroxide minerals in alluvial sediments [*Bhattacharya et al*., 1997; *Nickson et al*., 2000; *McArthur et al*., 2004; *Ravenscroft et al*., 2009]. In addition to natural controls (e.g., sediment lithology, mineralogy, geochemistry) on the spatial distribution of As concentrations in shallow groundwater, several authors [*Harvey et al*., 2006; *Klump et al*., 2006; *Neumann et al*., 2010] assert that intensive pumping of groundwater for irrigation has changed the spatial distribution of As concentrations by perturbing groundwater flow systems in the Bengal Basin. They show that irrigation abstraction of groundwater for dry‐season Boro rice cultivation can mobilize As by inducing recharge laden with reactive organic carbon (OC) derived primarily from surface sources (e.g., ponds) in Bangladesh. Using observational data and groundwater flow modeling in a localized study area, *Neumann et al*. [2010, 2011] show that groundwater recharge from anthropogenic ponds, rich in biologically available OC, produces groundwater elevated in arsenic, whereas recharge derived from rice‐field irrigation return flows gives rise to groundwater low in arsenic. The latter mechanism is consistent with the assertion that recharge induced by groundwater pumping serves primarily to flush mobile As from alluvial aquifers in the Bengal Basin [*van Geen et al*., 2003; *Stute et al*., 2007; *Datta et al*., 2011; *McArthur et al*., 2011a; *Reich*, 2011].

**Figure 1:**
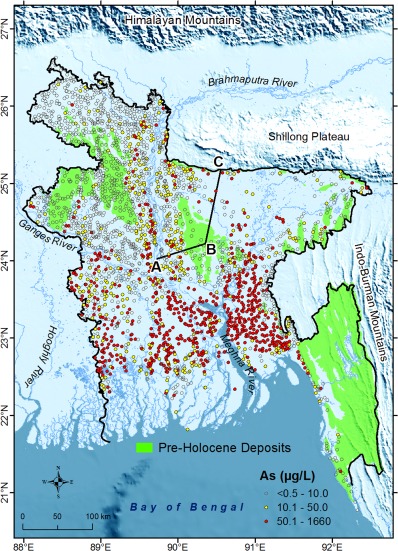
As concentrations in very shallow (≤50 m bgl) groundwater in Bangladesh sampled under the National Hydrochemical Survey [*DPHE*, 1999; *BGS and DPHE*, 2001]. The Pre‐Holocene deposits (e.g., Madhupur Clay) are shown in green; the rest of Bangladesh is covered with Holocene alluvium. The background image is a digital elevation model showing the hilly terrains surrounding the Bengal Basin. Arsenic concentrations in a hydrogeological cross section along the transect (A‐B‐C) are shown in Figure 2.

**Figure 2:**
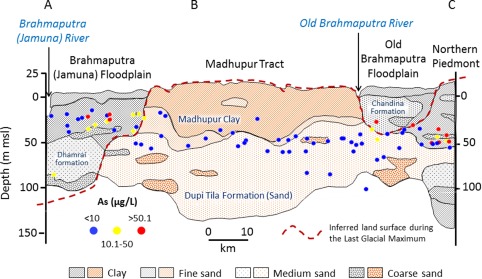
Hydrogeological cross section from north‐central part of Bangladesh [*Ravenscroft*, 2003] shows the Plio‐Pleistocene and Holocene aquifers in the Bengal Basin. Shallow groundwater As concentrations observed in the National Hydrochemical Survey [*DPHE*, 1999; *BGS and DPHE*, 2001] are plotted along the hydrogeological transect shown in Figure 1 (samples within a 10 km window from either side of the transect are plotted).

With few exceptions [*BGS and DPHE*, 2001; *Ahmed et al*., 2004; *Ravenscroft et al*., 2005], published research on the variation in As concentrations has been based on local‐scale studies. These studies provide a valuable aid to understanding but neglect the additional information that can be derived by examining regional‐scale variations in As concentrations (see Figures 1 and 2). Such regional‐scale variations have previously been attributed to surface geology and underlying sediment characteristics [*BGS and DPHE*, 2001; *Ravenscroft*, 2001; *Ahmed et al*., 2004; *McArthur et al*., 2008]. However, human activity over the last four decades has substantially affected the shallow groundwater system in Bangladesh [*Michael and Voss*, 2009; *Shamsudduha et al*., 2009a]: shallow groundwater abstraction has induced greater recharge in areas underlain by a permeable surface geology but reduced groundwater storage elsewhere [*Shamsudduha et al*., 2011]. By examining the regional variation in As concentrations and its relationship with abstraction (taking account of geological and hydrological controls), it becomes possible to extract large‐scale signals that complement understanding derived from local‐scale studies. Indeed, from the perspective of a policymaker, it is arguably the large‐scale and regional structure that is more relevant since local‐scale policy interventions are difficult to implement. Moreover, given the complexity and variability of the As mobilization process, it is possible that local‐scale studies will not yield sufficient quantities of data to identify genuine signals reliably: by performing a single integrated analysis of all available observations, the amount of data is correspondingly increased so that signals are more easily identified by “borrowing strength” from similar sites [e.g., *Katz et al*., 2003].

These considerations motivate us to examine statistical relationships between As concentrations in shallow groundwater and a set of relevant large‐scale hydrogeological and hydrodynamic factors that have been associated with As concentrations [*BGS and DPHE*, 2001; *Ravenscroft*, 2001; *Ahmed et al*., 2004; *Ravenscroft et al*., 2005; *Harvey et al*., 2006; *van Geen et al*., 2008; *Shamsudduha et al*., 2009b]. We construct generalized regression models to explain the observed spatial variation in As concentrations in shallow groundwater throughout the Bengal Basin of Bangladesh. This statistical analysis requires care because the As data set [*DPHE*, 1999; *BGS and DPHE*, 2001] features: (1) a highly skewed (nonnormal) distribution, (2) many records below detection limits, and (3) dependence between observations from neighboring spatial locations. We use the constructed models to quantify the overall effects of critical hydrogeological and hydrodynamic variables on As concentrations.

Section 2 describes the groundwater As and covariate data sets as well as the modeling strategy. Model outputs and interpretation of results are presented in section 3. Modeled associations between groundwater As and key covariates and their relevance to groundwater use for irrigation are discussed in section 4. The major outcomes of this study are summarized in section 5.

## 2 Methods and Data Sets

The aim of this work is to investigate the simultaneous effect of multiple factors upon shallow groundwater As concentrations. It is conventional in statistics to refer to the primary variable of interest (groundwater As concentration) as the “response variable” and to the potential influencing factors as “covariates.” In this section, we describe the response variable and covariate data sets used in this study, outlining the issues that must be addressed in order to produce a convincing outcome. This is followed by an explanation of the statistical methodology.

We construct generalized regression models (GRMs) at two spatial scales to explain the observed variation in As concentrations in shallow groundwater. The first model, hereafter known as the “national‐scale GRM,” explains the spatial variation in As concentrations nationally at 1643 locations that were surveyed [*DPHE*, 1999; *BGS and DPHE*, 2001] once during 1998 and 1999. Time series data of As concentrations cannot be used here as they do not exist at the national scale [*Fendorf et al*., 2010]. A second model, hereafter the “regional‐scale GRM,” addresses a potential bias in the national‐scale GRM due to the inclusion of large areas of north‐central (Madhupur Tract) and northwestern (Barind Tract) Bangladesh (Figures 1 and 2) where As concentrations are generally low (<10 μg L^−1^). The comparative absence of As in these Plio‐Pleistocene aquifers of the Bengal Basin has been attributed to: (1) the flushing of mobile As under increased groundwater flow induced by greater vertical hydraulic gradients that occurred naturally during the Last Glacial Maximum (LGM) (20 *ka*) when sea levels were nearly 120 m lower than present [*BGS and DPHE*, 2001; *McArthur et al*., 2008], and (2) the greater As‐adsorption properties of brown (oxidized) sediments that comprise these Plio‐Pleistocene aquifers [*Radloff et al*., 2011].

### 2.1 Groundwater Arsenic Data Set

We use a total of 2410 single observations of As concentrations in groundwater from wells with an intake depth of ≤50 m bgl (Figure 1 and supporting information Figure S1). The observations were sampled under the National Hydrochemical Survey (NHS) in Bangladesh conducted jointly by the British Geological Survey (BGS), the Department of Public Health Engineering, Bangladesh (DPHE), and Mott MacDonald (UK) during 1998 and 1999 [*DPHE*, 1999; *BGS and DPHE*, 2001]. The coordinates for sampling locations were taken by the hand‐held Global Positioning System [*BGS and DPHE*, 2001]. Groundwater samples were analyzed for As concentrations by two different techniques: hydride generation‐atomic fluorescence spectrometry (HG‐AFS) and hydride generation‐ICP AES (Inductively Coupled Plasma Atomic Emission Spectroscopy). The detection limits of As concentrations for the HG‐AFS and HG‐ICP‐AES methods were 0.5 and 6.0 μg L^−1^ respectively.

The groundwater As data set features a number of important characteristics described below. The distribution of observed As concentrations in Bangladesh is highly (positively) skewed, with values ranging from <0.5 to 1660 μg L^−1^. The spatial distribution of groundwater As concentrations is also highly variable throughout the country [*Gaus et al*., 2003; *Yu et al*., 2003; *Shamsudduha*, 2007]. However, higher As concentrations (>50 μg L^−1^) are observed in most parts of southern Bangladesh [*DPHE*, 1999; *BGS and DPHE*, 2001]. Of the 2410 As measurements, 743 (31%) are reported as below analytical detection limits. In the statistical literature, such values are described as being “censored.” The presence of censored values requires care in any statistical analysis. In environmental applications, nondetects in skewed data are most commonly handled by replacing each value with one‐half of the detection limit and then using a logarithmic transformation to normalize the distribution [*Helsel*, 2005]. However, this approach can lead to substantial bias in estimates of descriptive statistics (i.e., mean, variance), and can also severely distort regression coefficients and their standard errors [*Helsel*, 2006; *Antweiler and Taylor*, 2008; *Helsel*, 2010]. Consequently, we calculate the basic statistics of As data with censored observations using the Regression on Order Statistics (ROS) and Kaplan‐Meier (K‐M) methods [*Lee and Helsel*, 2007]. Moreover, when building models to describe the effect of multiple covariates upon As concentrations, we use methods that account explicitly for the censoring (see section 2.3 below).

A further feature to consider is the potential presence of variations in As concentrations that arise, at least in part, from local‐scale hydrodynamical, geological, and geochemical controls that cannot be incorporated explicitly into the analysis because the required data are not available at a national scale. In statistical terms, the effect of these local‐scale controls is to induce dependence between observations from neighboring sites that cannot be explained using the large‐scale covariates that are the focus of interest in this study. This dependence invalidates the usual standard errors and confidence intervals for model parameters, which must therefore be adjusted to ensure a correct analysis [*Chandler*, 2005; *Helsel*, 2005]. By carrying out such adjustments, the effects of local‐scale influences on As concentration are accounted for implicitly.

### 2.2 Covariate Data Sets: Rationale and Description

Sixteen covariates were considered in the development of our statistical models (Table S1). We group these covariates into four broad categories: (1) six geological and hydrogeological variables (Surface geology, Thickness of surficial silt and clay, Hydraulic conductivity, Specific yield, Darcy flux, Well depth and its statistical interaction with surface geology), (2) five hydrodynamic and groundwater recharge variables (Wet‐season groundwater table, Groundwater‐level trends, Mean groundwater fluctuation, Mean PGI recharge and its statistical interaction with surface geology, Net changes in recharge), (3) four geographical and seasonal variables (Latitude and Longitude, Surface elevation, Seasonality (sine + cosine of sampling dates)), and (4) groundwater‐fed irrigation (Irrigation trends (1985−1999)). This section provides a rationale for the inclusion of these variables as well as details of their data sets and processing.

Previous studies [*DPHE*, 1999; *BGS and DPHE*, 2001; *Ravenscroft*, 2001; *Ravenscroft et al*., 2005] have examined statistical relationships between groundwater As and both geological and hydrogeological factors in isolation. A previous study [*Ravenscroft*, 2001] correlated As concentrations with mean groundwater levels and found that low As concentrations (<10 μg L^−1^) are associated with deepest groundwater levels. High As concentrations (>50 μg L^−1^) in tubewells are associated with shallow (<3 m bgl) water table in aquifers [*Shamsudduha et al*., 2009b]. Several localized studies (Figure S2) [*Ravenscroft*, 2001; *McArthur et al*., 2004; *Harvey et al*., 2006; *Klump et al*., 2006; *Stute et al*., 2007; *Polizzotto et al*., 2008; *Neumann et al*., 2010] relate the distribution of As in groundwater with recharge to aquifers and long‐term changes in recharge rates, yet it remains unclear as to whether rises in groundwater recharge are associated with decreased or increased As concentrations over time. Geological and geomorphological controls on the regional‐scale distribution of groundwater As have been suggested by several studies [*DPHE*, 1999; *BGS and DPHE*, 2001; *Ahmed et al*., 2004; *Ravenscroft et al*., 2005; *Stute et al*., 2007; *Aziz et al*., 2008; *van Geen et al*., 2008]. *Ravenscroft* [2001] demonstrated associations between groundwater As, surface geology (see Figures S3 and S4), and geomorphology in Bangladesh through descriptive statistics and linear regression analysis.

The influence of soil permeability and properties of near‐surface deposits on the spatial distribution of groundwater As concentrations have also been examined previously [*van Geen et al*., 2006; *Stute et al*., 2007; *Aziz et al*., 2008; *van Geen et al*., 2008]. These studies reveal that low As concentrations in groundwater are associated with areas of highly permeable soils and near‐surface geology. It is hypothesized that shallow aquifers beneath sandy soils receive rapid recharge from rainwater (and surface water bodies) that flushes As in groundwater by dilution. Recharge also supplies oxidants (dissolved oxygen and nitrate) that inhibit the reductive dissolution of iron oxy‐hydroxides and thus As mobilization in groundwater [*Aziz et al*., 2008]. In contrast, low‐permeability surface geology is thought to inhibit vertical recharge to the underlying aquifer where As is mobilized in groundwater under sustained reducing (iron oxyhydroxides) conditions. This hypothesis is supported by a study [*Stute et al*., 2007] that showed that As concentration in very shallow (<20 m bgl) aquifers is linearly correlated with groundwater residence time.

Each of the studies above considers the effect of a single factor or covariate in isolation. In reality of course, As concentrations are influenced by many factors acting in combination. Moreover, some of the factors are correlated (see Figure S5 for Pearson's correlation matrices) so that there is a danger of misinterpreting results if they are analyzed individually. The only way to ensure that the variation in As concentrations is correctly apportioned is via an analysis that considers the simultaneous effects of all relevant factors; hence the motivation for the present study, which attempts this for the first time.

#### 2.2.1 Seasonal Groundwater Levels and Temporal Trends

We employ a newly compiled national database of shallow groundwater levels [*Shamsudduha et al*., 2009a]. Statistics of weekly groundwater levels (i.e., mean depth to dry and wet‐season groundwater levels below ground level; hereafter GWT‐dry for dry‐season and GWT‐wet for wet‐season water table) were calculated for the period 1985–1999 at each monitoring site and interpolated nationally across Bangladesh. Wet‐season water levels determine the aquifer‐full condition following annual recharge, which predominantly takes place during the monsoon season [*Shamsudduha et al*., 2011]. The 1985–1999 time period is specifically chosen as it represents a period over which groundwater‐fed abstraction for dry‐season irrigation developed throughout Bangladesh and could have influenced As concentrations in the tubewells during the 1998/1999 sampling period [*DPHE*, 1999; *BGS and DPHE*, 2001]. The mean GWT‐dry was found not to add much information to the analysis due to a high degree of correlation (Pearson's correlation 0.75) with the mean GWT‐wet. Therefore, the mean GWT‐dry was not used in any of the subsequent modeling. In addition to the mean GWT‐wet, we use groundwater‐level trends (1985−1999) to examine the effect of long‐term changes in groundwater levels on spatial variations in As concentrations in shallow groundwater of Bangladesh. Values of mean GWT‐wet and groundwater‐level trends were extracted at each As sampling point using standard Geographical Information System (GIS) procedures described below.

Covariate data sets, which are numerical point observations but do not specifically coexist at the same location of As sampling, are interpolated and mapped nationally throughout Bangladesh. Values of each interpolated surface at each As location (*n* = 2410) are extracted using the spatial extraction function within ArcGIS (v. 9.3). For example, GIS maps for seasonal groundwater levels (GWT‐wet) were constructed using Ordinary Kriging with an appropriate model variogram. Small‐scale spatial variations in groundwater levels are expected to be smoothed due to spatial interpolation at the national scale, and there is inevitably some uncertainty in the interpolated values (see supporting information Table S3). For example, the root mean square error (RMSE) for the interpolated GWT‐wet is 1.34 m whereas the mean and standard deviation of GWT‐wet are 1.5 and 2.0 m. The RMSE of the interpolated mean groundwater‐level trends is 12 cm yr^−1^, whereas the mean and standard deviation are −3.74 and 16.7 cm yr^−1^ (for similar statistics for other covariates, see Table S3). Ideally, these interpolation uncertainties would be accounted for explicitly in our analysis. Unfortunately, however, to do so is formidably difficult. Although various methods are available in the statistical literature for tackling this problem in regression models (see *Carroll et al*. [2006], for a review), a considerable amount of methodological and computational development is required to implement these methods in the context of models that also account for censoring and for dependence between observations as we do here. We take a pragmatic approach; therefore, we do not explicitly account for the interpolation uncertainty in our modeling, but we note that relationships between interpolated covariates and As concentration will be weaker than those between true (unobserved) covariate values and As concentration. Thus, if we find statistically significant associations involving the interpolated covariates, we can be confident that there are genuine associations with the true covariates as well.

#### 2.2.2 Mean Groundwater Recharge and Net Changes in Recharge

**Figure 3:**
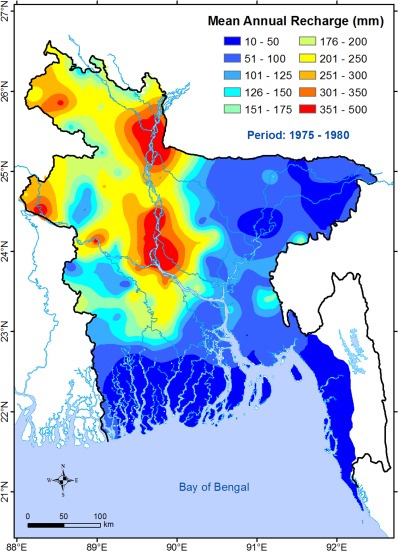
Map showing mean groundwater recharge for the predeveloped groundwater‐fed irrigation period (PGI; 1975−1980) in Bangladesh [*Shamsudduha et al*., 2011]. Estimates of mean recharge at 177 locations throughout Bangladesh have been interpolated applying a geostatistical interpolation method (Ordinary Kriging) with a modeled variogram.

We use: (i) mean annual (net) groundwater recharge over the period of 1975–1980 known as “predeveloped groundwater‐fed irrigation (PGI)” [*Shamsudduha et al*., 2011]; and (ii) net changes in recharge between the PGI period and developed groundwater‐fed irrigation (DGI; period 1985–1999) as covariates in the GRM. The required values have recently been estimated at the national scale in Bangladesh [*Shamsudduha et al*., 2011] using the water table fluctuation method with distributed specific yield values. PGI mean recharge (Figure 3) shows highest groundwater recharge along the River Brahmaputra and in some areas in northwestern Bangladesh. Variogram analysis reveals a strong spatial and directional dependence at the national scale (see Figure S6). Lowest groundwater recharge in the PGI period is observed in northeastern and southern Bangladesh.

We use the PGI mean groundwater recharge and net change in mean recharge between the PGI and DGI periods as two covariates representing the effect of groundwater recharge on the variation of As concentrations in the model. The PGI mean recharge is used to establish a “baseline” (i.e., groundwater recharge under predevelopment or nil‐pumping period) surface, and the “net change in recharge” then provides an opportunity to explain anomalies from this baseline surface in terms of the human intervention that has taken place in the last few decades [*Shamsudduha et al*., 2011]. The effect of the “net change in recharge” covariate thus provides insights into the real relationship between groundwater recharge and As concentrations at a regional scale.

#### 2.2.3 Surface Geology

Information on surface geology was extracted at each of the 2410 As sampling locations [*DPHE*, 1999; *BGS and DPHE*, 2001] from the geological map of Bangladesh (see supporting information Figure S3) [*Alam et al*., 1990]. The occurrence and spatial extent of shallow aquifers in Bangladesh follow the general distribution of the surface geology. A detailed description of surface geology and the aquifer systems in Bangladesh can be found in several studies [*UNDP*, 1982; *MPO*, 1987; *BGS and DPHE*, 2001]. Descriptive statistics of As concentrations within these geological units are summarized in Table S2. Mean groundwater As concentrations are highest (>100 μg L^−1^) in Chandina alluvium (“as,” see Figure S3 for details), deltaic sand (“dsd”), deltaic silt (“dsl”), and tidal deltaic deposits (“dt”); As concentrations vary with depth (Figures S3 and S7). Mean As concentrations are lowest (<10 μg L^−1^) in older alluvial fan (“afo”), bedrocks (“br”), Barind (“rb”), and Madhupur clay residuum (“rm”) in Bangladesh.

#### 2.2.4 Thickness of Surficial Silt and Clay (TSSC)

Shallow aquifers in Bangladesh are generally overlain by a silt and clay deposit, commonly known as the surficial silt and clay unit, throughout the country [*MPO*, 1987]. The thickness of surficial silt and clay (TSSC) ranges from <5 to 50 m [*Shamsudduha et al*., 2011]. In the alluvial fan deposits of northwestern Bangladesh, the TSSC is low (<5 m) where fine sands occur at the surface. In contrast, shallow aquifers occur at greater depths beneath the Madhupur and Barind Tracts, Sylhet depression, and the southern Ganges‐Brahmaputra‐Meghna Delta where the TSSC is higher (>15 m).

#### 2.2.5 Hydraulic Conductivity, Storage Coefficient, and Darcy Flux

At each sampling location, the hydraulic conductivity, storage coefficient (specific yield), and Darcy flow velocity of the shallow aquifer are considered as numerical covariates in the statistical model. The Bangladesh Water Development Board [*BWDB*, 1989, 1994] conducted pumping tests in shallow aquifers throughout Bangladesh and calculated horizontal hydraulic conductivity (*K_h_*) and specific yield (*S_y_*). Pumping‐test derived hydraulic properties of shallow aquifers have been compiled and GIS maps have been generated at the national scale [*Shamsudduha et al*., 2011]. Values of hydraulic conductivity and specific yield at the 2410 As sampling locations were extracted using geostatistical methods, in the same way as for groundwater covariates in section 2.2.1. In addition, groundwater flow velocity (Figure S8) (also known as the Darcy flux), which is a function of hydraulic conductivity and groundwater‐level gradient [*Hiscock*, 2005], has been calculated using hydraulic parameters compiled in this study. Darcy flux (*q*) has been calculated (*q* = −*K_h_i*) using the horizontal hydraulic conductivity [*Shamsudduha*, 2011] (*K_h_*) and the groundwater head gradient (*i*) [*Shamsudduha et al*., 2009a]. Values of Darcy flux at each of the As sampling locations have been estimated using the same interpolation procedure as for other hydraulic parameters (see supporting information Table S3 for interpolation errors). Similar comments apply here as previously, regarding the effect of interpolation errors on the conclusions from the analysis; there are, moreover, likely to be errors in the pumping test results which will have the same effect.

#### 2.2.6 Linear Trends in Groundwater‐Fed Irrigation

The Bangladesh Agricultural Development Corporation (BADC) has maintained a database of annual groundwater abstraction for irrigation since 2001. This database is recorded at the Thana/Upazila (third administrative unit in Bangladesh) level. However, no systematic record for irrigation existed before 2000 though groundwater‐fed irrigation in Bangladesh started during the early 1970s. Several studies [*UNDP*, 1982; *MPO*, 1987, 1991; *WARPO*, 2000] estimated groundwater abstraction for irrigation for some years (e.g., 1986, 1991, and 1996) at the national scale using information on number of irrigation pumps, discharge capacity, and pumping hours, or from agro‐climatic records [*BADC*, 2003; *Ravenscroft*, 2003]. This study has compiled all available data sets on groundwater abstraction for irrigation using shallow (<100 m bgl) groundwaters in Bangladesh and calculated linear trends (rate of change in annual irrigation) for the period of 1985–1999. An interpolated spatial map (Figure S9) shows linear trend slopes in groundwater‐fed irrigation at the national scale, and values are extracted at each of the 2410 As sampling locations. At the national scale, magnitude of groundwater‐fed irrigation follows a similar pattern as the long‐term trends in irrigation (i.e., quantity of groundwater‐fed irrigation‐water and irrigation trends are highly correlated, Pearson's correlation 0.79). Due to this high degree of correlation, the magnitude of groundwater‐fed irrigation does not add much information to the analysis.

#### 2.2.7 Geographical, Altitudinal, and Seasonal Effects

Geostatistical analyses of the groundwater As data set in Bangladesh reveal that concentrations tend to increase from north to south following a decreasing gradient in surface elevation [*Shamsudduha*, 2007; *Shamsudduha et al*., 2009b]. To capture this systematic regional variation, we use surface elevation as a covariate, along with Legendre polynomial transformations [*Chandler*, 2005] of the geographic coordinates of As observations. The degree of polynomials is restricted to a maximum of two, because we judge that this is adequate to capture any regional‐scale variation in As concentrations that is not explained by other covariates such as geology (there will, of course, be local‐scale variation as well, but this is accounted for elsewhere as described in section 2.3.3 below). Elevation information at each As location was derived from a digital elevation model of 300 m spatial resolution [*Shamsudduha et al*., 2009b]. Additionally, to adjust for any potential seasonal variations in groundwater As concentrations since sampling was conducted over a period from January 1998 to December 1999, sampling date is incorporated into the analysis via Fourier covariates [see *Chandler and Scott*, 2011, section 3.2], specifically cos(2π × day of sampling/365) and sin(2π × day of sampling/365). Although no clear seasonal variation in As concentration has been reported in Bangladesh [*Dhar et al*., 2008], there is a possibility that seasonality may arise due to dependence on one or more seasonally varying factors that are not considered in our analysis. Our inclusion of these Fourier covariates is intended to account for any such effects if they exist, thus eliminating any potential bias that could arise due to differences in sampling dates in different regions of the country.

### 2.3 Generalized Regression Model for as in Groundwater

To investigate the simultaneous effect of the preceding factors upon groundwater As concentration at the national scale, we develop a statistical model that can be regarded as a generalized regression technique. In the first instance, this requires careful consideration of the distribution of the As concentration data. In applied literature, it is common to analyze the logarithms of the data under the assumption of normality [*Lee and Helsel*, 2007]—this procedure effectively assumes that the original data values are drawn from lognormal distributions. In the statistical literature, however, this kind of approach has been superseded by the development of the generalized linear model (GLM) which avoids the need for any data transformation. GLMs [*McCullagh and Nelder*, 1989] extend the classical linear regression model to relate the expected value of a response variable, considered to be generated from some family of probability distributions, to a linear combination of covariates. In this framework, the most common candidate distribution for modeling a skewed data set like the groundwater As concentrations in Bangladesh is the gamma [*Chandler*, 2005; *Yan et al*., 2006]; however, it is computationally intensive to handle censored data correctly in this case [*Chandler and Wheater*, 2002]. A third possibility is to use the Weibull family of distributions, which is much more tractable [*Aiken and West*, 1991] and widely used in many other applications where censoring is a problem [e.g., *Klein and Moeschberger*, 2003]. For practical purposes, the three distributional families (lognormal, gamma, and Weibull) are hard to distinguish empirically and will usually yield similar results so that the precise choice is relatively unimportant in terms of substantive conclusions [see, e.g., *Yan et al*., 2006]; although, of course, it is necessary to check that the chosen family of distributions does indeed fit the data (see *McCullagh and Nelder* [1989], for more discussion of these issues). As part of our exploratory analyses, we considered both lognormal and Weibull distributions to model As in groundwater and found that results are indeed comparable (results are not shown but modeling codes and data sets are provided as supporting information). In view of this, coupled with the need to handle censoring efficiently, our subsequent modeling is based on the Weibull distribution. We refer to our model as a “generalized regression model (GRM)” rather than a GLM because, strictly speaking, the Weibull falls outside the class of distributions for which GLMs may be defined [*Faraway*, 2006]; in terms of interpretation however, the distinction is purely semantic. Since the ideas are relatively unfamiliar in water research, we now present them in some detail.

#### 2.3.1 Modeling Framework

The problem of censoring is not unique to environmental applications: it often occurs in the biomedical sciences as well. For example, one might be interested in the age at onset of a particular disease but an individual may already have the disease at the start of a study. In this case, and in the absence of further information, we know only that the age of onset is less than the individual's current age. This is directly analogous to an As observation recorded as “below detection limits”: in both cases, although the exact value is unavailable we know it is less than some threshold, and this information can be exploited in the analysis. Survival regression models [*Aitkin and Clayton*, 1980] are designed for use in this type of situation: the terminology comes from the fact that the methods were originally developed for the modeling of lifetimes, although they are generally applicable to any situation involving censored data. Models that exploit features of the Weibull distribution to handle censoring are used widely in the biomedical sciences, although they have not yet been used widely in environmental applications [*Helsel*, 2006; *Ryberg and Vecchia*, 2006].

Similar to the gamma distribution, the Weibull distribution is a two‐parameter continuous probability distribution with parameters *α* and *λ* representing “shape” and “scale,” respectively. The probability density function (PDF) of the distribution is

(1) $$f(y;\alpha ,\lambda )=\frac{\alpha }{\lambda }{\left(\frac{y}{\lambda }\right)}^{\alpha -1}\cdot e^{-\left(\frac{y}{\lambda }\right)}{}^{\alpha }\;\text{when}\;y\geq 0;\alpha ,\lambda >0$$

and the corresponding cumulative distribution function (CDF) is

(2) $$F(y;\alpha ,\lambda )=1-e^{-\left(\frac{y}{\lambda }\right)}{}^{\alpha }\;\text{when}\;y\geq 0;F(y;\alpha ,\lambda )=0\;\text{when}\;y<0$$

The mean and variance of the Weibull distribution are $\mu =\lambda \Gamma (1+\alpha^{-1})$ and $\lambda^{2}\Gamma (1+2/\alpha )-\mu^{2}$, respectively, where Γ(•) denotes the gamma function.

In the statistical modeling framework used here, the groundwater As observations, represented by $y_{1},...,y_{n}$, are all considered to be generated from Weibull distributions with a common shape parameter *α* [*Yan et al*., 2006]. The common shape parameter implies that the As observations are all drawn from distributions with a common coefficient of variation. The scale parameters are however covariate dependent: the scale parameter for the *i*th observation is λi so that $y_{i}∼Wei(\alpha ,\lambda_{i})$.

Suppose a groundwater As concentration at each location, $y_{i}$, is to be predicted from values of *J* covariates by $\left\{x_{i}(j):j=1,...,J\right\}$. It is common practice in Weibull regression [*Klein and Moeschberger*, 2003] to use a logarithmic link between the covariates and the mean, $\mu_{i}$, of the distribution:

(3) $$\log\mu_{i}=\beta_{0}+\sum_{j}\beta_{j}x_{i}(j)$$

where $\left\{\beta_{j}\right\}$ are model coefficients. The use of a logarithmic link is adopted primarily as a convenient device to guarantee that expected As concentrations {*μ_i_*} are positive according to the model [*Yan et al*., 2006]. It also ensures that the model coefficients are easily interpretable since $e^{\beta_{j}}$ is the average multiplicative effect of a unit increase in the *j*th predictor upon the expected groundwater As concentration.

A convenient feature of the Weibull model is the tractable expression in equation (2) for the CDF. Given the parameters of the distribution, it is easy therefore to calculate the probability of any observation falling above or below a particular threshold. In particular, the probability of an observation being censored (i.e., falling below the relevant detection limit) can be calculated.

Let $\delta_{i}$ be an indicator variable taking the value 1 if the As observation at the *i*th location is uncensored, and 0 if it is censored. Moreover, let $\tau_{i}$ be the detection threshold for the observation. Then, if the groundwater As observations are mutually independent, the likelihood function (*L*) for the model parameters is
(4)
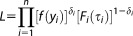



Maximum Likelihood (ML) estimation of the model parameters (i.e., the coefficients {*β_j_*} in equation (3)) can now be carried by maximizing the logarithm of equation (4) numerically [*Aitkin and Clayton*, 1980]; standard large‐sample theory can be used to calculate standard errors for the parameters and to test hypotheses about them. In reality, however, groundwater As observations from neighboring spatial locations are unlikely to be independent: the approach to deal with this difficulty is described in section 2.3.3. In this study, model fitting has been done using routines for survival analysis in the “R” environment (version 2.10.0) [*R Development Core Team*, 2009]; specifically, the survreg () function within the “survival” package [*Therneau et al*., 1990; *Therneau*, 2009] and the psm () function from the “rms” package [*Harrell*, 2001, 2012]. Our R codes and data sets are provided in supporting information.

A further potential difficulty with the development of our GRM is that several of the covariates are highly correlated: for example, covariates relating to irrigation intensity will be strongly related to hydraulic conductivity for the simple reason that permeable soils require more irrigation. This correlation makes it difficult to disentangle the effects of the individual covariates: specifically, it reduces the precision with which any individual model coefficient can be estimated [*Fox*, 2002]. However, this is automatically accounted for in the calculation of standard errors for the coefficient estimates. By considering all of the relevant factors simultaneously, the GRM provides a realistic representation of the uncertainty that accrues from correlation between covariates: thus, we can be confident that the associations revealed by the GRM are genuine.

#### 2.3.2 Model Checking and Testing

To check the fit of the GRM and to assess the appropriateness of the Weibull distribution assumption, standardized deviance residuals [*McCullagh and Nelder*, 1989; *Therneau et al*., 1990] are computed. Under the assumed model, the deviance residuals should be roughly normally distributed around a mean of 0 and with a standard deviation of 1; thus, approximately 95% of them should lie between −2 and +2 [*Chandler*, 2005], although heavy censoring (31%) in the data set can distort the normal approximation [*Davison and Gigli*, 1989].

Statistical significance of covariates is tested with a log likelihood ratio (LR) test (similar to the ANOVA test) [*Chandler and Scott*, 2011], adjusted for intersite dependence as described below. The adjusted LR test compares two models where one is a special case of the other (obtained, for example, by deleting a term or a block of terms); the test yields a *P* value for testing the hypothesis that the data were generated from the simpler of the two models. Additionally, the psm() function for GRM provides a generalized $R^{2}$ statistic commonly applied in the survival analysis to measure the proportion of variation in the response variable explained by the fitted model [*Nagelkerke*, 1991; *Harrell*, 2001]. Competing models are often compared using information criteria such as AIC (Akaike Information Criterion) and BIC (Bayesian Information Criterion). These criteria are not, however, valid in the present context because they assume that the likelihood function used to fit the models is correct. At an operational level, our adjusted LR test can be regarded as similar in spirit to the use of AIC and BIC but has the advantage that it accounts explicitly for unmodeled intersite dependence.

#### 2.3.3 Intersite Dependence

In section 2.3.1, we explained how model parameters can be estimated using ML under the assumption that observations in the data set are independent. In reality, however, unmodeled local‐scale controls are likely to produce dependence between neighboring locations as described in section 2.1. One possible approach to this problem is to model the dependence explicitly and to incorporate it into the ML fitting procedure. The computational burden of such an approach is formidable, however, and most existing techniques for this type of problem [e.g., *Banerjee et al*., 2004] are designed for use when the response variable is normally distributed, which is not the case here. A further drawback is that the approach can produce biased estimates of the regression coefficients, particularly (but not exclusively) if the intersite dependence structure is misspecified [*Pepe and Anderson*, 1994; *Park and Kim*, 2004; *Qu and Song*, 2004]. By contrast, “independence” ML estimates are unbiased in the presence of unmodeled dependence—although it is necessary to adjust their standard errors, along with likelihood ratios for comparing models [*Chandler*, 2005]. We adopt this here as a pragmatic, but theoretically defensible, approach to the problem.

The required adjustments to the standard errors can be calculated in a relatively straightforward manner if the As observations can be separated into a large number of distinct subsets that can be considered as independent [*Chandler and Bate*, 2007]. To achieve this, we fitted a preliminary model similar to that discussed below, and plotted a variogram of the residuals from this model (see section 3). This variogram indicated that spatial dependence was relatively localized and that sites separated by more than ∼25 km could be considered as effectively independent. We therefore sought a means of grouping the sites into a large number of subsets, in such a way that no two sites in different subsets are within 25 km of each other. To attempt this manually is infeasible; instead, we used a hierarchical clustering algorithm based on the geographical site coordinates. Ideally, we would like to obtain subsets that are geographically as widely separated from each other as possible; thus, a single‐linkage algorithm was used in the first instance so as to maximize the smallest distance between sites in distinct subsets [*Romesburg*, 2004]. However, some of the subsets generated by this procedure were very large due to the effect known as “chaining” [*Hartigan*, 1981]. To deal with this, subsets containing more than 50 sites were further split by running the Ward‐linkage method which tends to create subsets of more uniform size. Finally, sites within 25 km of another subset were removed one at a time until nearly all subsets were separated by at least 25 km. In this process, 767 sites were removed in total: these were not used for model calibration but were retained for subsequent validation. Data from the remaining 1643 sites (calibration data set), forming a total of 212 independent subsets (Figure S10), were used to fit the model. The theory described in *Chandler and Bate* [2007] was then used to adjust standard errors and likelihood ratios for the within‐subset dependence in the calibration data. A brief summary of the theory is provided in Appendix A of the supporting information.

#### 2.3.4 Statistical Interactions

Covariates may interact with each other so that the effect of one covariate upon the response variable may depend on the values of others. For example, previous studies [*DPHE*, 1999; *Ravenscroft*, 2001] examined statistical associations between aquifer's hydraulic properties and groundwater As concentrations and suggested that the relationship varies within different geological units in Bangladesh. As another example, consider two geological units, one containing Fe‐oxyhydroxide minerals whose dissolution would lead to the mobilization of As in groundwater and the other containing no such minerals. In this case, one might expect groundwater As concentrations to show a significant association with aquifer recharge in the first unit, but not necessarily any association in the second. Thus, the association between groundwater recharge and As concentration varies between geological units, resulting in a statistical interaction.

The presence of interactions can have important implications for the interpretation of statistical models [*Aiken and West*, 1991]. Moreover, they are easily handled within any regression framework, including the survival regression models considered here. All that is required is the addition of an extra term to the model, which is a product of the interacting covariates [*Chandler and Wheater*, 2002]. In the present study, several statistical interactions are included between covariates along with their main effects to explain variability in As observations. Exploratory analyses reveal substantial variations in relationships between groundwater As concentrations and mean annual recharge (PGI period) within different geological units in Bangladesh (Figure S11). Similar relationships exist between groundwater As and sampling depths (Figure S12), and between groundwater As and the thickness of surficial silt and clay (TSSC) (Figure S13). Therefore, the covariates (mean groundwater recharge for PGI period, well depth, and TSSC) and their interactions with the surface geology are considered in the model.

## 3 Results

### 3.1 Model Fitting

Fitting a generalized regression model (GRM) with the Weibull distribution involves choosing the covariates and estimating the corresponding parameters. Table S3 summarizes the basic statistics of covariates used in the GRM. Model building was done in stages, starting with a basic model including functions of spatial location (latitude, longitude, and surface elevation) along with sine and cosine functions of sampling date to represent any potential seasonal variation. Additionally, the variation in As concentrations with sampling depth was represented by the intake depth of each surveyed well. Subsequently, the factors representing surface geology, hydrogeology, groundwater dynamics, recharge processes, and abstraction were sequentially added to the model together with the associated statistical interaction terms. To represent the effect of the distinct geological units upon As concentrations within the fitted GRM, it is necessary to regard the surface geology (*K* = 15) as a categorical covariate [*Davison*, 2003]. The effects of such covariates can be represented using a separate coefficient for each of the *K* levels; however, only *K*−1 of these coefficients are identifiable [*Hardy and Reynolds*, 2009]. It is therefore conventional to impose a constraint to overcome this problem and for software to report only *K*−1 of the coefficients. Here, we have constrained the coefficients to sum to zero in the fitted model; the effect is that all of the other terms can be interpreted as representing “overall average” relationships across the whole of Bangladesh. All terms were added to the model, and the model fit was assessed by examining the standardized deviance residuals. At this stage, all the covariates listed in supporting information (see Table S3) have been added regardless of their apparent statistical significance: the resulting (comprehensive) model is deliberately overfitted. The comprehensive, national‐scale GRM has a total of 16 covariates and 3 statistical interactions. Subsequently, adjusted log likelihood ratio (LR) tests were applied to assess the significance of these covariates in explaining the overall As variation in groundwater. The LR test for individual covariates was performed by (1) fitting a simpler model in which the corresponding term(s) (including any statistical interactions) were omitted, and (2) comparing the adjusted log likelihoods of the full and simplified models. Lastly, we develop a final, national‐scale GRM after systematically discarding a number of covariates and their associated terms from the comprehensive model; this is done sequentially, so that after deleting each term or group of terms the statistical significance of the remaining terms is reassessed by LR test (see section 3.3). The resulting final GRM, which has a total of 10 covariates and 3 statistical interactions, is simpler and outcomes are easier to interpret.

### 3.2 Model Diagnostics

It is necessary to check the fit of any statistical model before interpreting the result. Checks of statistical models primarily involve (i) diagnostics of the model structure, (ii) examination of the assumed probability distribution, and (iii) assessment of the predictive ability [*Chandler and Wheater*, 2002]. In addition to these standard checks, for spatial models it is often informative to construct a variogram of the model residuals to assess the strength of intersite dependence. Figure S14 shows the spatial distribution of the standardized deviance residuals from the fitted GRM, whereas the variogram in Figure S15 shows their spatial dependence. It is evident from these plots that there is little spatial organization in the residuals except for intersite dependence up to a distance of 0.25° (∼25 km). Results show that approximately 96% of the deviance residuals fall between −2 and 2, as expected under the model.

It is also necessary to ensure that the probability structure (i.e., the Weibull distribution assumption in the current context) of the fitted model is correct since this is used to compute the likelihoods upon which inferences are based [*Chandler and Wheater*, 2002]. In survival regression, in the presence of censoring, the assumption for the Weibull distribution in the fitted model is generally checked visually by plotting $\log[-\log(1-F(\tau_{i}))]$ against log⁡(τ) [*Kleinbaum and Klein*, 2005; *Therneau*, 2009]; a straight line plot (Figure S16) indicates that the choice of Weibull distribution is reasonable here. Although a few points in the lower tail of the distribution fall slightly away from the straight line, these lie within the 95% uncertainty envelopes on the plot: this indicates that the departure from linearity is within the expected magnitude of sampling variation.

The predictive ability of the resulting model has also been checked using the 767 As observations that were not used in model calibration. The validation of the fitted model yields comparable residuals with the mean of −0.16 and standard deviation of 1.15. Few standardized deviance residuals (2.6%; 20 locations) are larger than 2 of which the average observed As concentration is high (mean As concentrations of 267 μg L^−1^ from 20 observations). Additionally, the spatial distribution of the deviance residuals for the validation data set (Figure S14) compares well with that of the calibration data. Similar to the calibration model, a log‐log plot (Figure S16) shows that the assumption for the Weibull distribution is valid. Overall, these diagnostic analyses indicate that the modeling results are reproducible.

### 3.3 Model Outputs and Interpretation

The GRM describes the variation in As concentrations and its relationship with surface geology, and hydrogeological processes that can influence As mobilization in shallow groundwaters. The resulting national‐scale GRM has a total of 16 covariates and 3 statistical interactions among surface geology and other covariates resulting in 76 model terms. Key model results are summarized in Tables S4 and S5 for the national‐scale and regional‐scale GRMs respectively. Because the GRM uses a logarithmic link between covariates and As concentrations (equation (3)), each exponentiated model coefficient ( $e^{\beta_{j}}$) is the average multiplicative effect of a one unit increase in the value of the corresponding covariate upon the mean As concentration. For example, the coefficient of −0.023 associated with hydraulic conductivity implies that an increase in hydraulic conductivity of 1 m d^−1^ is associated with a multiplicative change of exp(−0.023) = 0.977 (or, equivalently, a 2.3% decrease) in mean As concentration. The interpretation of model coefficients for the thickness of surficial silt and clay (TSSC) and mean groundwater recharge is different, however, because these covariates have interaction terms with surface geology so that their effects are geology specific. Their multiplicative effects on mean As concentration must therefore be calculated using both “main effect” and interaction coefficients. For example, in the deltaic sand (deltaic sand or “dsd”; see supporting information Figure S3 for full names of geological units) unit, an increase of TSSC by 1 m implies a proportional increase of mean As concentration by exp(−0.025 + 0.148) or 13% when all other covariates remain unchanged. By contrast, in the valley alluvium/colluvium (“ava”) unit, the corresponding proportional change is exp(−0.025 − 0.1136): here then, the same increase of TSSC implies a 13% decrease in mean As concentration. Detailed model outputs are provided in the supporting information (Tables S4 and S5).

Results from the national‐scale GRM show that surface geology, hydraulic conductivity, mean groundwater recharge, recharge trends, and groundwater‐fed irrigation trends all influence the spatial variation in groundwater As concentrations. The most important factors in the model are surface geology and its statistical interactions with well depth, PGI mean groundwater recharge, and TSSC. Based on a generalized *R^2^* value (see section 2.3.2), these factors alone explain ∼43% of the spatial variation in groundwater As concentrations. In contrast, aquifer's specific yield, Darcy velocity, and seasonal groundwater levels are the least important factors.

To assess the importance of individual covariates or groups of covariates in describing As variations in shallow groundwater, simpler models have been fitted in which individual terms or groups of terms were omitted from the full, national‐scale GRM; the explanatory power of the omitted terms was then assessed using an adjusted likelihood ratio (LR) test of the full versus reduced models. A summary of the LR test statistics for each model factor is given in Table 1. Recall, from section 2.3.2, that the null hypothesis for the LR test is that the data were generated from the reduced model: a low *P* value, leading to rejection of this hypothesis, can therefore be taken as evidence that the omitted terms are necessary and hence that the associated covariates are genuinely associated with variations in As concentrations. These results show that dropping some factors can significantly reduce the predictive capacity of the fitted model, whereas for others there is no significant change. For example, surface geology and its statistical interactions with TSSC, well depth, and mean PGI recharge are the most significant (LR‐test *P* value close to 0, see Table 1) factors explaining As variations. Likewise, the predictive power of the fitted GRM reduces when hydraulic conductivity, groundwater‐level trends, net changes in recharge and irrigation trends are omitted. Dropping other covariates such as specific yield, Darcy flux, GWT‐wet, and surface elevation does not affect the overall fit of the model; these variables can thus be considered as largely irrelevant in explaining spatial variations in As concentrations at national or regional scales.

**Table 1:** Effect of Dropping Covariates and Their Associated Terms From the Comprehensive, National‐Scale GRM According to Adjusted Log Likelihood Ratio (LR) Test Procedures^a^

Covariates/Factors	DF	LR Test *P* Value (Adjusted)
*Geology and Hydrogeological Variables*		
Surface geology and all statistical interactions	56	0
TSSC and its interaction with surface geology	1	5.2 × 10^−4^
Hydraulic conductivity	1	0.0334
Specific yield	1	0.6129
Darcy flux	1	0.6633
Well depth and interaction	15	8.3 × 10^−5^
*Hydrodynamic and Groundwater Recharge Variables*		
Wet‐season groundwater table (GWT‐wet)	1	0.9914
Groundwater‐level trends	1	0.0754
Mean groundwater fluctuation	1	0.3436
Mean PGI recharge and statistical interaction	15	0.0073
Net changes in recharge	15	0.0016
*Geographical, Altitudinal, and Seasonal Factors*		
Geographic coordinates (longitudes and latitudes) and statistical interactions	7	0.0008
Surface elevation	1	0.9147
Seasonality (sine + cosine of sampling dates)	2	0.5839
Geographic coordinates and interaction, surface elevation, and seasonality terms	10	0.0008
*Groundwater‐Fed Irrigation*		
Irrigation trends (1985−1999)	1	0.1470
Irrigation trends and net recharge changes	2	0.0011
Irrigation trends, mean PGI recharge and its interaction, and net recharge changes	17	3.8 × 10^−7^
*Statistical Interaction Terms*		
Only geological interaction terms	42	0

a *P* values derived from LR test and any value less than 10^−10^ is reported as zero. DF denotes degrees of freedom.

**Figure 4:**
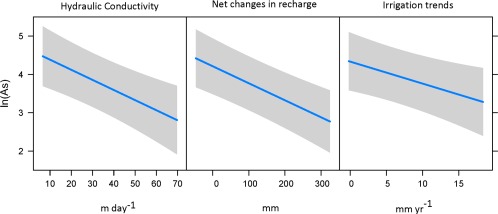
Modeled effects of key covariates: hydraulic conductivity, net changes in recharge, and irrigation trends on As concentrations in the national‐scale, final GRM. On each plot, the vertical axis is labeled as contribution to the natural logarithm of expected As concentration, the horizontal axis denotes measurement unit for each covariate, and a 95% pointwise confidence interval is drawn around the estimated effect. Note: relationships for hydraulic conductivity and net changes in recharge are statistically significant (LR‐test *P* values < 0.05), whereas the relationship for irrigation trends is of borderline significance (LR‐test *P* value of 0.06).

The final, national‐scale GRM is simpler yet retains a total of 10 statistically significant covariates and 3 interactions with surface geology and covariates (well depth, TSSC, and mean PGI recharge) (see supporting information for detailed model outputs). Figure 4 shows the modeled associations between three key hydraulic and groundwater abstraction covariates (hydraulic conductivity, net changes in recharge and irrigation trends) and As concentrations in groundwater. Modeled coefficients of the key covariates between two national‐scale GRMs (comprehensive and final) are comparable (see Table 2 and supporting information Table S4). In the final GRM, statistically significant (LR‐test *P* values <0.05) negative coefficients of −0.026 and −0.004 are obtained for hydraulic conductivity and net changes in recharge respectively; a negative coefficient of −0.056 for irrigation trends is of borderline significance (LR‐test *P* value of 0.06). However, modeled associations of the combined effect of covariates grouped under groundwater‐fed irrigation in Table 1 (e.g., irrigation trends, mean PGI recharge and its interaction, and net recharge changes) are statistical significant (LR‐test *P* value <10^−6^). As described above, the coefficient of −0.026 for hydraulic conductivity implies a 2.6% reduction in expected As concentrations for each unit (m d^−1^) increase in hydraulic conductivity. Similarly, a unit (mm) increase of net change in recharge is associated with a 0.4% reduction in expected As concentration; and a unit (mm yr^−1^) increase of irrigation trend (1985–1999) is associated with a 5.6% reduction. Operationally of course, these effects do not act in isolation: however, the GRM provides a means of quantifying the relative contributions of these different factors to the variation in As concentrations. The absence of significant statistical interactions between these factors and other covariates is particularly noteworthy as this indicates that the modeled relationships are consistent across the range of hydrogeological conditions represented in the data set.

**Table 2:** Summary of Key Results From the Final, National‐Scale GRM^a^

Covariates	Unit	Coefficient	Std. Error	LR Test *P* Value
*Geological and Hydrogeological Variables*				
Surface geology (*n* = 15)^b^	categorical			
TSSC and its interaction with surface geology^c^	m	−0.023	0.019	0.0013
Hydraulic conductivity	m d^−1^	−0.026	0.007	0.0089
Sampling well depth and its interaction with surface geology^c^	m	−0.012	0.009	0.0001
*Hydrodynamic and Groundwater Recharge Variables*				
Mean groundwater recharge and its interaction with surface geology^c^	mm yr^−1^	−0.0006	0.004	0.0019
Net changes in recharge^d^	mm	−0.004	0.0009	0.0006
Mean groundwater‐level trends	cm yr^−1^	0.034	0.012	0.0230
*Groundwater‐Fed Irrigation*				
Irrigation trends (1985−1999)	mm yr^−1^	−0.056	0.022	0.0635

a Model coefficients represent the overall effect of a covariate excluding its statistical interactions; however, *P* values are for the main effect together with all associated interactions. Complete results from the national‐scale, final GRM are provided in supporting information.

b Model coefficients, standard errors, and *P* values for categorical surface geology covariate listed in the supporting information.

c Model coefficients are associated with the respective covariate only and not including their statistical interactions.

d Net changes in mean recharge between PGI (1975−1980) and DGI (1995−1999) periods.

## 4 Discussion

### 4.1 Robustness of Modeled Associations in GRMs

It can be argued that the national‐scale GRM is biased toward the predominantly low‐As concentrations observed in groundwater in the Pleistocene and older deposits in Bangladesh. To check the robustness of modeled associations, we constructed a reduced model (regional‐scale GRM) restricting data to the As‐affected geological units in Bangladesh (i.e., excluding pre‐Holocene deposits). These pre‐Holocene deposits are the Barind clay residuum (“rb”), Madhupur clay residuum (“rm”), and pre‐Quaternary bedrock (“br”) deposits. Previous studies [*DPHE*, 1999; *BGS and DPHE*, 2001; *McArthur et al*., 2008, 2011b] have argued that mobile As from the Plio‐Pleistocene sediments in Barind and Madhupur Tracts (Figures 1 and 2) was vigorously flushed during the Last Glacial Maximum (LGM) (20 *ka*) under greater hydraulic gradients. Consequently, observed As concentrations in groundwater within these geological units are consistently low (<10 μg L^−1^). In contrast, prior to recent abstraction for irrigation, mobile As in shallow groundwater within the Holocene deposits was not subjected to vigorous flushing under natural (i.e., nil pumping) hydraulic gradients [*Ravenscroft et al*., 2005]. Intensive groundwater pumping for irrigation over the last few decades has, however, perturbed natural hydraulic gradients in shallow aquifers [*Harvey et al*., 2006; *Klump et al*., 2006; *Michael and Voss*, 2009]. We thus investigated whether the statistical associations observed in the national‐scale GRM hold in the regional‐scale GRM restricted to the Holocene deposits where As concentrations in shallow groundwater are generally high. Our analysis of the regional‐scale GRM confirms that the modeled associations of critical covariates explaining the spatial variation in groundwater As concentrations such as irrigation trends, net changes in recharge and hydraulic conductivity are robust and similar to the outcomes of both comprehensive and final national‐scale GRMs (see Table 2 and supporting information Tables S4 and S5).

### 4.2 Effects of Irrigation‐Induced Recharge on As Cycling

It has been argued that groundwater recharge can dilute previously mobilized As over time through flushing [*McArthur et al*., 2004, 2011a]. This implies that lower As concentrations would occur in areas where groundwater abstraction for irrigation has induced greater recharge to shallow aquifers. Our models reveal negative associations between irrigation trends, net changes in recharge and As concentrations in shallow groundwater. Both national and regional‐scale GRMs (Tables 1 and 2) show that an average net change in groundwater recharge of 1 mm yr^−1^ is associated with 0.4% lower mean As concentration (e.g., national mean As concentration 62 μg L^−1^). Net changes in recharge in the order of 100 mm yr^−1^ between the predeveloped groundwater‐fed irrigation (1975−1980) and developed groundwater‐fed irrigation (1995−1999) have been observed in Bangladesh [*Shamsudduha et al*., 2011]. According to the GRMs such a net change (100 mm yr^−1^) in mean groundwater recharge implies a 40% lower mean As concentration. This relationship inferred from our statistical model is one of association rather than causation; nonetheless, our modeling results are clearly consistent with the assertion that irrigation‐induced recharge serves to flush mobile As from shallow groundwater.

The mobilization of As in shallow groundwater, however, depends on a range of geochemical factors that include retardation (i.e., adsorption capacity of oxidized brown sediments) [*Radloff et al*., 2011], rates of reductive dissolution of iron‐oxyhydroxide minerals [*Nickson et al*., 1998], and the availability of reactive organic carbon (OC) in aquifers to drive microbial metabolism [*Harvey et al*., 2006; *Neumann et al*., 2010]. These factors vary locally and can greatly influence local‐scale variations in As concentrations in shallow aquifers. Indeed, at local scales, it has been argued that irrigation‐induced recharge both decreases the concentration of mobile As by flushing [*McArthur et al*., 2004; *Stute et al*., 2007; *van Geen et al*., 2008] and increases the mobilization of As by transporting reactive OC from surface sources to the site of As release [*Harvey et al*., 2006; *Neumann et al*., 2010]. Recent evidence from short‐term (<10 years) monitoring of As concentrations at sites in the Bengal Basin [*McArthur et al*., 2010; *Bhattacharya et al*., 2011] and other Asian Mega‐Deltas [*Winkel et al*., 2011; *van Geen et al*., 2013] suggests that As concentrations have decreased in areas of active flushing of aquifer due to irrigation‐induced recharge but increased in areas where intensive, long‐term pumping has transported groundwater from As‐contaminated regions in the aquifer. Our statistical models do not attempt to explain such localized variations in groundwater As concentrations; they reveal overall, mean relationships between As and covariates at both national and regional scales. As such, they explain large‐scale structures while implicitly accounting for localized variations by adjusting for the induced intersite dependence when calculating standard errors and performing hypothesis tests. Further sampling of shallow groundwater throughout Bangladesh is required to confirm the implications of the GRMs.

**Figure 5:**
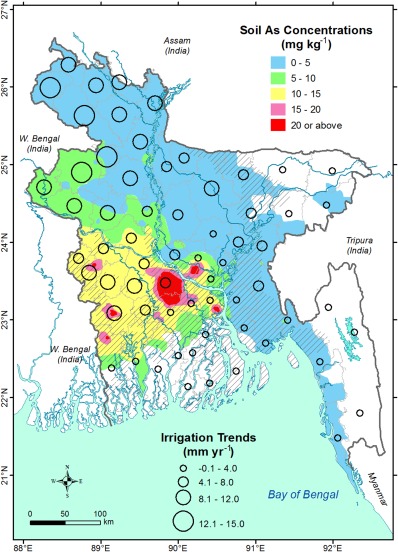
Average groundwater‐fed irrigation trends (mm yr^−1^) for the period of 1985–1999 in each districts (*n* = 64) in Bangladesh. The background map shows observed soil (depth 0−15 cm bgl) As concentrations surveyed and interpolated from a total of 394 sampling points reported in *Duxbury and Panaullah* [2007]. Areas in Bangladesh where As concentrations in groundwater are >50 μg L^−1^ are shown as hatched symbol.

A critical water management concern in Bangladesh is whether irrigation with As‐contaminated groundwater redistributes As from shallow aquifers to soil thereby affecting food security and human health [*Meharg and Rahman*, 2003; *Williams et al*., 2006; *Ravenscroft et al*., 2009]. Our statistical models cannot directly investigate this assertion, but modeled negative associations between As concentrations, irrigation trends and net changes in recharge can explain the process of As cycling. A national‐scale map of soil (sampling depth 0−15 cm bgl) As concentrations (Figure 5) surveyed between 2001 and 2005 throughout Bangladesh [*Duxbury and Panaullah*, 2007] shows that low As concentrations in soil (<5 mg kg^−1^) occur in northwestern and north‐central Bangladesh whereas higher concentrations of As in soils (>15 mg kg^−1^) are found south of the River Ganges. The survey also reveals that subsoil (30−45 cm bgl) As concentrations are much higher (5−15 mg kg^−1^) to the south of the River Ganges than in the rest of Bangladesh [*Duxbury et al*., 2011]. These observations suggest that As, if it was present in groundwater of northwestern and north‐central Bangladesh, has not recently been removed through irrigation‐induced groundwater recharge but may have previously been flushed during the LGM [*McArthur et al*., 2008]. South of the River Ganges, high As concentrations (>20 mg kg^−1^) in soils coincide with the areas where increases in groundwater‐fed irrigation have taken place in the last two decades (Figure 5). *Neumann et al*. [2010, 2011] show further that in Mushiganj very little (<10%) of the As applied to rice fields through groundwater‐fed irrigation returns to the aquifer. This region includes areas where intensive pumping for dry‐season irrigation has induced greater groundwater recharge to shallow aquifers, and areas (e.g., south of the confluence of the Rivers Ganges and Brahmaputra) where rain‐fed recharge to the underlying aquifer is inhibited by low permeability of surface geology [*Shamsudduha et al*., 2011].

## 5 Concluding Remarks

This study has two major outcomes. First, we demonstrate the application of generalized regression models (GRMs) to explain the spatial variation in groundwater As data set in Bangladesh that features (1) a highly skewed distribution, (2) a substantial number censored or nondetect observations, and (3) correlations between sites from neighboring spatial locations. These characteristics are commonly observed in many hydrological and environmental data sets where GRMs can be used to explain the spatial variation of variables of interest.

Second, we observe statistically significant inverse associations between groundwater As concentrations and two covariates: (1) hydraulic conductivity of the shallow aquifer and (2) net increase in mean recharge between periods before and after the development of groundwater‐fed irrigation. A sensitivity analysis using an adjusted log likelihood ratio (LR) test shows that a combined effect of groundwater‐fed irrigation trends (1985–1999) and recharge factors on the variation of As concentrations is statistically significant (LR‐test *P* value < 10^−6^). These associations are observed both nationally and in a regional‐scale model restricted to aquifers in Holocene deposits where As concentrations in shallow groundwater are generally high (>10 μg L^−1^). Both GRMs demonstrate significant associations between surface geology and As concentrations. The GRMs further reveal that significant statistical interactions exist between surface geology and mean groundwater recharge, thickness of surficial silt and clay (TSSC) and well depth.

Since time series records of As concentrations in shallow groundwater are limited, our analysis reveals associations rather than causation: further surveys are required to test the mechanisms implied by the associations that our GRMs reveal. A further caveat is that our analysis has necessarily used interpolated values of some covariates without explicitly accounting for the associated uncertainty; the relationships we have found for these covariates are therefore probably weaker than those that would have been obtained if they had been observed perfectly. A pragmatic interpretation of this is: where we find statistically significant associations involving the interpolated covariates, we can be confident that there are genuine associations with the true covariates as well.

Groundwater‐fed irrigation in Bangladesh has been observed to lower water tables during the dry season and induce greater recharge during the subsequent monsoon by increasing available groundwater storage [*Shamsudduha et al*., 2011]. Statistical associations developed here indicate that where favorable geological conditions enable increased recharge capture, lower As concentrations in shallow groundwater are observed. At local scales, irrigation‐induced recharge has been shown both to decrease the concentration of mobile As by flushing [*McArthur et al*., 2004; *Stute et al*., 2007] and to increase As concentrations by transporting reactive organic carbon from surface sources to the site of As release [*Harvey et al*., 2006; *Neumann et al*., 2010]. National and regional‐scale analyses presented here reveal an overall negative association between irrigation trends, net changes in recharge, and As concentrations in shallow groundwater. These findings are consistent with the assertion that irrigation‐induced recharge serves to flush mobile As from shallow groundwater. It has been observed that very little of the As in pumped groundwater (primarily applied as dry‐season irrigation water) is returned to the underlying aquifer [*Neumann et al*., 2010, 2011]. Groundwater abstraction for irrigation may consequently redistribute As to soil where it can continue to pose a threat to human health and food security in the Bengal Basin [*Ravenscroft et al*., 2009].

## Supporting information

Supporting Information:

Shamsudduha_etal_WRR_paper_SI_Readme_file_19Nov14

R_codes_for_adjusted_LR_test_19Nov14

R_codes_for_national_scale_GRM_19Nov14

R_codes_for_regional_scale_GRM_19Nov14

Shamsudduha_etal_SI_Final_GRM_full_results_19Nov14

Shamsudduha_etal_WRR_supplementary_information_15Dec2014

Arsenic_calibration_n1643_dataset_19Nov14

Arsenic_validation_n767_dataset_19Nov14

## References

[wrcr21308-bib-0001] Ahmed, K. M. , P. Bhattacharya , M. A. Hasan , S. H. Akhter , S. M. M. Alam , M. A. H. Bhuyian , M. B. Imam , A. A. Khan , and O. Sracek (2004), Arsenic enrichment in groundwater of the alluvial aquifers in Bangladesh: An overview, Appl. Geochem., 19(2), 181–200.

[wrcr21308-bib-0002] Aiken, L. S. , and S. G. West (1991), Multiple Regression: Testing and Interpreting Interactions, SAGE, Newbury Park, Calif.

[wrcr21308-bib-0003] Aitkin, M. , and D. Clayton (1980), The fitting of exponential, Weibull and extreme value distributions to complex censored survival data using GLIM, Appl. Stat., 29, 156–163.

[wrcr21308-bib-0004] Alam, M. K. , A. K. M. S. Hasan , M. R. Khan , and J. W. Whitney (1990), Geological map of Bangladesh, Geol. Surv. of Bangladesh, Dhaka.

[wrcr21308-bib-0005] Antweiler, R. C. , and H. E. Taylor (2008), Evaluation of statistical treatments of left‐censored environmental data using coincident uncensored data sets: I. Summary statistics, Environ. Sci. Technol., 42(10), 3732–3738. 18546715 10.1021/es071301c

[wrcr21308-bib-0006] Aziz, Z. , et al. (2008), Impact of local recharge on arsenic concentrations in shallow aquifers inferred from the electromagnetic conductivity of soils in Araihazar, Bangladesh, Water Resour. Res., 44, W07416, doi:10.1029/2007WR006000.

[wrcr21308-bib-0007] BADC (2003), Survey report on irrigation equipment and irrigated area in Boro 2002 season, report Boro/2001‐2002, Surv. and Monit. Project for Dev. of Minor Irrig., Dhaka.

[wrcr21308-bib-0008] Banerjee, S. , B. P. Carlin , and A. E. Gelfand (2004), Hierarchical Modelling and Analysis for Spatial Data, CRC Press, Boca Raton, Fla.

[wrcr21308-bib-0009] BGS and DPHE (2001), Arsenic contamination of groundwater in Bangladesh, *Rep. WC/00/19*, 267 pp., Br. Geol. Surv., Keyworth, U. K.

[wrcr21308-bib-0010] Bhattacharya, P. , D. Chatterjee , and G. Jacks (1997), Occurrence of arsenic‐contaminated groundwater in alluvial aquifers from the Delta Plains, eastern India: Options for safe drinking water supply, Int. J. Water Res. Dev., 13, 79–92.

[wrcr21308-bib-0011] Bhattacharya, P. , et al. (2011), Temporal and seasonal variability of arsenic in drinking water wells in Matlab, southeastern Bangladesh: A preliminary evaluation on the basis of a 4 year study, Environ. Sci. Health, Part A, 46(11), 1177–1184. 10.1080/10934529.2011.598768 21879850

[wrcr21308-bib-0012] BWDB (1989), Report on the compilation of aquifer test analysis results, *BWDB Water Supply Pap. 502*, Ground Water Circle II, Dhaka.

[wrcr21308-bib-0013] BWDB (1994), Report on the compilation of aquifer test analysis results, *BWDB Water Supply Pap. 534*, Ground Water Circle II, Dhaka.

[wrcr21308-bib-0014] Carroll, R. J. , D. Ruppert , L. A. Stefanski , and C. M. Crainiceanu (2006), Measurement Error in Nonlinear Models: A Modern Perspective, 2nd ed., Chapman and Hall, Boca Raton, Fla.

[wrcr21308-bib-0015] Chandler, R. , and H. Wheater (2002), Analysis of rainfall variability using generalized linear models: A case study from the West of Ireland, Water Resour. Res., 38(10), 1192, doi:10.1029/2001WR000906.

[wrcr21308-bib-0016] Chandler, R. E. (2005), On the use of generalized linear models for interpreting climate variability, Environmetrics, 16, 699–715.

[wrcr21308-bib-0017] Chandler, R. E. , and S. Bate (2007), Inference for clustered data using the independence log‐likelihood, Biometrika, 94, 167–183.

[wrcr21308-bib-0018] Chandler, R. E. , and M. Scott (2011), Statistical Methods for Trend Detection and Analysis in the Environmental Sciences, John Wiley, Chichester, U. K.

[wrcr21308-bib-0019] Chowdhury, M. T. A. , A. A. Meharg , C. Deacon , M. Hossain , and G. J. Norton (2012), Hydrogeochemistry and arsenic contamination of groundwater in the Haor basins of Bangladesh, Water Qual. Exposure Health, 4, 67–78.

[wrcr21308-bib-0020] Datta, S. , A. W. Neal , T. J. Mohajerin , T. Ocheltree , B. E. Rosenheim , C. D. White , and K. H. Johannesson (2011), Perennial ponds are not an important source of water or dissolved organic matter to groundwaters with high arsenic concentrations in West Bengal, India, Geophys. Res. Lett., 38, L20404, doi:10.1029/2011GL049301.

[wrcr21308-bib-0021] Davison, A. C. (2003), Statistical Models, Stat. and Probabilistic Math. Ser., Cambridge Univ. Press, Cambridge, U. K.

[wrcr21308-bib-0022] Davison, A. C. , and A. Gigli (1989), Deviance residuals and normal scores plots, Biometrika, 76(2), 211–221.

[wrcr21308-bib-0023] Dhar, R. K. , Y. Zheng , M. Stute , A. van Geen , Z. Cheng , M. Shanewaz , M. Shamsudduha , M. A. Hoque , M. W. Rahman , and K. M. Ahmed (2008), Temporal variability of groundwater chemistry in shallow and deep aquifers of Araihazar, Bangladesh, J. Contam. Hydrol., 99(1–4), 97–111. 18467001 10.1016/j.jconhyd.2008.03.007 PMC2605690

[wrcr21308-bib-0024] DPHE (1999), Groundwater studies for arsenic contamination in Bangladesh, Rapid Investigation Phase, Main Report, vol. S1–S5, Br. Geol. Surv., Keyworth, and Mott MacDonald Ltd., U. K.

[wrcr21308-bib-0025] Duxbury, J. M. , and G. Panaullah (2007), Remediation of arsenic for agriculture sustainability, food security and health in Bangladesh, FAO Working Paper, FAO, Rome.

[wrcr21308-bib-0026] Duxbury, J. M. , G. M. Panaullah , Y. J. Zavala , R. H. Loeppert , and Z. U. Ahmed (2011), Impact of use of As‐contaminated groundwater on soil As content and paddy rice production in Bangladesh, in *Issues in Asian Agriculture, Tech. Bull*, Food & Fertilizer Technology Center, [Available at http://www.agnet.org/htmlarea_file/library/20110808101120/tb180.pdf.]

[wrcr21308-bib-0027] Faraway, J. (2006), Extending the Linear Model With R: Generalized Linear, Mixed Effects and Nonparametric Regression Models, Chapman and Hall, Boca Raton, Fla.

[wrcr21308-bib-0028] Fendorf, S. , H. A. Michael , and A. van Geen (2010), Spatial and temporal variations of groundwater arsenic in south and southeast Asia, Science, 328(5982), 1123–1127. 20508123 10.1126/science.1172974

[wrcr21308-bib-0029] Fox, J. (2002), An R and S‐Plus Companion to Applied Regression, SAGE, Thousand Oaks, Calif.

[wrcr21308-bib-0030] Gaus, I. , D. G. Kinniburgh , J. C. Talbot , and R. Webster (2003), Geostatistical analysis of arsenic concentration in groundwater in Bangladesh using disjunctive kriging, Environ. Geol., 44, 939–948.

[wrcr21308-bib-0031] Hardy, M. A. , and J. Reynolds (2009), Incorporating categorical information into regression models: The utility of dummy variables, in Handbook of Data Analysis, edited by Hardy M. A. and Bryman A., p. 728, SAGE, Thousand Oaks, Calif.

[wrcr21308-bib-0032] Harrell, F. E. (2001), Regression Modeling Strategies, With Applications to Linear Models, Survival Analysis and Logistic Regression, Springer, N. Y.

[wrcr21308-bib-0033] Harrell, F. E. (2012), *Rms Package, Regression Modeling Strategies*, version 3.5‐0, R Dev. Core Team. CRAN. [Available at http://biostat.mc.vanderbilt.edu/rms.]

[wrcr21308-bib-0034] Hartigan, J. A. (1981), Consistency of single linkage for high‐density clusters, J. Am. Stat. Assoc., 76, 388–394.

[wrcr21308-bib-0035] Harvey, C. F. , et al. (2006), Groundwater dynamics and arsenic contamination in Bangladesh, Chem. Geol., 228, 112–136.

[wrcr21308-bib-0036] Helsel, D. R. (2005), Nondetects and Data Analysis: Statistics for Censored Environmental Data, John Wiley, N. Y.

[wrcr21308-bib-0037] Helsel, D. R. (2006), Fabricating data: How substituting values for nondetects can ruin results, and what can be done about it, Chemosphere, 65(11), 2434–2439. 16737727 10.1016/j.chemosphere.2006.04.051

[wrcr21308-bib-0038] Helsel, D. R. (2010), Much ado about next to nothing: Incorporating nondetects in science, Ann. Occupational Hyg., 54(3), 257–262. 10.1093/annhyg/mep092 20032004

[wrcr21308-bib-0039] Hiscock, K. M. (2005), Hydrogeology: Principles and Practice, Blackwell Sci., Oxford, U. K.

[wrcr21308-bib-0040] Katz, R. W. , M. B. Parlange , and C. Tebaldi (2003), Stochastic modeling of the effects of large‐scale circulation on daily weather in the southeastern US, Clim. Change, 60, 189–216.

[wrcr21308-bib-0041] Klein, J. P. , and M. Moeschberger (2003), Survival Analysis Techniques for Censored and Truncated Data, 2nd ed., Springer, N. Y.

[wrcr21308-bib-0042] Kleinbaum, D. G. , and M. Klein (2005), Survival Analysis: A Self‐Learning Text, Springer, N. Y.

[wrcr21308-bib-0043] Klump, S. , R. Kipfer , O. A. Cirpka , C. F. Harvey , M. S. Brennwald , K. N. Ashfaque , A. B. M. Badruzzaman , S. J. Hug , and D. M. Imboden (2006), Groundwater dynamics and arsenic mobilization in Bangladesh assessed using noble gases and Tritium, Environ. Sci. Technol., 40(1), 243–250. 16433358 10.1021/es051284w

[wrcr21308-bib-0044] Lee, L. , and D. R. Helsel (2007), Statistical analysis of water‐quality data containing multiple detection limits II: S‐language software for nonparametric distribution modeling and hypothesis testing, Comput. Geosci., 33, 696–704.

[wrcr21308-bib-0045] McArthur, J. M. , et al. (2004), Natural organic matter in sedimentary basins and its relation to arsenic in anoxic groundwater: The example of West Bengal and its worldwide implications, Appl. Geochem., 19(8), 1255–1293.

[wrcr21308-bib-0046] McArthur, J. M. , P. Ravenscroft , D. M. Banerjee , J. Milsom , K. A. Hudson‐Edwards , S. Sengupta , C. Bristow , A. Sarkar , S. Tonkin , and R. Purohit (2008), How paleosols influence groundwater flow and arsenic pollution: A model from the Bengal Basin and its worldwide implication, Water Resour. Res., 44, W11411, doi:10.1029/2007WR006552.

[wrcr21308-bib-0047] McArthur, J. M. , D. M. Banerjee , S. Sengupta , P. Ravenscroft , S. Klump , A. Sarkar , B. Disch , and R. Kipfer (2010), Migration of As, and 3H/3He ages, in groundwater from West Bengal: Implications for monitoring, Water Res., 44, 4171–4185. 20542311 10.1016/j.watres.2010.05.010

[wrcr21308-bib-0048] McArthur, J. M. , P. Ravenscroft , and O. Sracek (2011a), Aquifer arsenic source, Nat. Geosci., 4(10), 655–656.

[wrcr21308-bib-0049] McArthur, J. M. , B. Nath , D. M. Banerjee , R. Purohit , and N. Grassineau (2011b), Palaeosol control on groundwater flow and pollutant distribution: The example of arsenic, Environ. Sci. Technol., 45(4), 1376–1383. 21268629 10.1021/es1032376

[wrcr21308-bib-0050] McCullagh, P. , and J. A. Nelder (1989), Generalized Linear Models, Chapman and Hall, London, U. K.

[wrcr21308-bib-0051] Meharg, A. A. , and M. M. Rahman (2003), Arsenic contamination of Bangladesh paddy field soils: Implications for rice contribution to arsenic consumption, Environ. Sci. Technol., 37(2), 229–234. 12564892 10.1021/es0259842

[wrcr21308-bib-0052] Michael, H. , and C. Voss (2009), Controls on groundwater flow in the Bengal Basin of India and Bangladesh: Regional modeling analysis, Hydrogeol. J., 17(7), 1561–1577.

[wrcr21308-bib-0053] MPO (1987), The groundwater resources and its availability for development, Tech. Rep. 5, Master Plan Organization (MPO), Ministry of Water Resources, GoB, Harza Engineering USA in association with Sir MacDonald and Partners, UK, Met Consultant, USA and EPC Ltd., Dhaka.

[wrcr21308-bib-0054] MPO (1991), Description of Groundwater Model Programs, National Water Plan Project Phase II, Final Report, Master Plan Organization (MPO), Ministry of Water Resources, GoB, Harza Engineering, USA, Sir M MacDonald & Partners, UK, Meta Consultants, USA and EPC Ltd., Dhaka.

[wrcr21308-bib-0055] Mukherjee, A. , M. von Brömssen , B. R. Scanlon , P. Bhattacharya , A. E. Fryar , M. A. Hasan , K. M. Ahmed , D. Chatterjee , G. Jacks , and O. Sracek (2008), Hydrogeochemical comparison and effects of overlapping redox zones on groundwater arsenic near the Western (Bhagirathi sub‐basin, India) and Eastern (Meghna sub‐basin, Bangladesh) margins of the Bengal Basin, J. Contam. Hydrol., 99(1–4), 31–48. 18164513 10.1016/j.jconhyd.2007.10.005

[wrcr21308-bib-0056] Nagelkerke, N. J. D. (1991), A note on a general definition of the coefficient of determination, Biometrika, 78(3), 691–692.

[wrcr21308-bib-0057] Neumann, R. B. , K. N. Ashfaque , A. B. M. Badruzzaman , M. Ashraf Ali , J. K. Shoemaker , and C. F. Harvey (2010), Anthropogenic influences on groundwater arsenic concentrations in Bangladesh, Nat. Geosci., 3(1), 46–52.

[wrcr21308-bib-0058] Neumann, R. B. , A. P. S. Vincent , L. C. Roberts , A. B. M. Badruzzaman , M. A. Ali , and C. F. Harvey (2011), Rice field geochemistry and hydrology: An explanation for why groundwater irrigated fields in Bangladesh are net sinks of arsenic from groundwater, Environ. Sci. Technol., 45(6), 2072–2078. 21332196 10.1021/es102635d PMC3995004

[wrcr21308-bib-0059] Nickson, R. , J. M. McArthur , W. G. Burgess , K. M. Ahmed , P. Ravenscroft , and M. Rahman (1998), Arsenic poisoning in Bangladesh groundwater, Nature, 395, 338. 9759723 10.1038/26387

[wrcr21308-bib-0060] Nickson, R. , J. M. McArthur , P. Ravenscroft , W. G. Burgess , and K. M. Ahmed (2000), Mechanism of arsenic release to groundwater, Bangladesh and West Bengal, Appl. Geochem., 15(4), 403–413.

[wrcr21308-bib-0061] Park, E. , and Y. Kim (2004), Analysis of longitudinal data in case‐control studies, Biometrika, 91, 321–330.

[wrcr21308-bib-0062] Pepe, M. S. , and G. Anderson (1994), A cautionary note on inference for marginal regression models with longitudinal data and general correlated response data, Commun. Stat., Part B, 23, 939–951.

[wrcr21308-bib-0063] Polizzotto, M. L. , B. D. Kocar , S. G. Benner , M. L. Sampson , and S. Fendorf (2008), Near‐surface wetland sediments as a source of arsenic release to ground water in Asia, Nature, 454, 505–509. 18650922 10.1038/nature07093

[wrcr21308-bib-0064] Qu, A. , and P. X.‐K. Song (2004), Assessing robustness of generalised estimating equations and quadratic inference functions, Biometrika, 91, 447–459.

[wrcr21308-bib-0065] R Development Core Team (2009), R: A Language and Environment for Statistical Computing, edited, R Found. for Stat. Comput., Vienna.

[wrcr21308-bib-0066] Radloff, K. A. , et al. (2011), Arsenic migration to deep groundwater in Bangladesh influenced by adsorption and water demand, Nat. Geosci., 4(11), 793–798. 22308168 10.1038/ngeo1283 PMC3269239

[wrcr21308-bib-0067] Ravenscroft, P. (2001), Distribution of groundwater arsenic in the Bangladesh related to geology, in Groundwater Arsenic Contamination in the Bengal Delta Plain of Bangladesh, Proceedings of KTH‐Dhaka University Seminar, KTH Spec. Publ., edited by Bhattacharya P., Jacks G., and Khan A. A., pp. 4–56, Royal Institute of Technology (KTH), Sweden.

[wrcr21308-bib-0068] Ravenscroft, P. (2003), Overview of the hydrogeology of Bangladesh, in Groundwater Resources and Development in Bangladesh—Background to the Arsenic Crisis, Agricultural Potential and the Environment, edited by Rahman A. A. and Ravenscroft P., pp. 43–86, Bangladesh Cent. for Adv. Stud., Univ. Press, Dhaka.

[wrcr21308-bib-0069] Ravenscroft, P. , W. G. Burgess , K. M. Ahmed , M. Burren , and J. Perrin (2005), Arsenic in groundwater of the Bengal Basin, Bangladesh: Distribution, field relations, and hydrogeological setting, Hydrogeol. J., 13, 727–751.

[wrcr21308-bib-0070] Ravenscroft, P. , H. Brammer , and K. S. Richards (2009), Arsenic Pollution: A Global Synthesis, 1st ed., 616 pp., John Wiley, Chichester, U. K.

[wrcr21308-bib-0071] Reich, E. S. (2011), Conflicting studies fuel arsenic debate, Nature, 478, 437–438. 22034667 10.1038/478437a

[wrcr21308-bib-0072] Romesburg, C. H. (2004), Cluster Analysis for Researchers, Lulu Press, Morrisville, N. C.

[wrcr21308-bib-0073] Ryberg, K. R. , and A. V. Vecchia (2006), Water‐quality trend analysis and sampling design for the Devils Lake Basin, North Dakota, January 1965 through September 2003, *Sci. Invest. Rep. 2006–5238*, 64 pp., U.S. Geol. Surv., Bismarck, North Dakota.

[wrcr21308-bib-0074] Shamsudduha, M. (2007), Spatial variability and prediction modeling of groundwater arsenic distributions in the shallowest alluvial aquifers in Bangladesh, J. Spatial Hydrol., 7(2), 33–46.

[wrcr21308-bib-0075] Shamsudduha, M. (2011), Groundwater Dynamics and Arsenic Mobilisation in Bangladesh: A National‐Scale Characterisation, 167 pp., Univ. Coll. London, London, U. K.

[wrcr21308-bib-0076] Shamsudduha, M. , R. E. Chandler , R. G. Taylor , and K. M. Ahmed (2009a), Recent trends in groundwater levels in a highly seasonal hydrological system: The Ganges‐Brahmaputra‐Meghna Delta, Hydrol. Earth Syst. Sci., 13(12), 2373–2385.

[wrcr21308-bib-0077] Shamsudduha, M. , L. J. Marzen , A. Uddin , M.‐K. Lee , and J. A. Saunders (2009b), Spatial relationship of groundwater arsenic distribution with regional topography and water‐table fluctuations in the shallow aquifers in Bangladesh, Environ. Geol., 57, 1521–1535.

[wrcr21308-bib-0078] Shamsudduha, M. , R. G. Taylor , K. M. Ahmed , and A. Zahid (2011), The impact of intensive groundwater abstraction on recharge to a shallow regional aquifer system: Evidence from Bangladesh, Hydrogeol. J., 19(4), 901–916.

[wrcr21308-bib-0079] Stute, M. , Y. Zheng , P. Schlosser , A. Horneman , R. K. Dhar , M. A. Hoque , A. A. Seddique , M. Shamsudduha , K. M. Ahmed , and A. van Geen (2007), Hydrological control of As concentrations in Bangladesh groundwater, Water Resour. Res., 43, W09417, doi:10.1029/2005WR004499.

[wrcr21308-bib-0080] Therneau, T. M. (2009), Survival Analysis Package Including Penalised Likelihood, version 2.35‐8, R Dev. Core Team CRAN. [Available at http://r‐forge.r‐project.org.]

[wrcr21308-bib-0081] Therneau, T. M. , P. M. Grambsch , and T. R. Fleming (1990), Martingale‐based residuals for survival models, Biometrika, 77(1), 147–160.

[wrcr21308-bib-0082] UNDP (1982), The hydrogeological conditions of Bangladesh, *Tech. Rep. DP/UN/BGD‐74‐009/1*, Dhaka.

[wrcr21308-bib-0083] van Geen, A. , Y. Zheng , M. Stute , and K. M. Ahmed (2003), Comment on “Arsenic Mobility and Groundwater Extraction in Bangladesh”, Science, 300(5619), 584. 12714725 10.1126/science.1081057

[wrcr21308-bib-0084] van Geen, A. , et al. (2006), Preliminary evidence of a link between surface soil properties and the arsenic content of shallow groundwater in Bangladesh, J. Geochem. Explor., 88, 157–161.

[wrcr21308-bib-0085] van Geen, A. , et al. (2008), Flushing history as a hydrogeological control on the regional distribution of arsenic in shallow groundwater of the Bengal basin, Environ. Sci. Technol., 42(7), 2283–2288. 18504954 10.1021/es702316k PMC3050603

[wrcr21308-bib-0086] van Geen, A. , et al. (2013), Retardation of arsenic transport through a Pleistocene aquifer, Nature, 501(7466), 204–207. 24025840 10.1038/nature12444 PMC3772538

[wrcr21308-bib-0087] WARPO (2000), National Water Management Plan (NWMP)—Draft development strategy report, Main Report, vol. 2, Dhaka.

[wrcr21308-bib-0088] Williams, P. N. , M. R. Islam , E. E. Adomako , A. Raab , S. A. Hossain , Y. G. Zhu , J. Feldmann , and A. A. Meharg (2006), Increase in rice grain arsenic for regions of Bangladesh irrigating paddies with elevated arsenic in groundwaters, Environ. Sci. Technol., 40(16), 4903–4908. 16955884 10.1021/es060222i

[wrcr21308-bib-0089] Winkel, L. H. E. , P. T. K. Trang , V. M. Lan , C. Stengel , M. Amini , N. T. Ha , P. H. Viet , and M. Berg (2011), Arsenic pollution of groundwater in Vietnam exacerbated by deep aquifer exploitation for more than a century, Proc. Natl. Acad. Sci. U. S. A., 108, 1246–1251. 21245347 10.1073/pnas.1011915108 PMC3029707

[wrcr21308-bib-0090] Yan, Z. , S. Bate , R. Chandler , V. Isham , and H. Wheater (2006), Changes in extreme wind speeds in NW Europe simulated by generalized linear models, Theor. Appl. Climatol., 83, 121–137.

[wrcr21308-bib-0091] Yu, W. H. , C. M. Harvey , and C. F. Harvey (2003), Arsenic in groundwater in Bangladesh: A geostatistical and epidemiological framework for evaluating health effects and potential remedies, Water Resour. Res., 39(6), 1146, doi:10.1029/2002WR001327.

[wrcr21308-bib-0092] Zheng, Y. , et al. (2005), Geochemical and hydrogeological contrasts between shallow and deeper aquifers in the two villages of Araihazar, Bangladesh: Implications for deeper aquifers as drinking water sources, Geochim. Cosmochim. Acta, 69(22), 5203–5218.

