# Supplementary material for: A generalized regression model of arsenic variations in the shallow groundwater of Bangladesh
Source: Water Resour Res. 2015 Jan 28;51(1):685–703. doi: 10.1002/2013WR014572 (PMC4964952; doi:10.1002/2013WR014572)
Supplement: Supplementary file 1 — Shamsudduha_etal_WRR_paper_SI_Readme_file_19Nov14 [file WRCR-51-685-s001.pdf]

Auxiliary Material for

A generalized regression model of Arsenic variations in the shallow groundwater of Bangladesh

Mohammad Shamsudduha

(Institute for Risk and Disaster Reduction, University College London, London WC1E 6BT, UK)

Richard G. Taylor

(Department of Geography, University College London, London WC1E 6BT, UK)

Richard E. Chandler

(Department of Statistical Science, University College London, London WC1E 6BT, UK)

Water Resources Research, 2014

Introduction

The auxiliary material contains a number of files. The main supplementary file contains
16 supporting figures (Figures S1 to S16) and 5 supporting tables (Tables S1 to S5) and
an appendix (Appendix A).

Two "comma separated value" files contain datasets for national- and regional-scale models.
Names of these data files are provided as follows:

Data file 1: Arsenic_calibration_n1643_dataset_19Nov14.csv
Data file 2: Arsenic_validation_n767_dataset_19Nov14.csv

There are 3 PDF files with "R" modeling codes:
These are the programming scripts for R Language (http://www.r-project.org).
These codes are used to fit the regression models for groundwater arsenic data and
to test statistical significance of each model covariate using an adjusted log-likelihood ratio (LR) test statistics.

R codes 1: R_codes_for_national_scale_GRM_19Nov14.pdf
R codes 2: R_codes_for_regional_scale_GRM_19Nov14.pdf
R codes 3: R_codes_for_adjusted_LR_test_19Nov14.pdf

One PDF file with detailed model results: national-scale, final generalized regression model

R outputs: Shamsudduha_etal_SI_Final_GRM_full_results_19Nov14.pdf
