# Supplementary material for: A generalized regression model of arsenic variations in the shallow groundwater of Bangladesh
Source: Water Resour Res. 2015 Jan 28;51(1):685–703. doi: 10.1002/2013WR014572 (PMC4964952; doi:10.1002/2013WR014572)
Supplement: Supplementary file 2 — R_codes_for_adjusted_LR_test_19Nov14 [file WRCR-51-685-s002.pdf]

```r
##########################################################################
# Function to campare restricted and extended models (class of lm, glm or
# survival) where observations fall within certain clusters and show inter-
# site depedence. This is a likelihood ratio test like anova() where cluster
# information is taken into account by default.
##########################################################################

# source("Bangladesh_As_adjusted_LR_test_GRM.r")

LRtest <- function(model0, model1, cluster) {

##########################################################################
# Compares restricted and extended models that have been fitted to the same
# set of clustered data using an "independence" loglikelihood. The comparison
# is done using an adjusted likelihood ratio test, as described in
# Chandler & Bate (Biometrika, 2007) but using the *vertical* scaling of the
# log-likelihood as in their equation (25), rather than the horizontal
# scaling that is studied in detail elsewhere in the paper (the two
# procedures are asymptotically equivalent, but vertical scaling is
# computationally cheaper). Note that with vertical scaling, the numerator
# of the scaling factor in equation (25) cancels with the denominator of the
# secondary adjustment in equation (20) - the whole thing can therefore be
# done at one stroke.
###########################################################################
# Arguments:
#
# model0     A model object corresponding to the restricted model
# model1     Ditto, extended model
# cluster    A vector of variables defining clusters for each case
#            in the database used for model (required if the model
#            objects are of class lm, glm, or survreg)
###########################################################################

 if (!identical(class(model0),class(model1))) {
  stop("model0 and model1 have different classes!")
 }
 if (any(class(model0) %in% c("lm","glm","survreg"))) {
#
# Check same response variable in each model (code lifted from
# anova.lm)
#
  responses <- as.character(lapply(list(model0,model1),
                                   function(x) deparse(x$terms[[2]])))
  if (!all(responses == responses[1])) {
  stop("Models have different responses")
  }
#
# Check that models are nested, and determine the constraints
# for the restricted one.
#
  theta0 <- coef(model0); theta1 <- coef(model1)
  if (!all(names(theta0) %in% names(theta1))) {
   stop("model0 is not nested within model1")
  }
  p0 <- length(theta0); p1 <- length(theta1)
#
#       For survreg objects, the scale parameter is also included
#       in the ML fit. Although this isn't included in all models ...
#       to find out whether or not to include it, nick some code
#       from getS3method("summary","survreg"). Also take the
#       opportunity to check that the survreg fit contains both
#       naive and robust covariance matrix estimates
#
  if (class(model1)[1] == "survreg") {
   if (is.null(model1$naive.var)) stop("no clusters defined in model1")
```

```r
   p.all <- nrow(model0$var)
   if (p.all > p0) {
    theta0 <- c(theta0,log(model0$scale))
    if (p.all - p0 == 1) {
     names(theta0) <- c(names(model0$coefficients),"Log(scale)")
    } else {
     names(theta0) <- c(names(model0$coefficients),names(model0$scale))
    }
    p0 <- p.all
   }
   p.all <- nrow(model1$var)
   if (p.all > p1) {
    theta1 <- c(theta1,log(model1$scale))
    if (p.all - p1 == 1) {
     names(theta1) <- c(names(model1$coefficients),"Log(scale)")
    } else {
     names(theta1) <- c(names(model1$coefficients),names(model1$scale))
    }
    p1 <- p.all
   }
  }
  kept.pars <- match(names(theta0),names(theta1))
  theta.tmp <- round(theta1 - theta1)      # To copy the coefficient names
  theta.tmp[kept.pars] <- theta0
  theta0 <- theta.tmp
  zero.pos <- (1:p1)[-kept.pars]

################################################################################
# Covariance matrix calculations, and log-likelihoods. For  linear and
# generalised linear models the latter are derived from the deviances /
# residual sums of squares, converted back to the log-likelihood scale,
# because it isn't clear that the logLik function gives the right thing.
# Actually, in the case of an unknown dispersion parameter we do *not*
# use full ML fits of both models - rather, we take the moment estimate from
# the larger model as fixed. This is OK, because the estimate will differ
# from the *true* dispersion parameter by an amount that is O(k^-0.5) in
# probability and the computed (naive) likelihood ratio statistic is
# calculated as (D0-D1) / \hat{phi} = [(D0-D1)/phi] * [1 + O(k^-0.5)]
# which is asymptotically equivalent to the chi-squared statistic you'd get
# if phi were known. The reason for proceeding this way is to ensure
# compatibility with (e.g.) quasi-Wald statistics, which just use dispersion
# estimates from the extended model. If you *don't* do this, then the ML
# estimate of the dispersion parameter in the restricted model can `hide'
# much of the lack of fit and reduce the significance of extra terms
# (or, at least, indicate that they're not necessary because the extra
# variation can be explained via increased dispersion). That seems a bit
# heuristic - need to understand this a bit better! NB can't use the normal
# F ratio to cope with the unknown dispersion, because the residual deviance
# doesn't have the same chi-squared distribution as in the independence case.
###############################################################################

  n <- p1 + model1$df.residual
  if (class(model1)[1] == "lm") {
   summ1 <- lm.clus.sum(model1,cluster=cluster)
   sigsq <- summ1$sigma^2
   logL1 <- -model1$df.residual
   RSS0 <- (n-p0) * summary(model0)$sigma^2
   logL0 <- -RSS0/sigsq
   R <- summ1$cov.scaled; N <- summ1$cov.unscaled
  } else if (class(model1)[1] == "glm") {
   if (!identical(model0$family[1:2],model1$family[1:2])) {
    stop("model0 and model1 have different 'family' attributes")
   }
   summ0 <- summary(model0)
   summ1 <- glm.clus.sum(model1,cluster=cluster)
```

```
    dispersion <- summ1$dispersion
    logL0 <- -summ0$deviance / (2*dispersion)
    logL1 <- -summ1$deviance / (2*dispersion)
    R <- summ1$cov.scaled; N <- summ1$cov.unscaled
   }  else if (class(model1)[1] == "survreg") {
    logL0 <- model0$loglik[2]
    logL1 <- model1$loglik[2]
    R <- model1$var; N <- model1$naive.var
   }
  }

#
# OK: here's the scaling factor.
#
 thetadiff <- theta1 - theta0; psidiff <- theta1[zero.pos]
 H <- -solve(N); H.adj <- -solve(R)
 R.psi.inv <- -solve(R[zero.pos,zero.pos])
 scalfac <- (t(psidiff) %*% R.psi.inv %*% psidiff) /
                  (t(thetadiff) %*% H %*% thetadiff)
 df <- p1-p0
 LR.naive <- 2*(logL1-logL0)
 pval.naive <- pchisq(LR.naive,df=df,lower.tail=FALSE)
 LR.adj <- LR.naive*scalfac
 pval.adj <- pchisq(LR.adj,df=df,lower.tail=FALSE)
 data.frame(Lambda=c(LR.naive,LR.adj),
            P.value=c(pval.naive,pval.adj),
            row.names=c("Naive","Adjusted"))
}

# end of the script written by Richard Chandler, Professor of Statistics at
# the Department of Statistical Science at University College London.
# E-mail: r.chandler@ucl.ac.uk
```
