# Supplementary material for: A generalized regression model of arsenic variations in the shallow groundwater of Bangladesh
Source: Water Resour Res. 2015 Jan 28;51(1):685–703. doi: 10.1002/2013WR014572 (PMC4964952; doi:10.1002/2013WR014572)
Supplement: Supplementary file 3 — R_codes_for_national_scale_GRM_19Nov14 [file WRCR-51-685-s003.pdf]

```r
################################################################################
# Generalized regression model (GRM) for describing the nation-scale variations in As
# concentrations in Bangladesh. Survival regression techniques have been applied here.
# Models are constructed in R statistical language platform (http://www.r-project.org/).
################################################################################

# First load the required libraries (R packages):

library(survival); library(rms); library(gstat); library(NADA); library(sp)

# source("Bangladesh_As_national_GRM_15Aug14.r")                 # for fitting survreg model
# source("Bangladesh_As_adjusted_LR_test_GRM.r")                 # for comparing models: LR test

options(contrasts=c("contr.sum", "contr.poly"))
options(show.signif.stars = TRUE)

# Load calibration dataset to fit the national-scale, comprehensive (full) GRM:

model.data <- read.csv("Arsenic_calibration_n1643_dataset_15Aug14.csv", header=T, sep=",")
model.data$GeolAbb <- as.factor(model.data$GeolAbb)

dd <- datadist(model.data)
options(datadist="dd")

# names(model.data)                                              # to print the data headers


# Run the regression model with a Weibull distribution. This is the national-scale GRM (model0):

model0 <- survreg(Surv(Arsenic, Censor, type = "left") ~ factor(GeolAbb) + USCunit + HydCond +
                  SyPerc + Darcy + WellDepth + WetGWT + GWLtrend + MeanGWF + RechPGI +
                  Rchange + LongLeg1 + LatLeg1 + LongLeg2 + LatLeg2 + LongLeg1:LatLeg1 +
                  Elevation + DCos + DSin + IrrigTrends + factor(GeolAbb):WellDepth +
                  factor(GeolAbb):USCunit + factor(GeolAbb):RechPGI + cluster(ClusterID),
                  data=model.data, dist="weibull")

model0$coefficients <- round(model0$coefficients, 4)
print("model0"); print(summary(model0))                          # to print model results


# Alternatively, one can run survival regression model using psm() function from the RMS package
# The advantage of using the psm() function is that the resulting model provides additional
# information such as R2 value and model effect can easily be visualized using plot() function.

mod.psm <- psm(Surv(Arsenic, Censor, type = "left") ~ GeolAbb + USCunit + HydCond + SyPerc +
               Darcy + WellDepth + WetGWT + GWLtrend + MeanGWF + RechPGI + Rchange +
               LongLeg1 + LatLeg1 + LongLeg2 + LatLeg2 + LongLeg1:LatLeg1 + Elevation + DCos +
               DSin + IrrigTrends + GeolAbb:WellDepth + GeolAbb:USCunit + GeolAbb:RechPGI +
               cluster(ClusterID), data=model.data, dist="weibull")

# In order to visualize model effects for each covariate it is necesary to remove the cluster
# from the model fitting.

mod.red <- psm(Surv(Arsenic, Censor, type = "left") ~ GeolAbb + USCunit + HydCond + SyPerc +
               Darcy + WellDepth + WetGWT + GWLtrend + MeanGWF + RechPGI + Rchange +
               LongLeg1 + LatLeg1 + LongLeg2 + LatLeg2 + LongLeg1:LatLeg1 + Elevation + DCos +
               DSin + IrrigTrends + GeolAbb:WellDepth + GeolAbb:USCunit + GeolAbb:RechPGI,
               data=model.data, dist="weibull")

var.names <- c("HydCond", "Rchange", "IrrigTrends")
plot(Predict(mod.red, name=var.names, ref.zero=F), ylab="log(As)", lwd=2,
             aspect=1, index.cond=list(c(3,2,1)))

# Now extract deviance residuals from the calibration model for diagnostics:
```

```r
cur.data <- cbind(model.data, RegRes=residuals(model0, type="deviance"))


# First, check whether the assumption for choosing the Weibull distribution is correct.
# A straight line indicates that the chosen distribution is appropriate for the As dataset.

par(mfrow=c(2,2), mgp=c(2,0.5,0), tck=-0.03, cex.axis=0.9, cex.main=1, cex.lab=1)

ntimes <- model.data$Arsenic * exp(-model0$linear.predictors)
plot(survfit(Surv(ntimes, Censor, type="left") ~ 1, data=model.data), fun="cloglog", cex=0.5,
             axes=F, lty=2:3, xlab=(expression(log(tau))), ylab=(expression(log(-log(1-F(tau))))))
axis(2, at=-4:2, lab=seq(-4,2))
axis(1, at=c(0.01, 0.1, 1, 10, 100), lab=c(0.01, 0.1, 1, 10, 100))
main <- title(paste("Calibration: Weibull model assumption"))
box()


# Now plot deviance residuals on a map to check spatial distribution of the model residuals.
# If the model fitting is sensible then the residuals should fall within a range of -2 to +2.

model.res <- data.frame(cbind(cur.data$Long, cur.data$Lat, cur.data$RegRes))   # model residuals

names(model.res) <- c("x", "y", "Res")
coordinates(model.res) = ~x + y

spplot(model.res, "Res", key.space=list(x=0.7,y=0.96,corner=c(0,1)), scales=list(draw=T),
       col.regions=rev(rainbow(6)), cuts=c(-4,-2,-1,1,2,4), xlim=c(87.5,93), ylim=c(20.5,27),
       main=paste("Calibration GRM residuals", sep=""))


# Now construct a variogram of the GLM residuals to see any spatial depenedency of residuals:

par(mfrow=c(2,2))

res.vgm <- variogram(Res ~ 1, data=model.res)
var.dist <- as.numeric(res.vgm$dist)
var.gamma <- as.numeric(res.vgm$gamma)

plot(var.gamma ~ var.dist, pch=21, cex=2, cex.lab=1.2, col="blue", ylim=c(0,2),
                 xlab="Distance (degrees)", ylab="Semivariance", main="Variogram of GRM residuals")
abline(h=var(cur.data$RegRes), lty=2, lwd=3, col="red")



# Check the reproductive capacity of the fitted GRM with validation dataset:

valid.data <- read.csv("Arsenic_validation_n767_dataset_15Aug14.csv", header=T, sep=",")

# Fit the model without ClusterID now before validation data can be used to test the model.

grm.model <- survreg(Surv(Arsenic, Censor, type = "left") ~ factor(GeolAbb) + USCunit + HydCond +
                     SyPerc + Darcy + WellDepth + WetGWT + GWLtrend + MeanGWF + RechPGI +
                     Rchange + LongLeg1 + LatLeg1 + LongLeg2 + LatLeg2 + LongLeg1:LatLeg1 +
                     Elevation + DCos + DSin + IrrigTrends + factor(GeolAbb):WellDepth +
                     factor(GeolAbb):USCunit + factor(GeolAbb):RechPGI,
                     data=model.data, dist="weibull")

alpha <- grm.model$scale                                                  # extract the scale value

eta.pred <- predict(grm.model, newdata=valid.data, type="linear", se.fit=F)
eta.reg <- survreg(Surv(Arsenic, Censor, type = "left") ~ eta.pred - 1, init=1, scale=alpha,
                        data=valid.data, dist="weibull", control=list(iter=0))

# Check the Weibull distribution assumption for the validation data using the following code:
```

```r
par(mfrow=c(2,2), mgp=c(2,0.5,0), tck=-0.03, cex.axis=0.9, cex.main=1, cex.lab=1)

ntimes <- valid.data$Arsenic * exp(-eta.reg$linear.predictors)
plot(survfit(Surv(ntimes, Censor, type="left")~1, data=valid.data), fun="cloglog", cex=0.5,
     axes=F, lty=2:3, xlab=(expression(log(tau))), ylab=(expression(log(-log(1-F(tau))))))
axis(2, at=-4:2, lab=seq(-4,2))
axis(1, at=c(0.01, 0.1, 1, 10, 100), lab=c(0.01, 0.1, 1, 10, 100))
main <- title(paste("Validation: Weibull model assumption"))
box()


# Now plot deviance residuals on a map to check spatial distribution of the model residuals.
# If the model fitting is sensible then the residuals should fall within a range of -2 to +2.

cur.data1 <- cbind(valid.data, ValidRes=residuals(eta.reg, type="deviance"))   # model residuals
model.res1 <- data.frame(cbind(cur.data1$Long, cur.data1$Lat, cur.data1$ValidRes))
names(model.res1) <- c("x", "y", "Res")
coordinates(model.res1) = ~x + y

spplot(model.res1, "Res", key.space=list(x=0.7,y=0.96,corner=c(0,1)), scales=list(draw=T),
       col.regions=rev(rainbow(6)), cuts=c(-4,-2,-1,1,2,4),  xlim=c(87.5,93), ylim=c(20.5,27),
       main=paste("Validation GRM residuals", sep=""))


# At this stage GRM has been successfully fitted, model performance and diagnostics have been
# done; fitted GRM has been validated using a sebset of As dataset.
# Now systematically drop model terms and test the significance of the terms using the adjusted
# log-likelihood ratio test (LR test). Start by dropping model terms from the national-scale GRM.
# Data groups are described in the paper: (1) Geology and hydrogeological factors;
# (2) hydrodynamics and groundwater recharge factors; (3) Geographical, altitudinal and
# seasonal factors; (4) Groundwater-fed irrigation; and (5) Geological interactions.
# First load the function [LRtest()] first. Interpretation: a low adjusted p value suggests
# that deletion of the term badly affect the model fitting and, therefore, the term represented
# by the covariate is considered to be a significant covariate for arsenic model.

# source("Bangladesh_As_adjusted_LR_test_GRM.r")                  # for comparing models: LR test

# Surface geology and hydrogeological factors:

model1 <- update(model0, ~ . - (factor(GeolAbb) + factor(GeolAbb):WellDepth +
                                factor(GeolAbb):RechPGI + factor(GeolAbb):USCunit))
print("model1"); print(LRtest(model1, model0))

model2 <- update(model0, ~ . - (factor(GeolAbb) + USCunit + factor(GeolAbb):WellDepth +
                                factor(GeolAbb):RechPGI + factor(GeolAbb):USCunit))
print("model2"); print(LRtest(model2, model0))

model3 <- update(model0, ~ . - (factor(GeolAbb):WellDepth + factor(GeolAbb):RechPGI +
                                factor(GeolAbb):USCunit))
print("model3"); print(LRtest(model3, model0))

model3a <- update(model0, ~ . - (factor(GeolAbb):USCunit + USCunit))
print("model3a"); print(LRtest(model3a, model0))

model3b <- update(model0, ~ . - (factor(GeolAbb):WellDepth + WellDepth))
print("model3b"); print(LRtest(model3b, model0))

model4 <- update(model0, ~ . - (factor(GeolAbb) + USCunit + HydCond + SyPerc + Darcy +
                                WellDepth + factor(GeolAbb):WellDepth + factor(GeolAbb):RechPGI +
                                factor(GeolAbb):USCunit))
print("model4"); print(LRtest(model4, model0))

model5 <- update(model0, ~ . - (HydCond + SyPerc + Darcy))
print("model5"); print(LRtest(model5, model0))
```

```
model5a <- update(model0, ~ . - (HydCond))
print("model5a"); print(LRtest(model5a, model0))

model5b <- update(model0, ~ . - (SyPerc))
print("model5b"); print(LRtest(model5b, model0))

model5c <- update(model0, ~ . - (Darcy))
print("model5c"); print(LRtest(model5c, model0))

model6 <- update(model0, ~ . - (WetGWT + Elevation))
print("model6"); print(LRtest(model6, model0))

model6a <- update(model0, ~ . - (WetGWT))
print("model6a"); print(LRtest(model6a, model0))

# Hydrodynamic and groundwater recharge processes:

model7 <- update(model0, ~ . - (RechPGI + Rchange + GWLtrend + factor(GeolAbb):RechPGI))
print("model7"); print(LRtest(model7, model0))

model8 <- update(model0, ~ . - (RechPGI + factor(GeolAbb):RechPGI))
print("model8"); print(LRtest(model8, model0))

model9 <- update(model0, ~ . - (WetGWT + GWLtrend + MeanGWF + RechPGI + Rchange +
                                factor(GeolAbb):RechPGI))
print("model9"); print(LRtest(model9, model0))

model9a <- update(model0, ~ . - (Rchange))
print("model9a"); print(LRtest(model9a, model0))

model9b <- update(model0, ~ . - (GWLtrend))
print("model9b"); print(LRtest(model9b, model0))

# Geographical, altitudinal and seasonal factors:

model10 <- update(model0, ~ . - (LongLeg1 + LatLeg1 + LongLeg2 + LatLeg2 + LongLeg1 + LatLeg1 +
                                 LongLeg1:LatLeg1 + DCos + DSin + Elevation))
print("model10"); print(LRtest(model10, model0))

model10a <- update(model0, ~ . - (Elevation))
print("model10a"); print(LRtest(model10a, model0))

model10b <- update(model0, ~ . - (DCos + DSin))
print("model10b"); print(LRtest(model10b, model0))

# Groundwater abstraction (irrigation trends):

model11 <- update(model0, ~ . - (IrrigTrends))
print("model11"); print(LRtest(model11, model0))

model12 <- update(model0, ~ . - (IrrigTrends + Rchange))
print("model12"); print(LRtest(model12, model0))

model12a <- update(model0, ~ . - (IrrigTrends + Rchange + RechPGI + factor(GeolAbb):RechPGI))
print("model12a"); print(LRtest(model12a, model0))


# LR test shows that not all the covariates considering in the comprehensive model are not
# statistically significant and therefore can be removed from the model. Consequently,
# the final, national-scale model forms that can be expressed by the following formula:

final.grm <- survreg(Surv(Arsenic, Censor, type = "left") ~ factor(GeolAbb) + USCunit + HydCond +
                     WellDepth + GWLtrend + RechPGI + Rchange + LongLeg1 + LatLeg1 + LongLeg2 +
                     LatLeg2 + LongLeg1:LatLeg1 + IrrigTrends + factor(GeolAbb):WellDepth +
                     factor(GeolAbb):USCunit + factor(GeolAbb):RechPGI + cluster(ClusterID),
```

```r
                     data=model.data, dist="weibull")
final.grm$coefficients <- round(final.grm$coefficients, 4)
print("final.grm"); print(summary(final.grm))


# Alternatively, one can run survival regression model using psm() function from the RMS package
# The advantage of using the psm() function is that the resulting model provides additional
# information such as R2 value and model effect can easily be visualized using plot() function.

final.mod <- psm(Surv(Arsenic, Censor, type = "left") ~ GeolAbb + USCunit + HydCond +
                 WellDepth + GWLtrend + RechPGI + Rchange + LongLeg1 + LatLeg1 +
                 LongLeg2 + LatLeg2 + IrrigTrends + GeolAbb:WellDepth +
                 GeolAbb:USCunit + GeolAbb:RechPGI, data=model.data, dist="weibull")

var.names <- c("HydCond", "Rchange", "IrrigTrends")

plot(Predict(final.mod, name=var.names, ref.zero=F), ylab="ln(As)", asp=1, lwd=2.5, layout=c(3,1),
             par.settings=my.settings, index.cond=list(c(1,3,2)))

grm.save <- paste("Final_national_GRM_model_effects_plot_15Aug14", ".pdf", sep="")
dev.dims <- dev.size(units=c("in"))
# dev.copy(pdf, grm.save, width=dev.dims[1], height=dev.dims[2])
# dev.off()

# Derive LR-test statistics using the final GRM and by dropping individual covariates and their
# statistical interactions terms.

model5a <- update(final.grm, ~ . - (HydCond))
print("model5a"); print(LRtest(model5a, final.grm))

model9a <- update(final.grm, ~ . - (Rchange))
print("model9a"); print(LRtest(model9a, final.grm))

model9b <- update(final.grm, ~ . - (GWLtrend))
print("model9b"); print(LRtest(model9b, final.grm))

model11 <- update(final.grm, ~ . - (IrrigTrends))
print("model11"); print(LRtest(model11, final.grm))

model3a <- update(final.grm, ~ . - (factor(GeolAbb):USCunit + USCunit))
print("model3a"); print(LRtest(model3a, final.grm))

model3b <- update(final.grm, ~ . - (factor(GeolAbb):WellDepth + WellDepth))
print("model3b"); print(LRtest(model3b, final.grm))

model8 <- update(final.grm, ~ . - (RechPGI + factor(GeolAbb):RechPGI))
print("model8"); print(LRtest(model8, final.grm))

# Model outputs (e.g., residuals) can be saved to a comma-separated value file as follows:

# outflnm <- paste("Arsenic_national_GRM_model_residuals_15Aug12", ".csv", sep="")
# write.table(cur.data, outflnm, row.names=F, col.names=T, sep=",")

# End of script

# R codes written by M Shamsudduha, last modified on 15 August 2014 at UCL Institute for Risk and
# Disaster Reduction. Contact e-mail: m.shamsudduha@ucl.ac.uk
```
