# Supplementary material for: A generalized regression model of arsenic variations in the shallow groundwater of Bangladesh
Source: Water Resour Res. 2015 Jan 28;51(1):685–703. doi: 10.1002/2013WR014572 (PMC4964952; doi:10.1002/2013WR014572)
Supplement: Supplementary file 5 — Shamsudduha_etal_SI_Final_GRM_full_results_19Nov14 [file WRCR-51-685-s005.pdf]

Supplementary Information: Model results for the final, national-scale GRM

Model outputs from R programing language:

Parametric Survival Model: Weibull Distribution

```
survreg(formula = Surv(Arsenic, Censor, type = "left") ~ factor(GeolAbb) +
        USCunit + HydCond + WellDepth + GWLtrend + RechPGI + Rchange +
        LongLeg1 + LatLeg1 + LongLeg2 + LatLeg2 + LongLeg1:LatLeg1 +
        IrrigTrends + factor(GeolAbb):WellDepth + factor(GeolAbb):USCunit +
        factor(GeolAbb):RechPGI + cluster(ClusterID), data = model.data,
        dist = "weibull")
```

| | Value | Std. Err | (Naive SE) | z | p |
|---|---|---|---|---|---|
| (Intercept) | 4.6475 | 0.48114 | 0.528912 | 9.6594 | 4.48e-22 |
| factor(GeolAbb)1 | -2.0228 | 2.47101 | 1.893165 | -0.8186 | 4.13e-01 |
| factor(GeolAbb)2 | -3.6682 | 1.29453 | 0.876850 | -2.8336 | 4.60e-03 |
| factor(GeolAbb)3 | 3.0609 | 0.86422 | 0.811725 | 3.5418 | 3.97e-04 |
| factor(GeolAbb)4 | -0.8736 | 1.30705 | 1.220783 | -0.6684 | 5.04e-01 |
| factor(GeolAbb)5 | 1.6829 | 0.99754 | 0.782695 | 1.6870 | 9.16e-02 |
| factor(GeolAbb)6 | -0.0530 | 1.45670 | 1.104601 | -0.0364 | 9.71e-01 |
| factor(GeolAbb)7 | -1.7956 | 1.26031 | 1.273628 | -1.4247 | 1.54e-01 |
| factor(GeolAbb)8 | 0.4504 | 0.64950 | 1.180497 | 0.6935 | 4.88e-01 |
| factor(GeolAbb)9 | -2.7515 | 2.23123 | 2.223838 | -1.2332 | 2.18e-01 |
| factor(GeolAbb)10 | 1.1280 | 1.25356 | 1.839817 | 0.8998 | 3.68e-01 |
| factor(GeolAbb)11 | 0.9383 | 0.77063 | 0.860189 | 1.2176 | 2.23e-01 |
| factor(GeolAbb)12 | -2.5077 | 1.02506 | 0.825883 | -2.4464 | 1.44e-02 |
| factor(GeolAbb)13 | 0.4948 | 1.21544 | 0.950879 | 0.4071 | 6.84e-01 |
| factor(GeolAbb)14 | 2.5548 | 1.94457 | 1.565542 | 1.3138 | 1.89e-01 |
| USCunit | -0.0233 | 0.01744 | 0.019820 | -1.3357 | 1.82e-01 |
| HydCond | -0.0256 | 0.00960 | 0.007216 | -2.6677 | 7.64e-03 |
| WellDepth | -0.0124 | 0.00916 | 0.009720 | -1.3532 | 1.76e-01 |
| GWLtrend | 0.0339 | 0.01409 | 0.011848 | 2.4064 | 1.61e-02 |
| RechPGI | -0.0006 | 0.00403 | 0.004033 | -0.1489 | 8.82e-01 |
| Rchange | -0.0043 | 0.00123 | 0.000957 | -3.5086 | 4.50e-04 |
| LongLeg1 | -0.6278 | 0.34899 | 0.367816 | -1.7989 | 7.20e-02 |
| LatLeg1 | -1.2590 | 0.49703 | 0.453192 | -2.5331 | 1.13e-02 |
| LongLeg2 | -0.8508 | 0.29375 | 0.255948 | -2.8963 | 3.78e-03 |
| LatLeg2 | -0.6961 | 0.70548 | 0.561110 | -0.9867 | 3.24e-01 |
| LongLeg1:LatLeg1 | 0.6615 | 0.58344 | 0.614508 | 1.1338 | 2.57e-01 |
| IrrigTrends | -0.0564 | 0.03006 | 0.022457 | -1.8763 | 6.06e-02 |
| factor(GeolAbb)1:WellDepth | 0.0460 | 0.06266 | 0.042613 | 0.7341 | 4.63e-01 |
| factor(GeolAbb)2:WellDepth | 0.1043 | 0.02487 | 0.018876 | 4.1939 | 2.74e-05 |
| factor(GeolAbb)3:WellDepth | -0.0468 | 0.01633 | 0.016045 | -2.8661 | 4.16e-03 |
| factor(GeolAbb)4:WellDepth | 0.0395 | 0.03939 | 0.038301 | 1.0029 | 3.16e-01 |
| factor(GeolAbb)5:WellDepth | 0.0217 | 0.01598 | 0.014806 | 1.3583 | 1.74e-01 |
| factor(GeolAbb)6:WellDepth | 0.0016 | 0.04745 | 0.027597 | 0.0337 | 9.73e-01 |
| factor(GeolAbb)7:WellDepth | -0.0532 | 0.02204 | 0.027088 | -2.4140 | 1.58e-02 |
| factor(GeolAbb)8:WellDepth | -0.0130 | 0.01594 | 0.025617 | -0.8154 | 4.15e-01 |
| factor(GeolAbb)9:WellDepth | -0.0629 | 0.07318 | 0.066378 | -0.8596 | 3.90e-01 |
| factor(GeolAbb)10:WellDepth | 0.0084 | 0.02036 | 0.045213 | 0.4126 | 6.80e-01 |
| factor(GeolAbb)11:WellDepth | 0.0155 | 0.01725 | 0.016536 | 0.8985 | 3.69e-01 |
| factor(GeolAbb)12:WellDepth | 0.0384 | 0.01387 | 0.017328 | 2.7678 | 5.64e-03 |
| factor(GeolAbb)13:WellDepth | -0.0081 | 0.02313 | 0.019875 | -0.3502 | 7.26e-01 |
| factor(GeolAbb)14:WellDepth | 0.0000 | 0.03675 | 0.034355 | 0.0000 | 1.00e+00 |
| factor(GeolAbb)1:USCunit | -0.0621 | 0.03939 | 0.047992 | -1.5767 | 1.15e-01 |
| factor(GeolAbb)2:USCunit | 0.0538 | 0.02567 | 0.030444 | 2.0960 | 3.61e-02 |
| factor(GeolAbb)3:USCunit | -0.0869 | 0.03645 | 0.034630 | -2.3840 | 1.71e-02 |
| factor(GeolAbb)4:USCunit | 0.0774 | 0.06208 | 0.046120 | 1.2468 | 2.12e-01 |
| factor(GeolAbb)5:USCunit | -0.1297 | 0.04309 | 0.029828 | -3.0098 | 2.61e-03 |
| factor(GeolAbb)6:USCunit | 0.0682 | 0.07711 | 0.069355 | 0.8845 | 3.76e-01 |
| factor(GeolAbb)7:USCunit | 0.0962 | 0.04611 | 0.052974 | 2.0863 | 3.69e-02 |

```
factor(GeolAbb)8:USCunit     0.0355  0.03767   0.067396  0.9425 3.46e-01
factor(GeolAbb)9:USCunit     0.1564  0.10364   0.095686  1.5091 1.31e-01
factor(GeolAbb)10:USCunit   -0.0080  0.05288   0.107360 -0.1513 8.80e-01
factor(GeolAbb)11:USCunit   -0.0378  0.03319   0.032996 -1.1388 2.55e-01
factor(GeolAbb)12:USCunit   -0.0231  0.03696   0.030869 -0.6249 5.32e-01
factor(GeolAbb)13:USCunit   -0.0069  0.03212   0.031252 -0.2148 8.30e-01
factor(GeolAbb)14:USCunit    0.0035  0.04334   0.058935  0.0807 9.36e-01

factor(GeolAbb)1:RechPGI     0.0033  0.00986   0.007655  0.3346 7.38e-01
factor(GeolAbb)2:RechPGI     0.0030  0.00429   0.004299  0.6990 4.85e-01
factor(GeolAbb)3:RechPGI    -0.0018  0.00401   0.004048 -0.4483 6.54e-01
factor(GeolAbb)4:RechPGI    -0.0010  0.00432   0.004516 -0.2316 8.17e-01
factor(GeolAbb)5:RechPGI     0.0002  0.00436   0.004121  0.0459 9.63e-01
factor(GeolAbb)6:RechPGI    -0.0289  0.01449   0.010593 -1.9951 4.60e-02
factor(GeolAbb)7:RechPGI    -0.0005  0.01130   0.012255 -0.0443 9.65e-01
factor(GeolAbb)8:RechPGI     0.0068  0.00958   0.012903  0.7099 4.78e-01
factor(GeolAbb)9:RechPGI     0.0246  0.04298   0.042860  0.5723 5.67e-01
factor(GeolAbb)10:RechPGI   -0.0034  0.00924   0.012852 -0.3681 7.13e-01
factor(GeolAbb)11:RechPGI    0.0026  0.00432   0.004633  0.6015 5.48e-01
factor(GeolAbb)12:RechPGI    0.0289  0.01194   0.008085  2.4199 1.55e-02
factor(GeolAbb)13:RechPGI   -0.0014  0.00584   0.004621 -0.2396 8.11e-01
factor(GeolAbb)14:RechPGI   -0.0215  0.00907   0.006859 -2.3717 1.77e-02
Log(scale)                   0.6515  0.05721   0.023417 11.3885 4.77e-30

Scale= 1.92

Weibull distribution
Loglik(model)= -6219.9   Loglik(intercept only)= -6780.2
        Chisq= 1120.45 on 68 degrees of freedom, p= 0
(Loglikelihood assumes independent observations)
Number of Newton-Raphson Iterations: 6
n= 1643
```

Abbreviation Key:

USCunit=thickness of surficial silt and clay (TSSC),
HydCond=hydraulic conductivity,
WellDepth=surveyed tubewell depth,
GWLtrend= trends in mean groundwater levels,
RechPGI=mean recharge for pre-developed groundwater-fed irrigation period,
Rchange=net changes in groundwater recharge,
Long=tubewell location longitude,
Lat=tubewell location latitude, and
IrrigTrends=groundwater-fed irrigation trends.
Value denotes model coefficient;
Std. Err and S.E. denote standard error of coefficients, and
(:) denotes statistical interaction term.
