# Supplementary material for: A generalized regression model of arsenic variations in the shallow groundwater of Bangladesh
Source: Water Resour Res. 2015 Jan 28;51(1):685–703. doi: 10.1002/2013WR014572 (PMC4964952; doi:10.1002/2013WR014572)
Supplement: Supplementary file 6 — Shamsudduha_etal_WRR_supplementary_information_15Dec2014 [file WRCR-51-685-s006.docx]

### Supplementary Information

**A generalized regression model of Arsenic variations in the shallow groundwater of Bangladesh**

**Mohammad Shamsudduha^1^, Richard G. Taylor^2^ and Richard E. Chandler^3^**

^1^ Institute for Risk and Disaster Reduction, University College London, London WC1E 6BT, UK

^2^ Department of Geography, University College London, London WC1E 6BT, UK

^3^ Department of Statistical Science, University College London, London WC1E 6BT, UK

Corresponding author: M. Shamsudduha (e-mail: m.shamsudduha@ucl.ac.uk)

### Supplementary Figures:


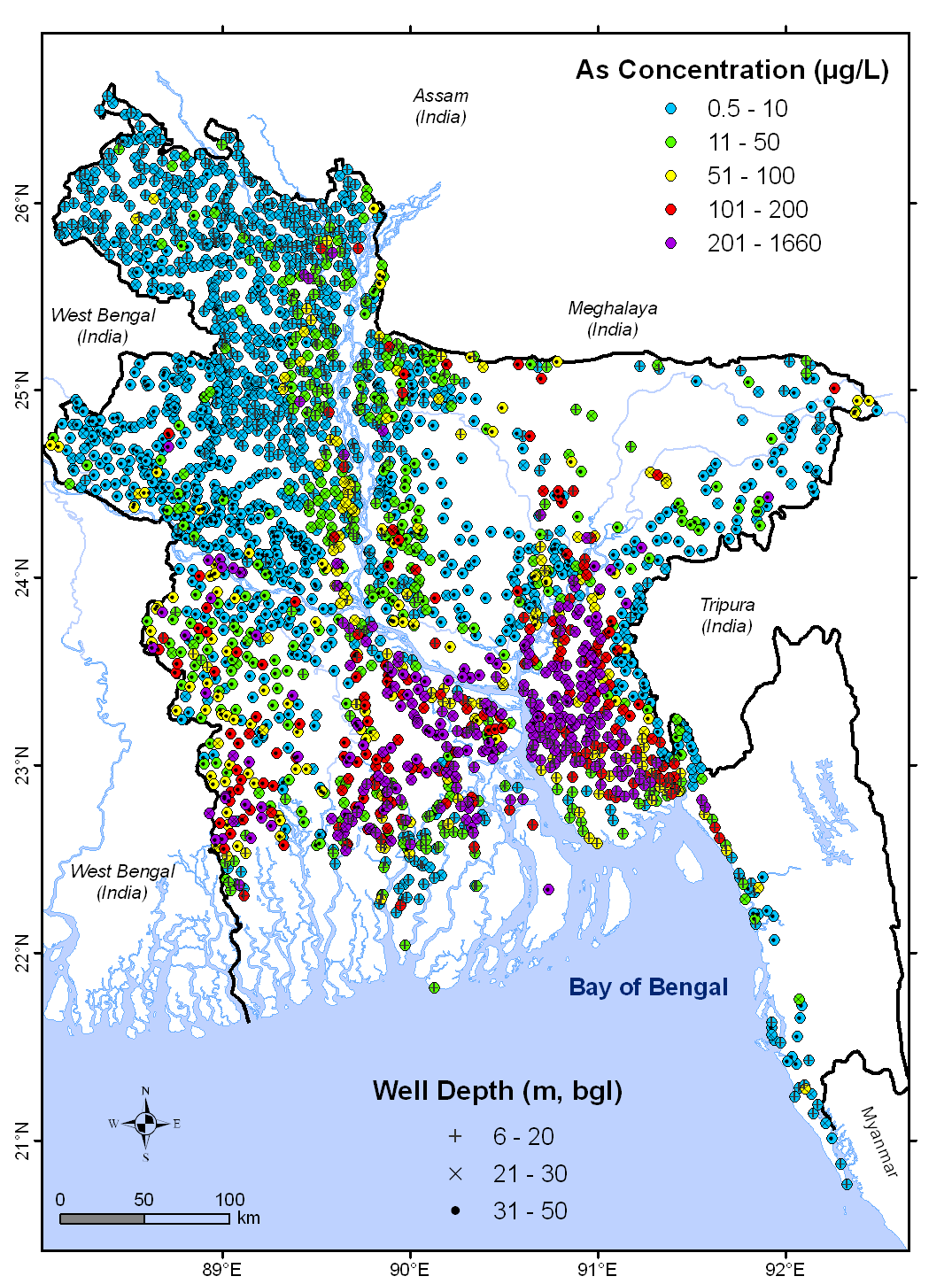


**Figure S1.** Spatial distribution of one-off surveyed As concentrations in shallow groundwater (sampling depth ≤50 m bgl) in Bangladesh [[*BGS and DPHE*, 2001](#_ENREF_4)]. Highest As concentrations are observed in the southeastern and south-central regions in the country where tubewell depths are mostly very shallow (<30 m bgl).


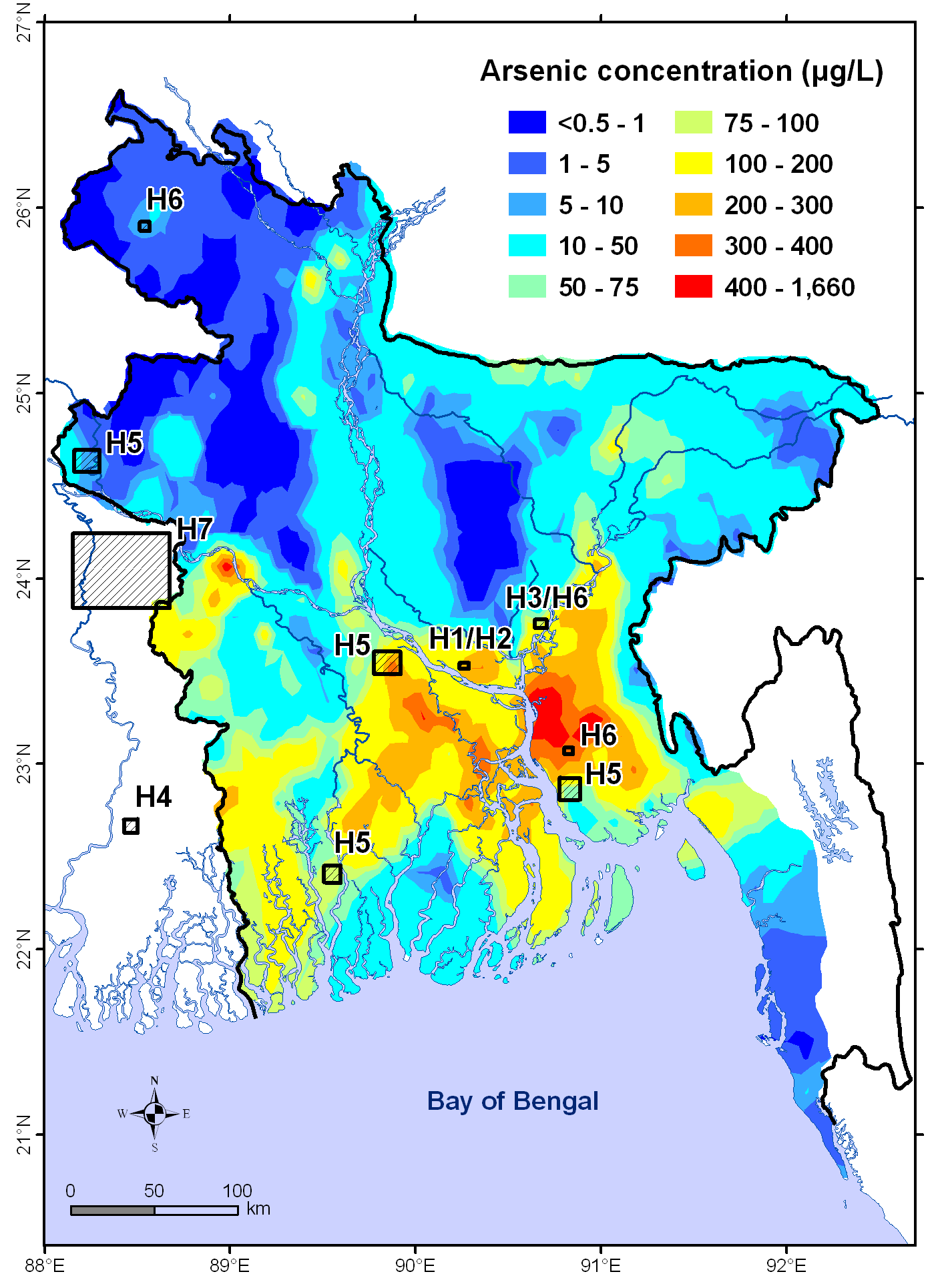


**Figure S2.** Spatial distribution of groundwater As concentrations in shallow (<50 m bgl) aquifers in Bangladesh. The gridded map of As concentrations was created by interpolating 2410 data points using the Inverse Distance Weighting method. Locations for the study sites associated with various As mobilization hypotheses are shown on the map. Keys: H-1: young carbon model [[*Harvey et al.*, 2002](#_ENREF_11); [*Harvey et al.*, 2006](#_ENREF_10)]; H-2: groundwater mixing model [[*Klump et al.*, 2006](#_ENREF_13)]; H-3 and H-6: aquifer flushing model [[*McArthur et al.*, 2004](#_ENREF_15); [*Stute et al.*, 2007](#_ENREF_22); [*van Geen et al.*, 2008](#_ENREF_23)]; H-4: As-peat hypothesis [[*Ravenscroft*, 2001](#_ENREF_17); [*McArthur et al.*, 2004](#_ENREF_15)]; and H-5: As-OC codeposition hypothesis [[*Meharg et al.*, 2006](#_ENREF_16)]; and H-4 and H-7: As-pond refuting hypothesis [[*Sengupta et al.*, 2008](#_ENREF_19); [*Datta et al.*, 2011](#_ENREF_7)].


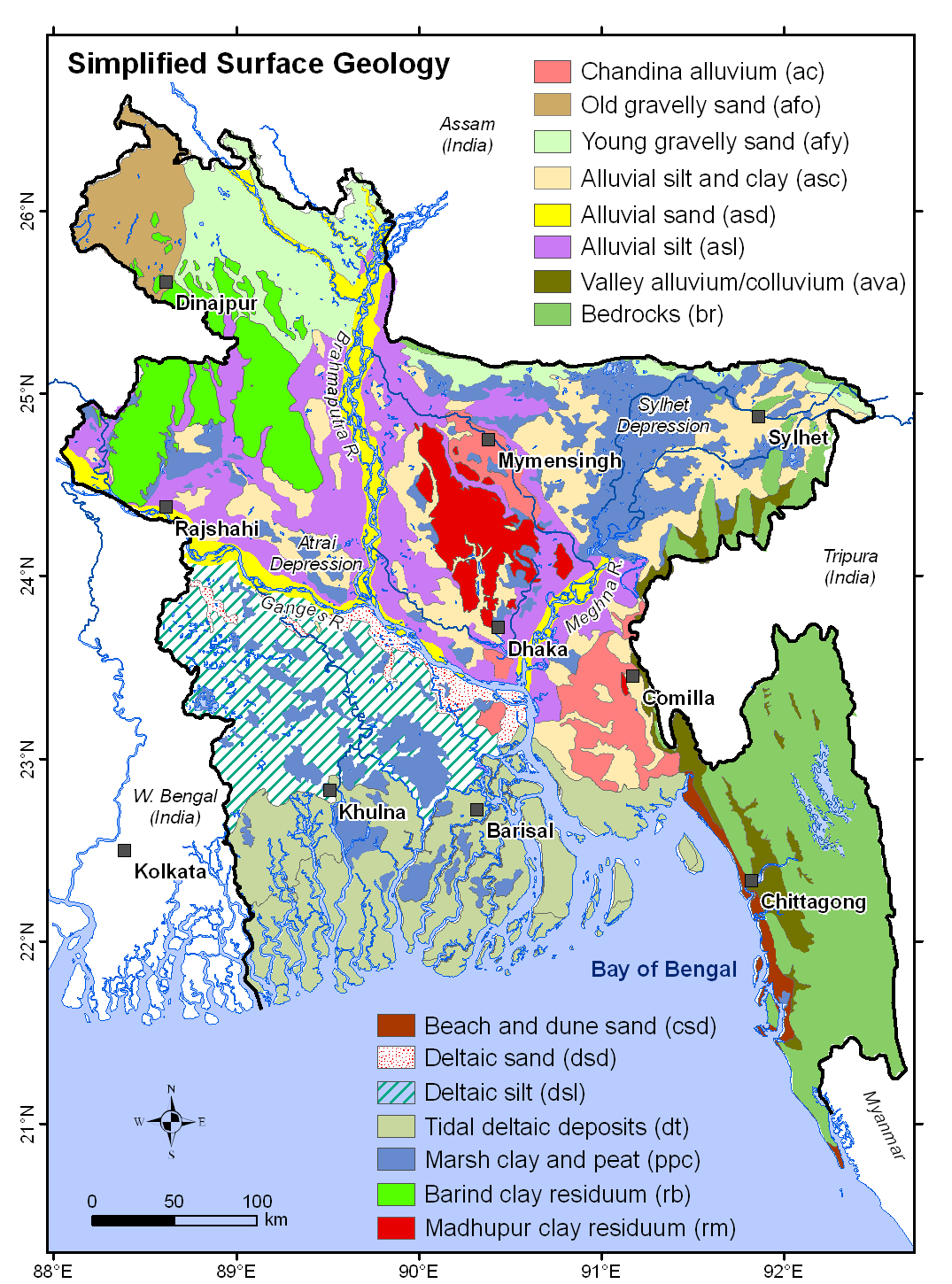


**Figure S3.** Simplified surface geological units in Bangladesh [[*Alam et al.*, 1990](#_ENREF_2)]. Major river channels and important district headquarters are also shown.


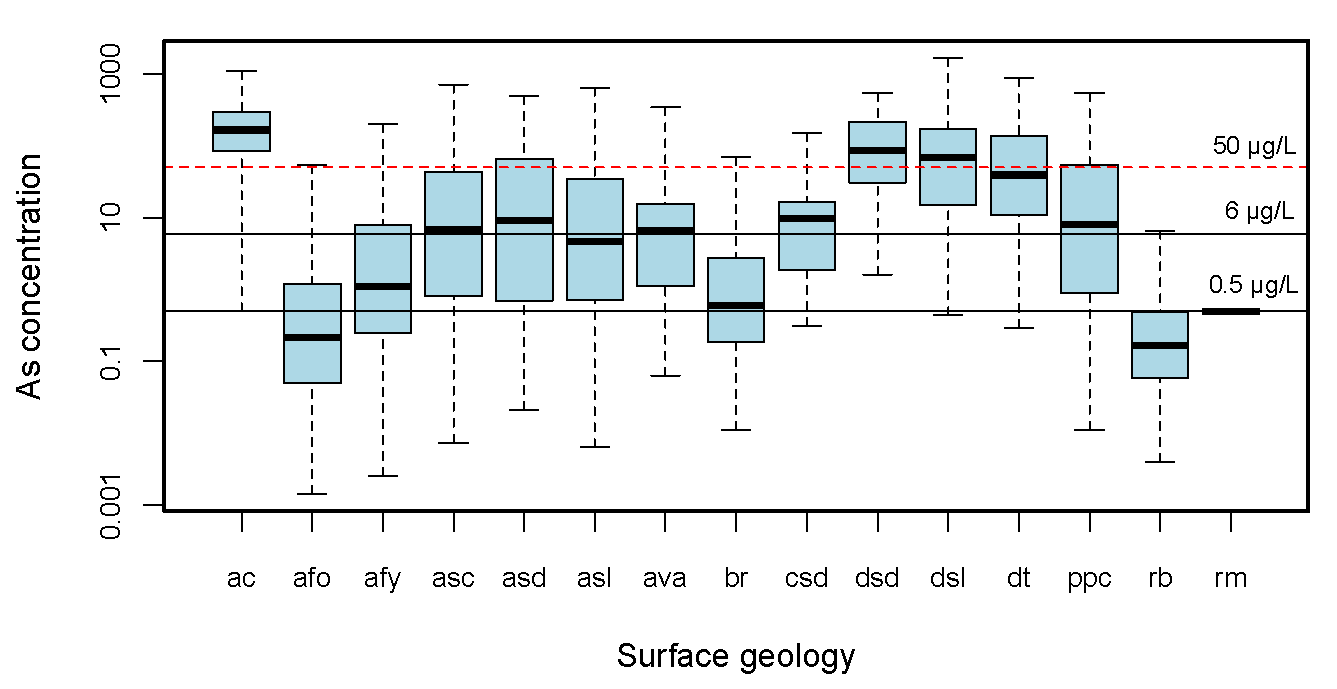


**Figure S4.** Box-and-Whisker plots showing As variations within various surface geological units in Bangladesh (see Figure S3 for name and location). The vertical axis is in log scale. The horizontal lines on the plot represent different threshold As concentrations; the black lines represent the minimum detection limits (6 and 0.5 µg L^-1^) of As measurements by two different methods (see [[*BGS and DPHE*, 2001](#_ENREF_4)] for details), and the broken red line represents the Bangladesh standard limit (50 µg L^-1^) of As in drinking water. Values below the detection limits are approximated using the regression on order statistics (ROS) technique designed for multiply censored analytical chemistry data [[*Helsel*, 2005](#_ENREF_12)]. The NADA package under the “R” statistical environment [[*Lee and Helsel*, 2007](#_ENREF_14)] was used for the analysis and plot.


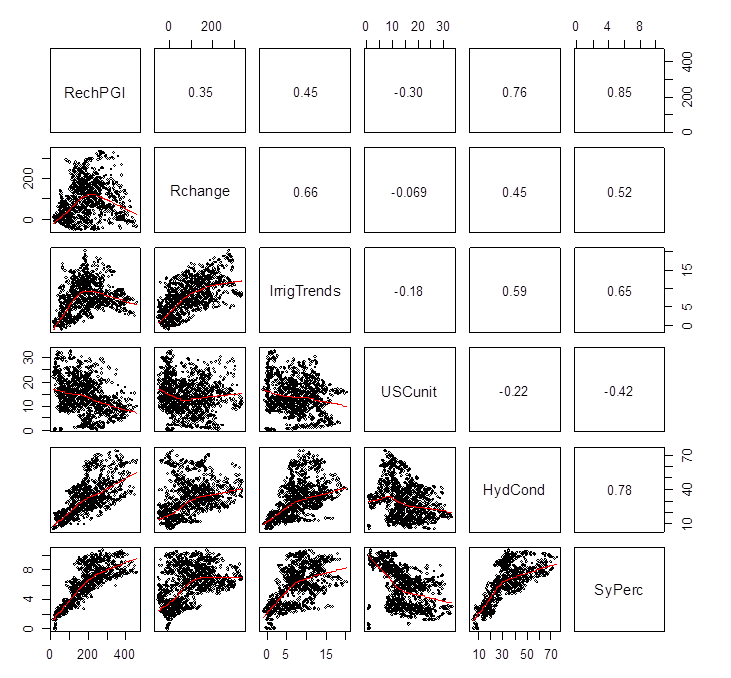


**Figure S5.** Pearson’s correlation matrices for some important covariates. Key: RechPGI=mean recharge (mm yr^-1^) for pre-developed groundwater-fed irrigation period, Rchange=net changes in groundwater recharge (mm), IrrigTrends=groundwater-fed irrigation trends (mm yr^-1^), USCunit=thickness (m) of surficial silt and clay (TSSC), HydCond=hydraulic conductivity (m d^-1^) and SyPerc=specific yield (%).

**
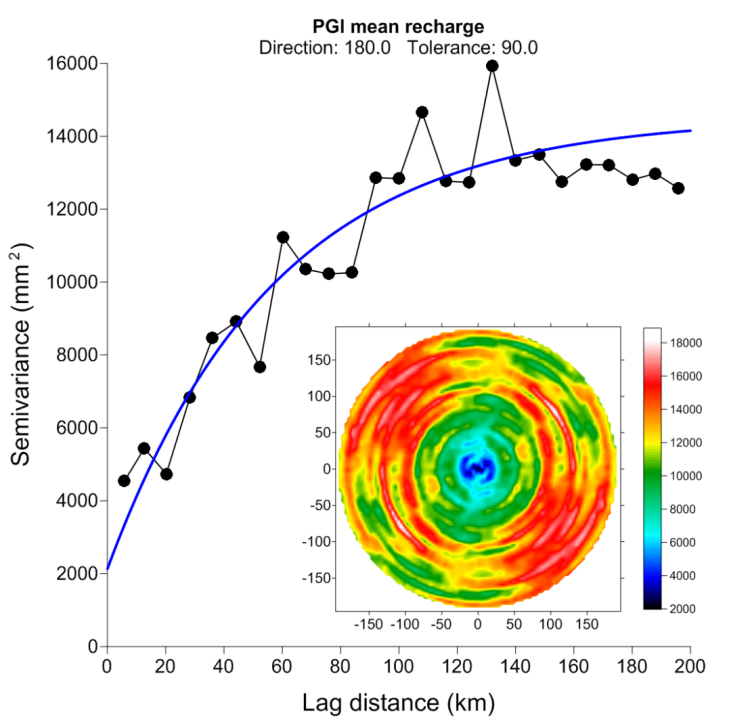
**

**Figure S6.** Variogram of original values (in mm) of the PGI mean groundwater recharge shows spatial dependence at the national scale. A strong spatial dependence exists in mean groundwater recharge values within a distance of around 100 km. The variogram surface map (inset image) shows strong anisotropy (i.e., directional dependence) in recharge locations: highest variations in recharge values are observed along NNW-SSE direction featuring a trend in mean recharge values at the national scale.


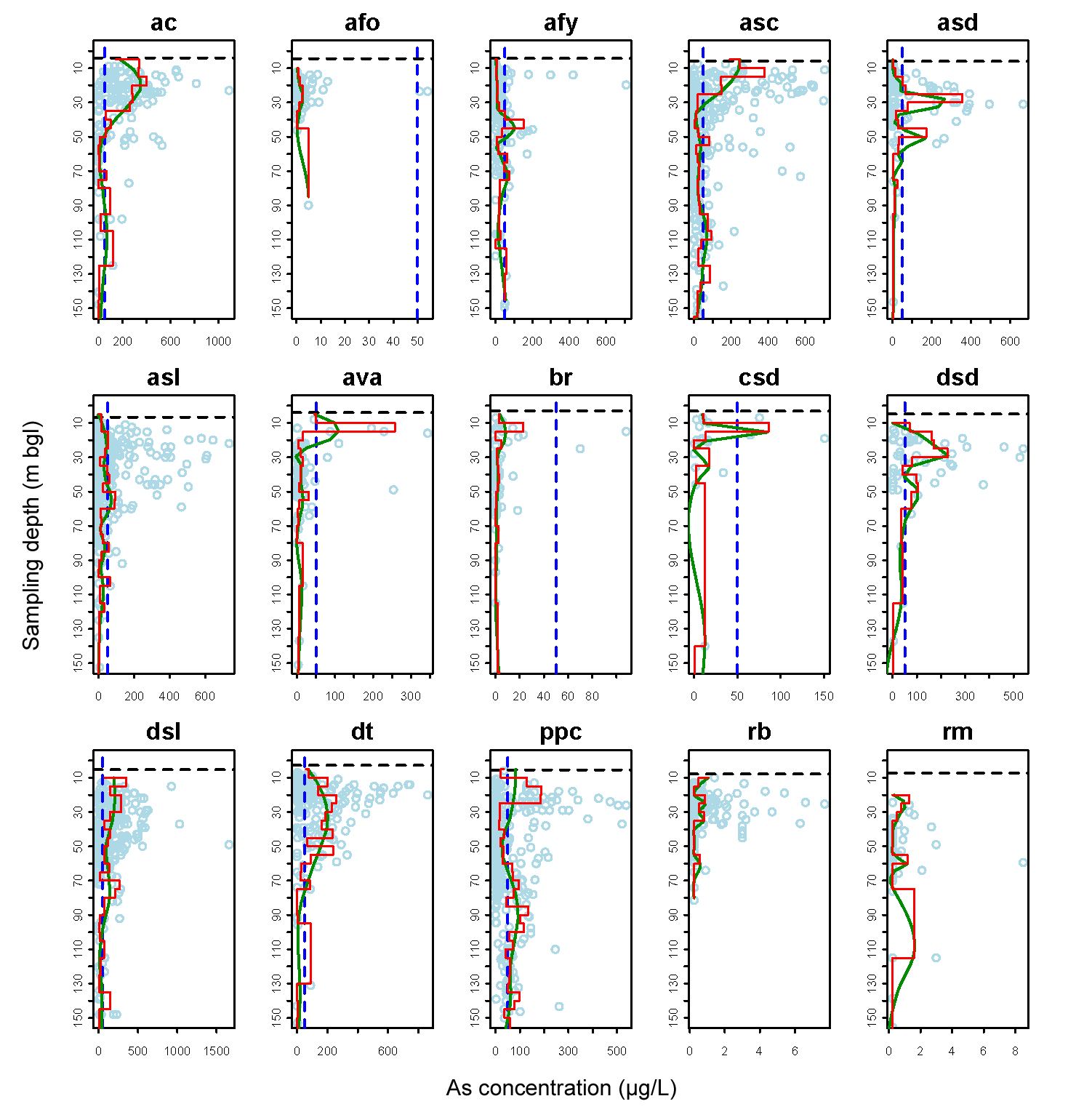


**Figure S7.** Groundwater As concentrations by surface geology. In each panel, blue circles are individual As data points (NHS As data); step-wise red lines are the 75^th^ percentile values in each 5-m bin of sampling depth; green lines are the Lowess smooth line; vertical, dashed blue lines represent Bangladesh As standard; and horizontal, dashed black lines are the mean dry-season groundwater table in each geological units (see Figure S3 for name and location).


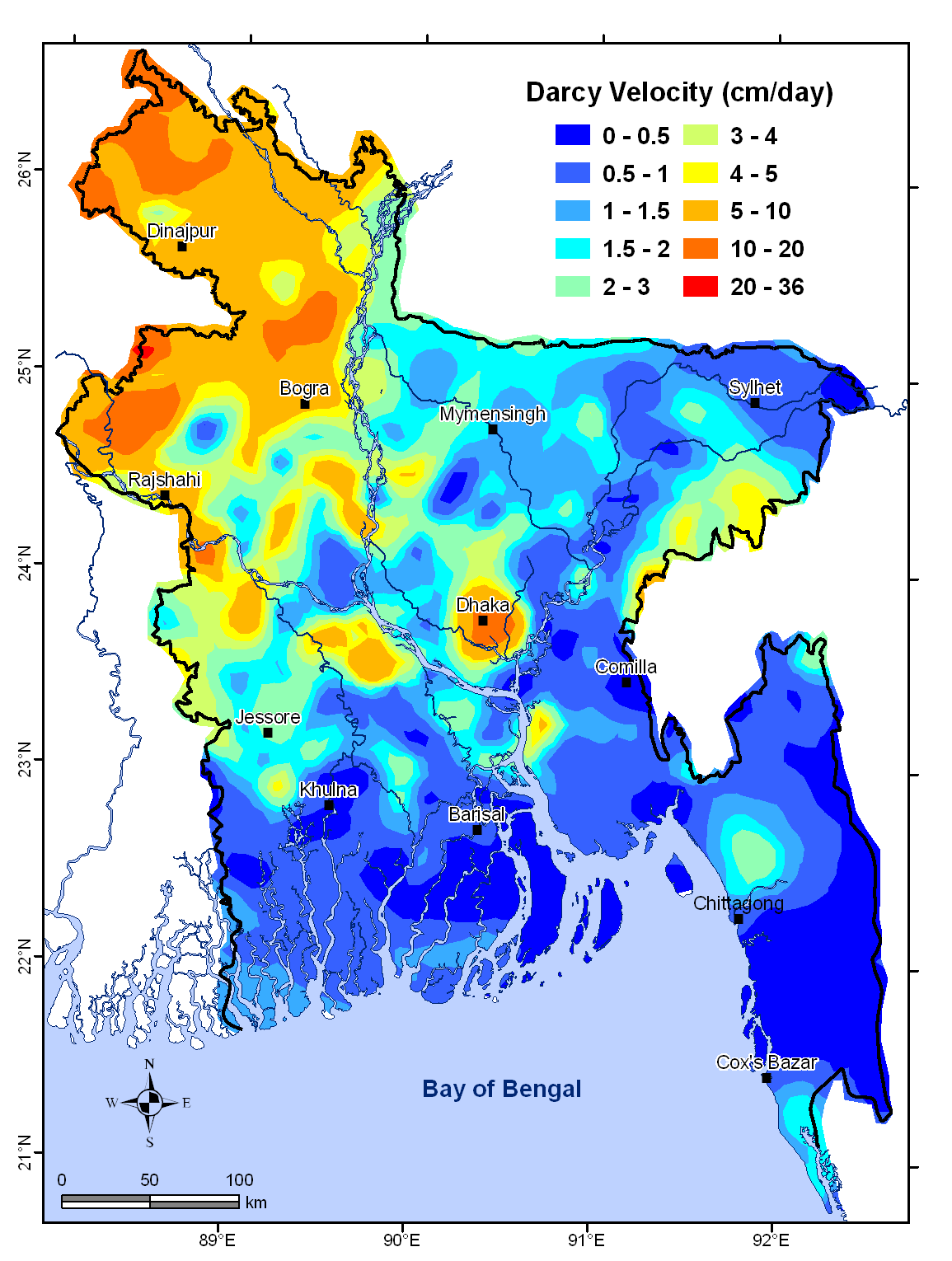


**Figure S8.** Groundwater flow velocity (Darcy flux) of shallow aquifers throughout Bangladesh. The Darcy flux map is created in the ArcGIS environment using spatial information on aquifer’s hydraulic conductivity and observed groundwater-level gradients compiled in this study.


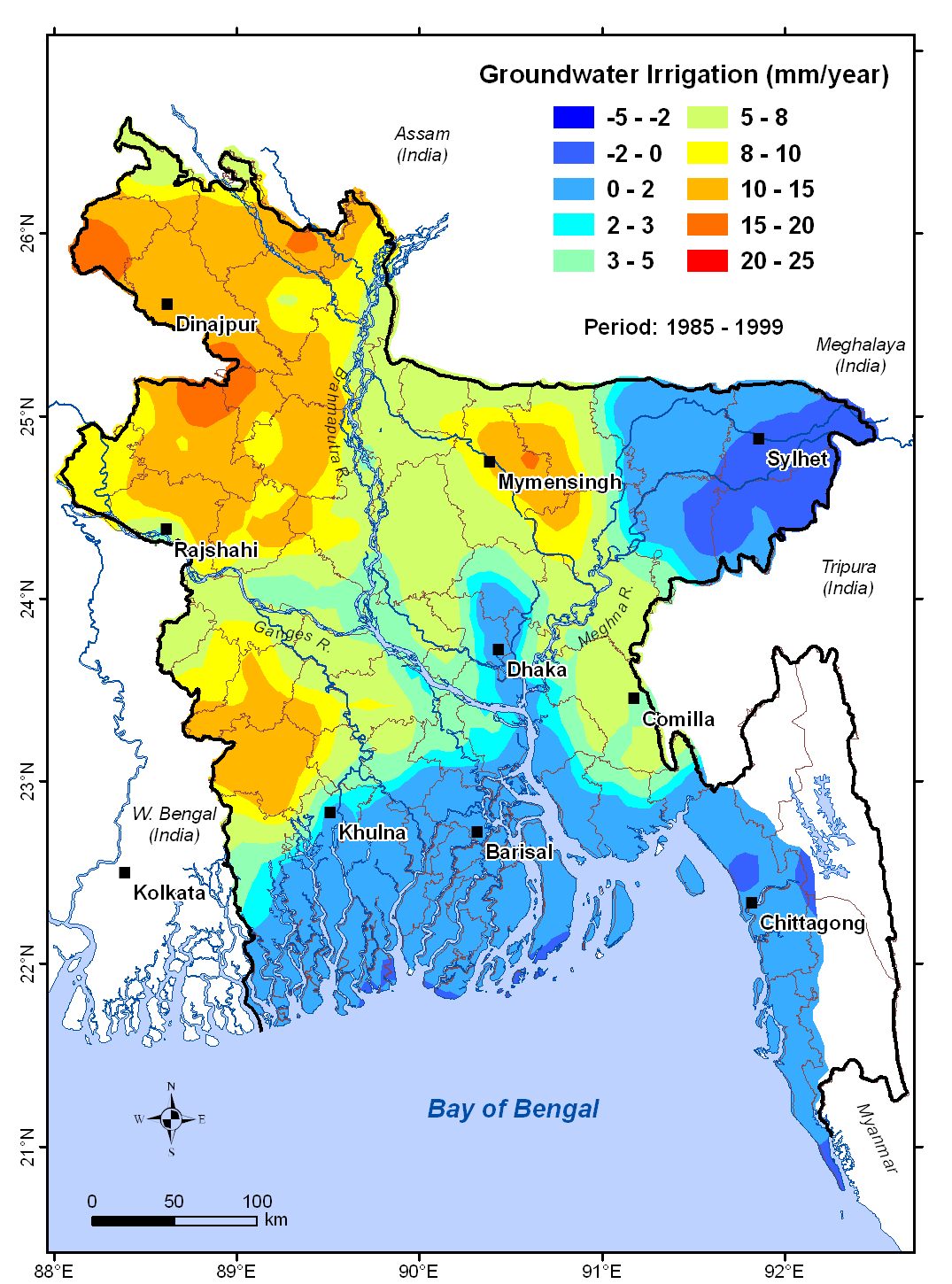


**Figure S9.** Temporal trends (mm yr^-1^) in groundwater-fed irrigation over the period of 1985 to 1999 in Bangladesh.


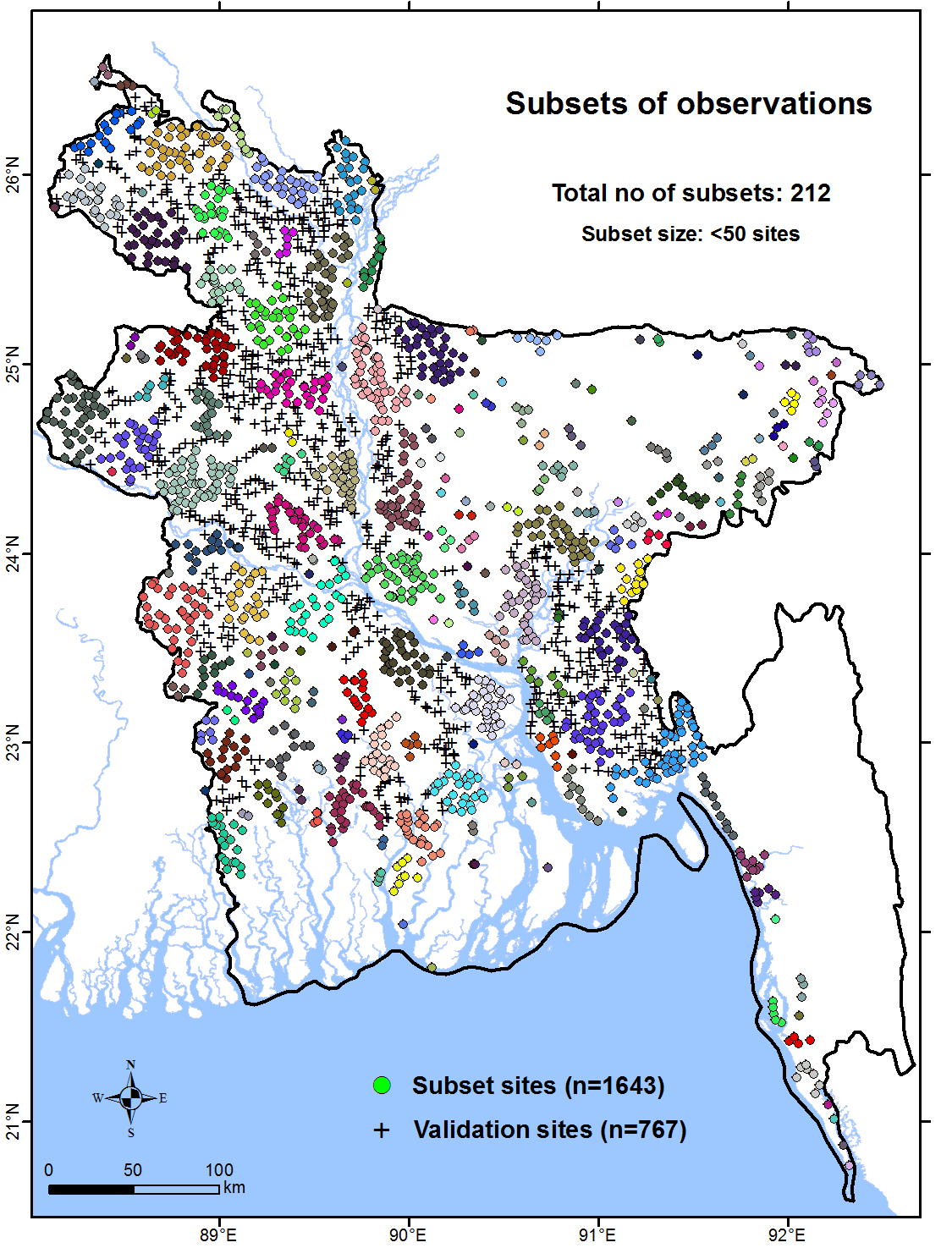


**Figure S10.** Spatial distribution of the color-coded subsets of As observations (*n*=1643) as well as the remaining observations (*n*=767) that were grouped using the hierarchical clustering method in order to resolve the inter-site spatial dependence in the As dataset. Subsets of observations were used to fit the calibration model and the remaining observations were used to validate the fitted model.


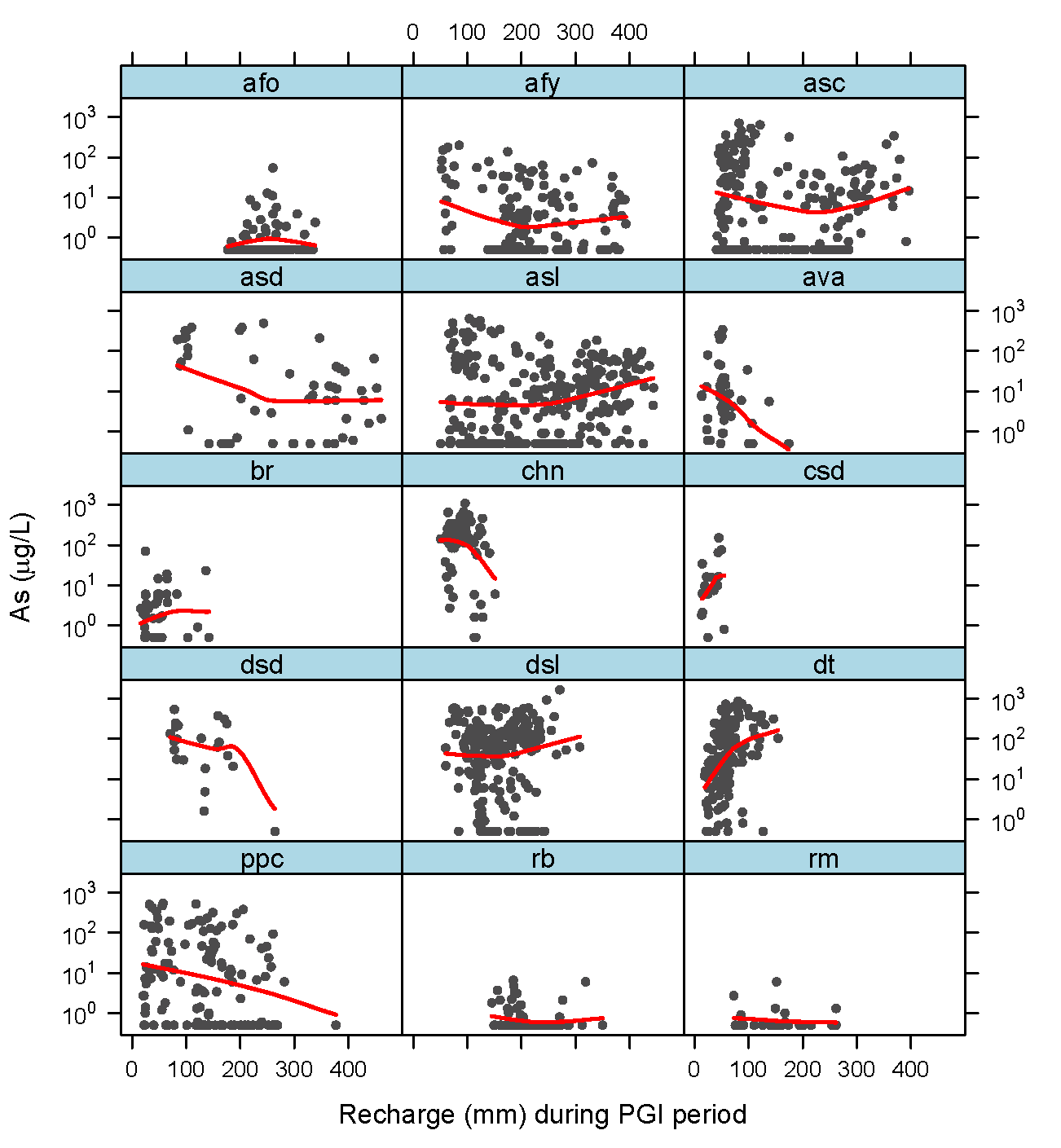


**Figure S11.** Variations in the relationship between As concentrations in groundwater and mean recharge (pre-developed groundwater-fed irrigation period, PGI, 1975−1980) to shallow aquifers within various geological units in Bangladesh. The red line in each individual panel is a nonparametric regression estimate (LOWESS) [[*Cleveland*, 1981](#_ENREF_6)] of the relationship between As concentrations and net recharge.


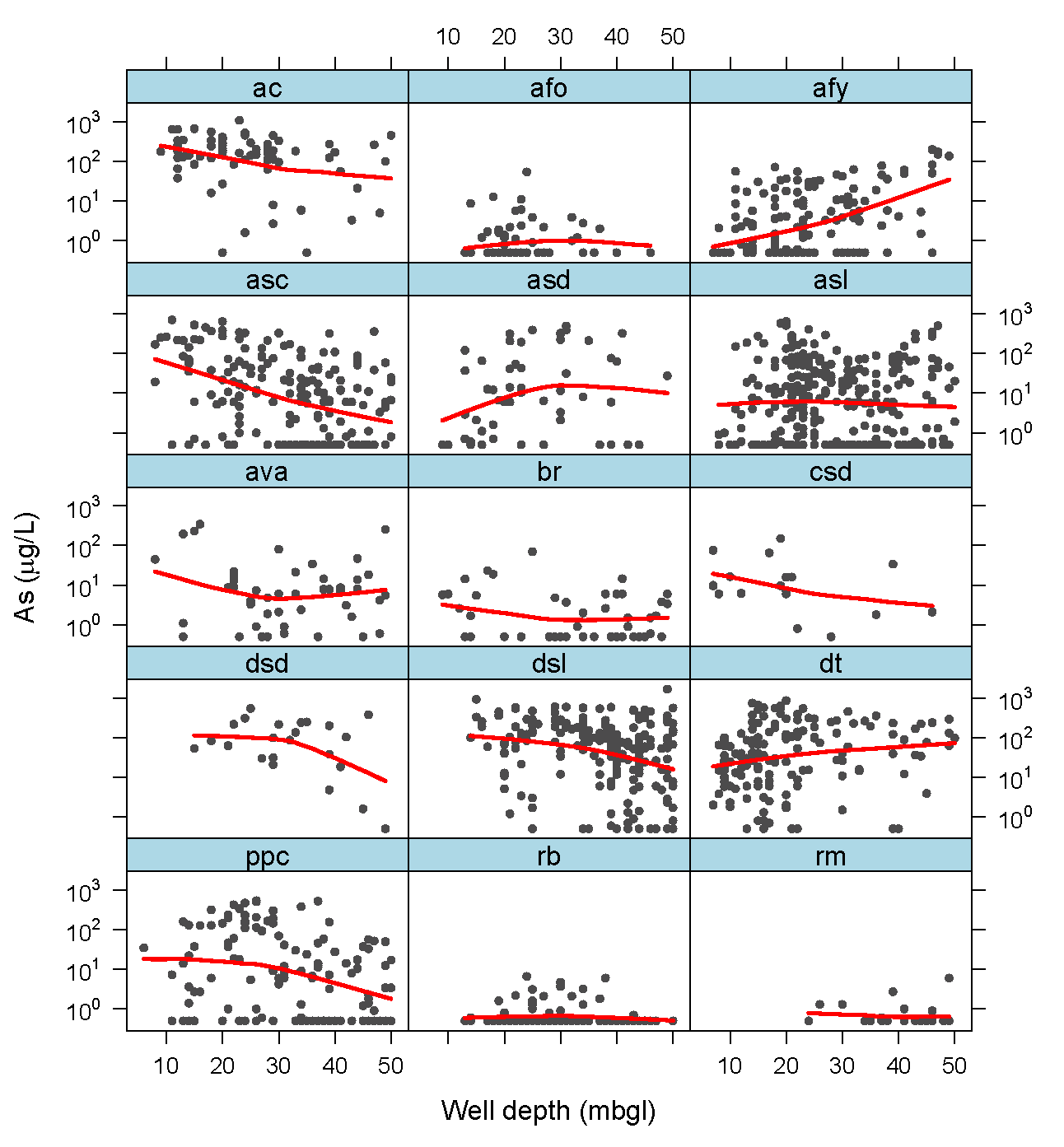


**Figure S12.** Variations in the relationships between As concentrations and sampling depths within different (*n*=15) surface geological units in Bangladesh. Depth to these surveyed wells are very shallow (<50 m bgl). The red line in each individual panel represents a locally-weighted polynomial regression (LOWESS) [[*Cleveland*, 1981](#_ENREF_6)] between As concentrations and well depth.


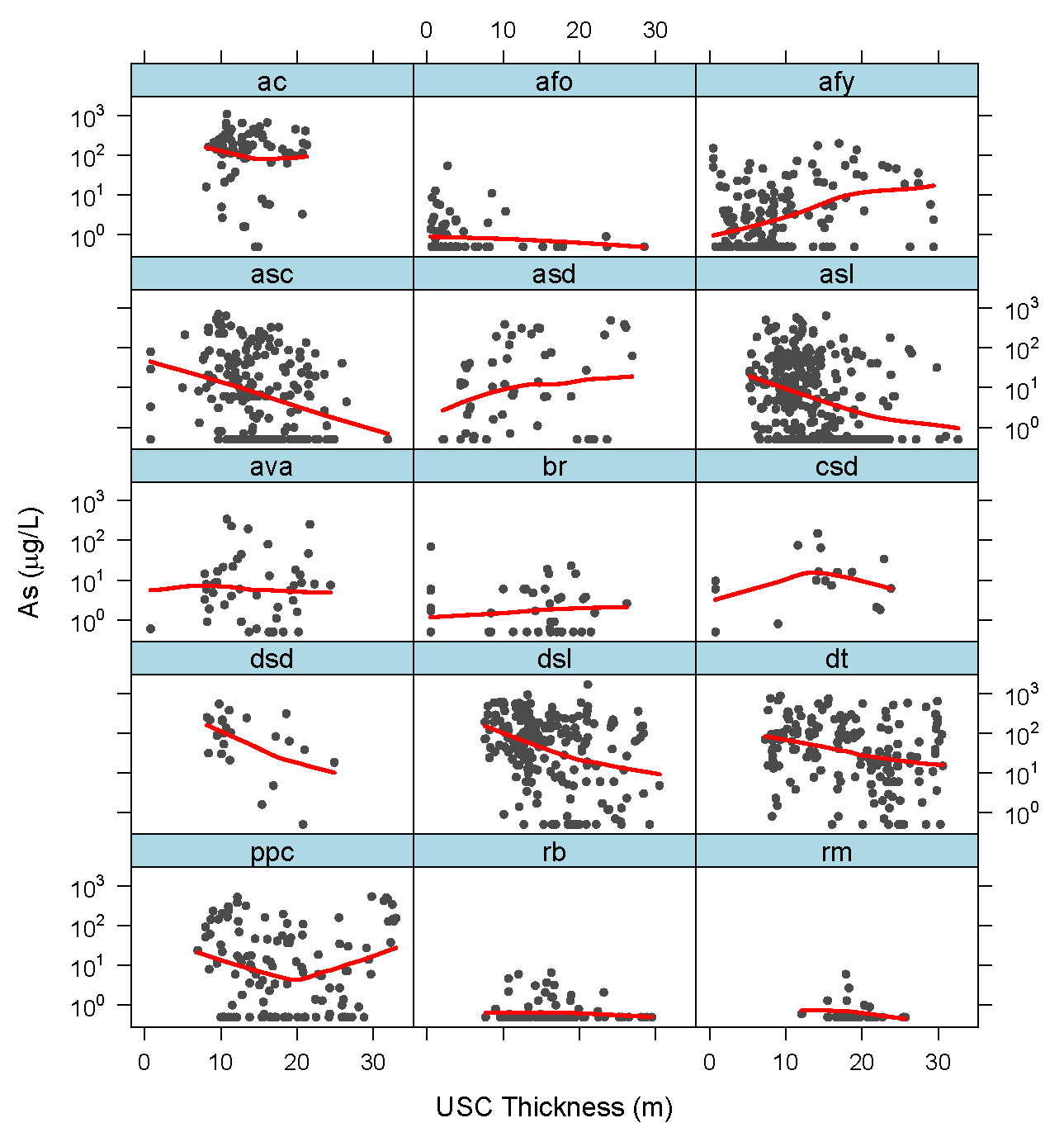


**TSSC (m)**

**Figure S13.** Variations in the relationships between As concentrations and the thickness of surficial silt and clay (TSSC) within different (*n*=15) surface geological units in Bangladesh. Depth to these surveyed wells are very shallow (<50 m bgl). The red line in each individual panel represents a locally-weighted polynomial regression (LOWESS) [[*Cleveland*, 1981](#_ENREF_6)] between As concentrations and TSSC.


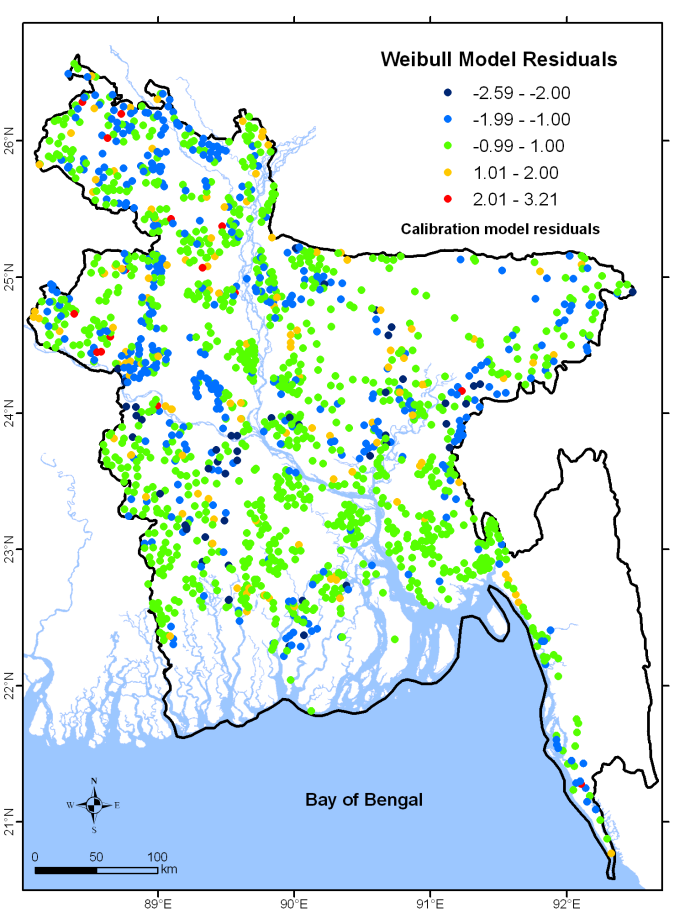

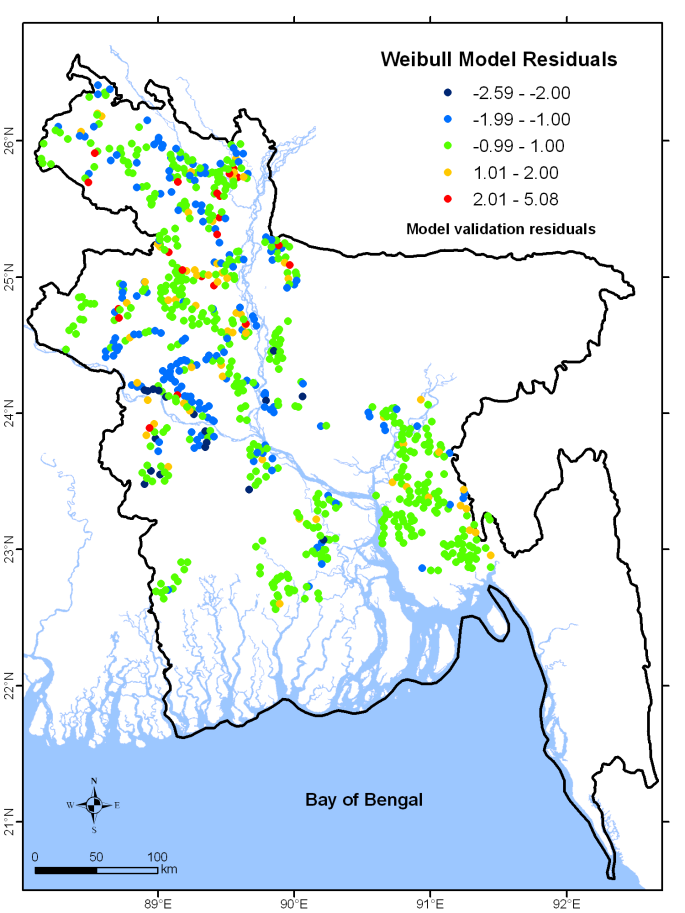


**(a)**

**(b)**

**Figure S14.** Spatial distribution of standardized deviance residuals from (a) the fitted, national-scale model, (b) validation of the fitted model using a subset of covariate datasets.


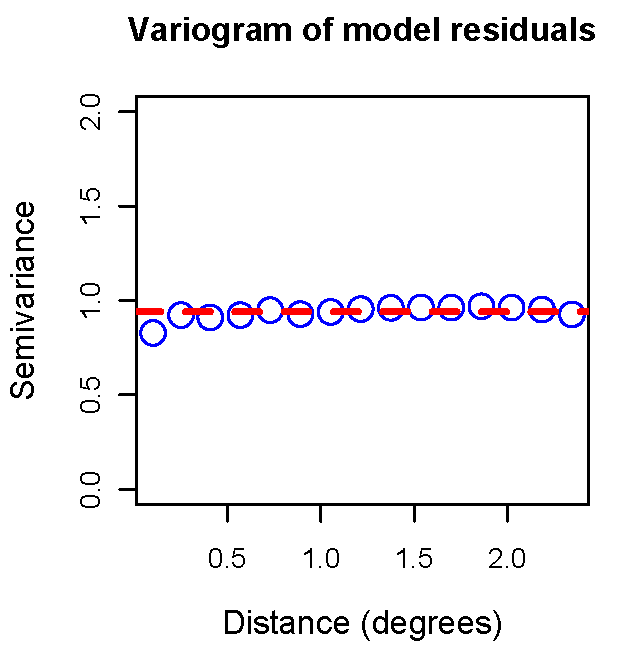


**Figure S15.** Variogram of the standardized deviance residuals for the fitted national-scale GRM; sample variance of the residuals is shown as dashed red line.


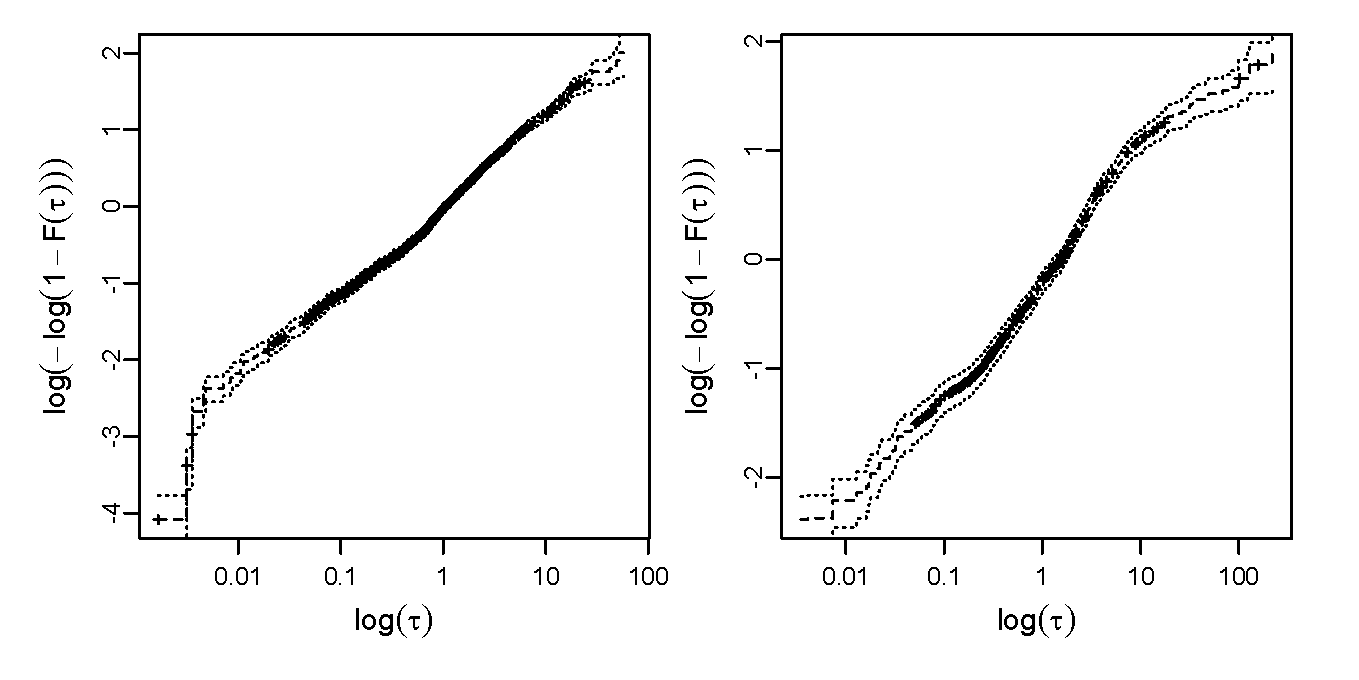


**(a)**

**(b)**

**Figure S16.** Weibull model assumption is checked with a plot of log(−log(1-F(τ_i_))) and log(τ). A straight line in the plot indicates that the assumption for the Weibull distribution is valid. Both plots (a) for the fitted, national-scale GRM, and (b) validation of the national-scale GRM suggest that the Weibull distribution is suitable for modeling groundwater As dataset.

### Supplementary Tables:

**Table S1.** Covariate datasets used in this study to explain As variations in groundwater, along with summary of conclusions of previous studies regarding their effects on As concentration. Units of measurement are given in Table S3.

| Group | Covariate datasets | Association with As | Reference |
| --- | --- | --- | --- |
| Geology and hydrogeological variables | Surface geological unit | Mobilization of groundwater As is largely geologically controlled | Refs [[*DPHE*, 1999](#_ENREF_9); [*BGS and DPHE*, 2001](#_ENREF_4); [*Ravenscroft*, 2001](#_ENREF_17); [*Ahmed et al.*, 2004](#_ENREF_1)] |
|  | Thickness of surficial silt and clay cover (TSSC) | Properties of near-surface deposits are related to As mobilization | Refs [[*DPHE*, 1999](#_ENREF_9); [*BGS and DPHE*, 2001](#_ENREF_4); [*Ravenscroft*, 2001](#_ENREF_17)] |
|  | Hydraulic conductivity of shallow aquifer | Hydraulic conductivity is associated with aquifer flushing and thus As in groundwater | Ref [[*Aziz et al.*, 2008](#_ENREF_3)] |
|  | Specific yield of shallow aquifer | Groundwater recharge is associated with specific yield and thereby control As mobilization | Refs [[*Harvey et al.*, 2006](#_ENREF_10); [*Klump et al.*, 2006](#_ENREF_13); [*Stute et al.*, 2007](#_ENREF_22)] |
|  | Darcy flux (shallow groundwater flow velocity) | Groundwater flow moves As from the site of release (distribution of As controlled by preferential flow paths ) | Refs [[*BGS and DPHE*, 2001](#_ENREF_4); [*Ravenscroft et al.*, 2005](#_ENREF_18)] |
|  | Depth to sampling well | Distribution of As is strongly related to depth (low As at greater depths) | Ref [[*BGS and DPHE*, 2001](#_ENREF_4)] |
| Hydrodynamic and groundwater recharge variables | Dry-season mean groundwater table (Note: not used in model fitting) | Low As concentrations in areas where dry-season groundwater table is deep | Ref [[*Ravenscroft et al.*, 2005](#_ENREF_18)] |
|  | Wet-season mean groundwater table | High As concentrations in areas where wet-season groundwater table is shallow | Ref [[*Shamsudduha et al.*, 2009](#_ENREF_21)] |
|  | Trend in mean annual groundwater levels | Low As in areas of declining groundwater levels | Ref [[*Shamsudduha et al.*, 2009](#_ENREF_21)] |
|  | Mean groundwater-level fluctuation | Low As concentrations in areas of limited groundwater fluctuations (annual range in groundwater levels) | Ref [[*DPHE*, 1999](#_ENREF_9)] |
|  | Net annual mean groundwater recharge (Pre groundwater (GW)-fed irrigation) | Role of recharge in As mobilization is controversial (recharge can either decrease or increase As in groundwater) | Refs [[*DPHE*, 1999](#_ENREF_9); [*Harvey et al.*, 2006](#_ENREF_10); [*Stute et al.*, 2007](#_ENREF_22); [*van Geen et al.*, 2008](#_ENREF_23)] |
|  | Net changes in mean recharge (Developed GW-fed irrigation − Pre GW-fed irrigation) | Role of long-term recharge trends in As mobilization is controversial (recharge can either decrease or increase As in groundwater) | Ref [[*Klump et al.*, 2006](#_ENREF_13)] |
| Geographical and seasonal factors | Geographic positions (sample latitudes and longitudes) | There are regional trends in groundwater As variations | Ref [[*Shamsudduha*, 2007](#_ENREF_20)] |
|  | Surface elevation | Low As concentrations in elevated areas; high As in low-lying areas | Ref [[*Shamsudduha et al.*, 2009](#_ENREF_21)] |
|  | Seasonality (sampling dates as a proxy) | No discernible seasonal pattern of As has been detected at the national scale | This study |
| Groundwater-fed irrigation | Slopes of linear trends (1985-1999) in groundwater-fed irrigation. See Section 1.2.6 for further details. | Role of groundwater-fed irrigation to As mobilization is controversial | Refs [[*Ravenscroft et al.*, 2005](#_ENREF_18); [*Harvey et al.*, 2006](#_ENREF_10); [*Klump et al.*, 2006](#_ENREF_13)] |

**Table S2.** Descriptive statistics of the NHS As data (*n*=2410) within different geological units in Bangladesh. Mean, median, and standard deviation of As observations are estimated using the ROS method [[*Helsel*, 2005](#_ENREF_12)] in the R statistical computing environment.

| **Geology** | **No of data** | **Censored** | **%Censored** | **Median** | **Mean** | **Std. deviation** | **Minimum** | **Maximum** |
| --- | --- | --- | --- | --- | --- | --- | --- | --- |
| ac | 121 | 2 | 1.6 | 182.0 | 221.6 | 185.4 | <0.5 | 1090.0 |
| afo | 109 | 68 | 62.4 | 0.3 | 2.2 | 7.3 | <0.5 | 54.2 |
| afy | 281 | 108 | 38.4 | 1.5 | 15.7 | 57.3 | <0.5 | 708.0 |
| asc | 285 | 92 | 32.3 | 9.1 | 77.5 | 147.4 | <0.5 | 704.0 |
| asd | 91 | 28 | 30.8 | 5.8 | 68.3 | 128.9 | <0.5 | 665.0 |
| asl | 496 | 141 | 28.2 | 5.1 | 45.1 | 102.7 | <0.5 | 735.0 |
| ava | 56 | 10 | 17.9 | 6.6 | 30.5 | 68.3 | <0.5 | 344.0 |
| br | 53 | 28 | 52.8 | 0.6 | 6.0 | 17.7 | <0.5 | 108.0 |
| csd | 18 | 3 | 16.7 | 9.7 | 23.6 | 38.4 | <0.5 | 151.0 |
| dsd | 43 | 4 | 9.3 | 72.0 | 123.6 | 145.2 | <0.5 | 540.0 |
| dsl | 280 | 34 | 12.1 | 67.9 | 134.2 | 187.0 | <0.5 | 1660.0 |
| dt | 192 | 15 | 8.0 | 48.0 | 118.9 | 163.3 | <0.5 | 862.0 |
| ppc | 159 | 50 | 31.4 | 9.1 | 65.5 | 111.9 | <0.5 | 538.0 |
| rb | 194 | 135 | 69.6 | 0.3 | 0.7 | 1.1 | <0.5 | 7.7 |
| rm | 31 | 25 | 80.6 | 0.4 | 0.4 | 0.1 | <0.5 | 6.0 |
| National | 2410 | 743 | 30.8 | 5.7 | 66.7 | 134.3 | <0.5 | 1660.0 |

**Table S3.** Descriptive statistics of covariates used to fit the generalized regression model for explaining the variation of As concentrations in groundwater in Bangladesh. Number of data points and standard deviation of original data points, and root mean square error (RMSE) for geostatistical interpolation of numerical covariates are provided.

| **Covariates / Factors** | **Data point (Std. dev.) / RMSE interpolation or Remarks** | **Data type and Unit** | **Mean**^†^ | **Median**^†^ | **Standard deviation**^†^ | **Data range**^†^ |
| --- | --- | --- | --- | --- | --- | --- |
| Surface geological unit | Vector data | Non-numeric or categorical; polygonal GIS layers | n.a. | n.a. | n.a. | 15 units |
| Thickness of surficial silt and clay cover (TSSC) | Data digitized from a map | Numerical (m); gridded dataset | 14.00 | 13.60 | 6.63 | 0.5 to 33.4 |
| Hydraulic conductivity | 280 (21.0) / 15.4 | Numerical (m d^-1^); gridded dataset | 30.85 | 29.30 | 15.53 | 5.7 to 75.8 |
| Specific yield | 305 (4.00) / 3.00 | Numerical; gridded dataset (%) | 5.82 | 6.00 | 2.51 | 0.1 to 10.7 |
| Darcy flux | Estimated from hydraulic conductivity and groundwater levels datasets | Numerical (cm d^-1^); gridded dataset | 3.63 | 2.33 | 3.75 | 0.05 to 31.5 |
| Well depth | n.a. (As dataset) | Measured/estimated depth (m bgl) to well screen | 27.88 | 26.00 | 10.78 | 6.0 to 50.0 |
| Wet-season groundwater table | 454 (1.99) / 1.34 | Numerical (m bgl); gridded dataset | 1.40 | 1.19 | 1.02 | 0.01 to 11.5 |
| Groundwater-level trends | 454 (16.7) / 12 | Numerical (cm yr^-1^); gridded dataset | −3.60 | −2.56 | 5.72 | −62.56 to 5.8 |
| Mean groundwater fluctuation | 454 (1.84) / 1.14 | Numerical (m); gridded dataset | 3.97 | 3.89 | 1.50 | 0.9 to 8.04 |
| Mean annual groundwater recharge (PGI) | 117 (101) / 62 | Numerical (mm yr^-1^); gridded dataset | 166.10 | 158.30 | 98.89 | 13.02 to 460.7 |
| Net changes in mean recharge (DGI−PGI) | 282 (164) / 78 | Numerical (mm); gridded dataset | 78.94 | 60.00 | 89.69 | −90 to 333 |
| Longitude | n.a. (As dataset) | Measured (GPS) coordinates (in degree) | 89.85 | 89.71 | 0.99 | 88.08 to 92.48 |
| Latitude | n.a. (As dataset) | Measured (GPS) coordinates (in degree) | 24.17 | 24.21 | 1.11 | 20.8 to 26.6 |
| Surface elevation | n.a. (DEM of 300-m spatial resolution) | GIS Raster, (m msl) | 14.85 | 10.93 | 13.85 | 0.63 to 93.8 |
| Seasonality (water sampling dates) | n.a. (As dataset) | Sampling dates (decimal year) | 1998.80 | 1998.42 | 0.56 | 1998.01 to 1999.93 |
| Slopes of linear trends in groundwater-fed irrigation | 645 (5) / 3.13 | Numerical (mm yr^-1^); gridded dataset | 6.88 | 6.80 | 4.63 | −1.1 to 20.2 |

Note: n.a. denotes ‘not appropriate’ for descriptive statistics or no spatial interpolation was performed.

^†^Descriptive statistics (mean, median, standard deviation and data range) are calculated from data points after extracting interpolated values of numerical covariates at As observations (*n*=2410).

**Table S4.** Summary of the national-scale GLM for the As dataset in Bangladesh providing estimated coefficients of model parameters and unadjusted and adjusted (within subsets of As data) standard errors with the corresponding the Wald test statistic (*z* value), and statistical significance (*P* value). DF denotes degree of freedom.

| **Covariates/Factors** | **Coefficient** | **DF** | **Std. error** | ***z* value^c^** | ***P* value^c^** |
| --- | --- | --- | --- | --- | --- |
| **Geology and hydrogeological variables:** |  |  |  |  |  |
| Surface geology^b^ | - | 14 | - | - | - |
| TSSC^a^ | -0.025 | 1 | 0.020 | -1.261 | 0.2075 |
| Hydraulic conductivity | -0.023 | 1 | 0.008 | -2.854 | 0.0043 |
| Specific yield | -0.068 | 1 | 0.107 | -0.640 | 0.5220 |
| Darcy flux | -0.014 | 1 | 0.022 | -0.608 | 0.5430 |
| Well depth^a^ | -0.013 | 1 | 0.010 | -1.327 | 0.1840 |
| **Hydrodynamic and groundwater recharge variables:** |  |  |  |  |  |
| Wet-season GWT | -0.004 | 1 | 0.094 | -0.046 | 0.9630 |
| Groundwater-level trends | 0.030 | 1 | 0.016 | 1.836 | 0.0664 |
| Mean groundwater fluctuation | -0.171 | 1 | 0.097 | -1.765 | 0.0776 |
| Mean PGI recharge^a^ | 0.001 | 1 | 0.004 | 0.167 | 0.8670 |
| Net changes in recharge | -0.004 | 1 | 0.001 | -3.251 | 0.0013 |
| **Geographical, altitudinal, and seasonal factors:** |  |  |  |  |  |
| Longitude (degree 1 Legendre) ^a^ | -0.817 | 1 | 0.411 | -1.989 | 0.0467 |
| Latitude (degree 1 Legendre) ^a^ | -0.978 | 1 | 0.612 | -1.599 | 0.1100 |
| Longitude (degree 2 Legendre) | -0.890 | 1 | 0.274 | -3.248 | 0.0012 |
| Latitude (degree 2 Legendre) | -1.099 | 1 | 0.622 | -1.766 | 0.0773 |
| Longitude1: Latitude1 | 0.269 | 1 | 0.717 | 0.367 | 0.7070 |
| Surface elevation | -0.002 | 1 | 0.016 | -0.152 | 0.8790 |
| Cosine (sampling date) | -0.679 | 1 | 6.470 | -0.105 | 0.9160 |
| Sine (sampling date) | 0.299 | 1 | 1.937 | 0.155 | 0.8770 |
| **Groundwater-fed irrigation:** |  |  |  |  |  |
| Irrigation trends | -0.050 | 1 | 0.024 | -2.056 | 0.0399 |
| **Statistical interaction terms:** |  |  |  |  |  |
| Geology : Well depth^b^ | - | 14 | - | - | - |
| Geology : Mean PGI recharge^b^ | - | 14 | - | - | - |
| Geology : TSSC^b^ | - | 14 | - | - | - |

Note: ^a^ Coefficients should be interpreted with their interactions and statistical significance is calculated using LR test; ^b^ model coefficients, standard errors, and *P* values for categorical surface geology covariate and its interactions with well depth, mean PGI recharge and TSSC are not summarized here but can be produced using the model codes and datasets; ^c^ z and *P* values were adjusted within the subsets in the input datasets (output summarized from GRM using the psm() function).

**Table S5.** Summary of the regional-scale GLM for the As dataset in Bangladesh providing estimated coefficients of model parameters and unadjusted and adjusted (within subsets of As data) standard errors with the corresponding the Wald test statistic (*z* value), and statistical significance (*P* value). DF denotes degree of freedom.

| **Covariates/Factors** | **Coefficient** | **DF** | **Std. error** | ***z* value^c^** | ***P* value^c^** |
| --- | --- | --- | --- | --- | --- |
| **Geology and hydrogeological variables:** |  |  |  |  |  |
| Surface geology^b^ | - | 14 | - | - | - |
| TSSC^a^ | -0.025 | 1 | 0.020 | -1.340 | 0.1787 |
| Hydraulic conductivity | -0.024 | 1 | 0.008 | -2.810 | 0.0049 |
| Specific yield | -0.051 | 1 | 0.113 | -0.450 | 0.6523 |
| Darcy flux | -0.019 | 1 | 0.023 | -0.810 | 0.4172 |
| Well depth^a^ | -0.002 | 1 | 0.010 | -0.190 | 0.8495 |
| **Hydrodynamic and groundwater recharge variables:** |  |  |  |  |  |
| Wet-season GWT | -0.151 | 1 | 0.113 | -1.330 | 0.1823 |
| Groundwater-level trends | 0.025 | 1 | 0.019 | 1.320 | 0.1859 |
| Mean groundwater fluctuation | -0.230 | 1 | 0.101 | -2.270 | 0.0231 |
| Mean PGI recharge^a^ | 0.0003 | 1 | 0.005 | -0.060 | 0.9514 |
| Net changes in recharge | -0.004 | 1 | 0.001 | -3.720 | 0.0002 |
| **Geographical, altitudinal, and seasonal factors:** |  |  |  |  |  |
| Longitude (degree 1 Legendre) ^a^ | -1.100 | 1 | 0.425 | -2.590 | 0.0097 |
| Latitude (degree 1 Legendre) ^a^ | -0.530 | 1 | 0.668 | -0.790 | 0.4272 |
| Longitude (degree 2 Legendre) | -0.949 | 1 | 0.276 | -3.440 | 0.0006 |
| Latitude (degree 2 Legendre) | -1.949 | 1 | 0.676 | -2.880 | 0.0039 |
| Longitude1: Latitude1 | 0.380 | 1 | 0.772 | 0.490 | 0.6226 |
| Surface elevation | 0.007 | 1 | 0.018 | 0.400 | 0.6920 |
| Cosine (sampling date) | 1.580 | 1 | 6.596 | 0.240 | 0.8107 |
| Sine (sampling date) | 0.761 | 1 | 1.967 | 0.390 | 0.6989 |
| **Groundwater-fed irrigation:** |  |  |  |  |  |
| Irrigation trends | -0.055 | 1 | 0.025 | -2.20 | 0.0279 |
| **Statistical interaction terms:** |  |  |  |  |  |
| Geology : Well depth^b^ | - | 14 | - | - | - |
| Geology : Mean PGI recharge^b^ | - | 14 | - | - | - |
| Geology : TSSC^b^ | - | 14 | - | - | - |

Note: ^a^ Coefficients should be interpreted with their interactions and statistical significance is calculated using LR test; ^b^ model coefficients, standard errors, and *P* values for categorical surface geology covariate and its interactions with well depth, mean PGI recharge and TSSC are not summarized here but can be produced using the model codes and datasets; ^c^ z and *P* values were adjusted within the subsets in the input datasets (output summarized from GRM using the psm() function).

### Appendix A: Adjusted standard errors and likelihood ratio (LR) tests

This Appendix provides a brief summary of the procedures that are used to adjust standard errors and likelihood ratios for unmodeled inter-site dependence, when models are fitted using maximum likelihood under the assumption that the observations are independent. Throughout, the “prime” symbol $'$ denotes the transpose of a vector or matrix.

Consider a model involving a vector $\boldsymbol{\theta}=\left( \theta_{1},\cdots,\theta_{p} \right)'$of unknown parameters (corresponding to the regression coefficients in our GRM), which are to be estimated using data $\boldsymbol{y}=\left( y_{1},\cdots,y_{n} \right)^{'}$ (corresponding to the As observations). Maximum likelihood estimates can be obtained by maximizing the logarithm of a likelihood function. If the observations are assumed independent then the likelihood can be written as a product as in equation (4), and its logarithm is a sum of contributions from each individual observation $l\left( \boldsymbol{\theta};\boldsymbol{y} \right)=\sum_{i=1}^{n} l_{i}\left( \boldsymbol{\theta};y_{i} \right)$ say (the precise form of these terms is unimportant for the present discussion). Denote by $\hat{\boldsymbol{\theta}}$ the value of $\boldsymbol{\theta}$ for which $l\left( \boldsymbol{\theta};\boldsymbol{y} \right)$ is maximized, and by $\boldsymbol{\theta}_{\boldsymbol{0}}$ the ‘true’ value of $\boldsymbol{\theta}$ i.e. the value corresponding to the mechanism that generated the data. Moreover, let $\boldsymbol{U}\left( \boldsymbol{\theta} \right)= \partial l\left( \boldsymbol{\theta};\boldsymbol{y} \right)/\partial\boldsymbol{\theta}$ be the gradient vector of the log-likelihood, and let $\boldsymbol{H}= -E\left[ \partial^{2}l\left( \boldsymbol{\theta};\boldsymbol{y} \right)/\partial\boldsymbol{\theta}\partial\boldsymbol{\theta}^{'}|_{\boldsymbol{\theta}=\boldsymbol{\theta}_{0}} \right]$ be the matrix of expected second derivatives of $-l\left( \boldsymbol{\theta};\boldsymbol{y} \right)$ evaluated at $\boldsymbol{\theta}_{\boldsymbol{0}}$.Then, under general conditions (see [[*Davison*, 2003](#_ENREF_8)], p147) and if the sample size *n* is large enough, $\hat{\boldsymbol{\theta}}$ has approximately a multivariate normal distribution with expected value $\boldsymbol{\theta}_{\boldsymbol{0}}$ and covariance matrix $\boldsymbol{H}^{-1}\boldsymbol{V}\boldsymbol{H}^{-1}$ where $\boldsymbol{V}$ is the covariance matrix of$\boldsymbol{U}\left( \boldsymbol{\theta}_{0} \right)$. The standard errors of the individual parameter estimates are the square roots of the corresponding diagonal elements of this covariance matrix. Moreover, if the observations really are independent, then $\boldsymbol{H}=\boldsymbol{V}$ and the covariance matrix reduces to $\boldsymbol{H}^{-1}$. This can be easily estimated from the matrix of second derivatives of $l\left( \boldsymbol{\theta};\boldsymbol{y} \right)$ evaluated at $\hat{\boldsymbol{\theta}}$ (this matrix is often produced in software as a by-product of gradient-based numerical optimization procedures, so no extra work is required to obtain it).

If the observations are not independent, the “independence” log-likelihood $l\left( \boldsymbol{\theta};\boldsymbol{y} \right)$ can still be used to estimate $\boldsymbol{\theta}$. The theory outlined above remains valid, but the covariance matrix of $\hat{\boldsymbol{\theta}}$ does not reduce to $\boldsymbol{H}^{-1}$: it is thus necessary to estimate $\boldsymbol{V}$ as well as $\boldsymbol{H}$. This is usually done by partitioning the observations into subsets, in such a way that dependence occurs between observations within the same subset but different subsets are independent. Noting that $\boldsymbol{U}\left( \boldsymbol{\theta} \right)$, like $l\left( \boldsymbol{\theta};\boldsymbol{y} \right)$, is a sum of contributions from each observation, and denoting by $M$ the total number of subsets, we can write $\boldsymbol{U}\left( \boldsymbol{\theta} \right)=\sum_{m=1}^{M} \boldsymbol{U}_{m}\left( \boldsymbol{\theta} \right)$ say, where $\boldsymbol{U}_{m}\left( \boldsymbol{\theta} \right)$ is the contribution from the *m*^th^ subset. Now, because the subsets are independent, we have $Var\left[ \boldsymbol{U}\left( \boldsymbol{\theta} \right) \right]=\sum_{m=1}^{M} Var\left[ \boldsymbol{U}_{m}\left( \boldsymbol{\theta} \right) \right]$ so that $\boldsymbol{V}=\sum_{m=1}^{M} Var\left[ \boldsymbol{U}_{m}\left( \boldsymbol{\theta}_{0} \right) \right]$. Moreover, it can be shown that the expected value of $\boldsymbol{U}_{m}\left( \boldsymbol{\theta}_{0} \right)$ is zero for all *m*, so that $Var\left[ \boldsymbol{U}_{m}\left( \boldsymbol{\theta}_{0} \right) \right]=E\left[ \boldsymbol{U}_{m}\left( \boldsymbol{\theta}_{0} \right)\boldsymbol{U}_{\boldsymbol{m}}^{\boldsymbol{'}}\left( \boldsymbol{\theta}_{0} \right) \right]$. We thus have $\boldsymbol{V}=\sum_{m=1}^{M} E\left[ \boldsymbol{U}_{m}\left( \boldsymbol{\theta}_{0} \right)\boldsymbol{U}_{\boldsymbol{m}}^{\boldsymbol{'}}\left( \boldsymbol{\theta}_{0} \right) \right]=E\left[ \sum_{m=1}^{M} \boldsymbol{U}_{m}\left( \boldsymbol{\theta}_{0} \right)\boldsymbol{U}_{\boldsymbol{m}}^{\boldsymbol{'}}\left( \boldsymbol{\theta}_{0} \right) \right]$. If *M* is large, the variance of a sum of *M* terms is small compared with its expectation, whence $\boldsymbol{V}$ can be estimated as $\hat{\boldsymbol{V}}=\sum_{m=1}^{M} \boldsymbol{U}_{m}\left( \hat{\boldsymbol{\theta}} \right)\boldsymbol{U}_{\boldsymbol{m}}^{\boldsymbol{'}}\left( \hat{\boldsymbol{\theta}} \right)$ (this argument can be made rigorous). $\hat{\boldsymbol{V}}$ can be calculated straightforwardly providing the $\boldsymbol{\theta}$-derivatives of the log-likelihood contributions can be evaluated, and then combined with the estimate of $\boldsymbol{H}$ to obtain an “adjusted” covariance matrix and standard errors. Our code, provided in the online supplement, demonstrates how this is done for the GRM.

The theory above also underpins the dependence-adjusted likelihood ratio test proposed by [[*Chandler and Bate*, 2007](#_ENREF_5)]. Suppose we wish to test the hypothesis that $k$ components of $\boldsymbol{\theta}$ are zero in our GRM (i.e. that the associated covariates have no influence on As concentrations). Conventionally, this is done by dropping the corresponding terms from the model and refitting. The log-likelihood obtained from this “reduced model” $l\left( \tilde{\boldsymbol{\theta}};\boldsymbol{y} \right)$ say, will be less than $l\left( \hat{\boldsymbol{\theta}};\boldsymbol{y} \right)$. Under the null hypothesis that the data were generated from the reduced model, and if the observations are independent, the quantity $2\left[ l\left( \hat{\boldsymbol{\theta}};\boldsymbol{y} \right)-l\left( \tilde{\boldsymbol{\theta}};\boldsymbol{y} \right) \right]$ has approximately a chi-squared distribution with *k* degrees of freedom. The theory underlying this result relies on the fact that the curvature of the log-likelihood function is determined by the matrix $\boldsymbol{H}$**,** and that $\boldsymbol{H}=\boldsymbol{V}$ when the observations are independent. The dependence-adjusted test uses exactly the same procedure, but replaces the log-likelihood function $l\left( \boldsymbol{\theta};\boldsymbol{y} \right)$ with an adjusted function, $l_{ADJ}\left( \boldsymbol{\theta};\boldsymbol{y} \right)$ say, satisfying $l_{ADJ}\left( \hat{\boldsymbol{\theta}};\boldsymbol{y} \right)=l\left( \hat{\boldsymbol{\theta}};\boldsymbol{y} \right)$ and with second derivative matrix $-\boldsymbol{H}\boldsymbol{V}^{-1}\boldsymbol{H}$ in place of $-\boldsymbol{H}$. Specifically, the adjusted log-likelihood used in the present work is the “vertically scaled” version defined at equation (25) of [[*Chandler and Bate*, 2007](#_ENREF_5)]:

$$l_{ADJ}\left( \boldsymbol{\theta};\boldsymbol{y} \right)= l\left( \hat{\boldsymbol{\theta}};\boldsymbol{y} \right)+\left( \boldsymbol{\theta}-\hat{\boldsymbol{\theta}} \right)^{'}\boldsymbol{H}\boldsymbol{V}^{-1}\boldsymbol{H}\left( \boldsymbol{\theta}-\hat{\boldsymbol{\theta}} \right)\frac{l\left( \boldsymbol{\theta};\boldsymbol{y} \right)-l\left( \hat{\boldsymbol{\theta}};\boldsymbol{y} \right)}{\left( \boldsymbol{\theta}-\hat{\boldsymbol{\theta}} \right)'\boldsymbol{H}\left( \boldsymbol{\theta}-\hat{\boldsymbol{\theta}} \right)}.$$

Again, our code in the online supplement demonstrates how this is implemented in practice.

**References**

Ahmed, K. M., P. Bhattacharya, M. A. Hasan, S. H. Akhter, S. M. M. Alam, M. A. H. Bhuyian, M. B. Imam, A. A. Khan, and O. Sracek (2004), Arsenic enrichment in groundwater of the alluvial aquifers in Bangladesh: an overview, *Appl. Geochem.*, *19*(2), 181-200.

Alam, M. K., A. K. M. S. Hasan, M. R. Khan, and J. W. Whitney (1990), Geological map of Bangladesh, Geological Survey of Bangladesh, Dhaka.

Aziz, Z., et al. (2008), Impact of local recharge on arsenic concentrations in shallow aquifers inferred from the electromagnetic conductivity of soils in Araihazar, Bangladesh, *Wat. Resour. Res.*, *44*, W07416.

BGS, and DPHE (2001), Arsenic contamination of groundwater in Bangladesh, *WC/00/19*, 267 pp, British Geological Survey, Keyworth.

Chandler, R. E., and S. Bate (2007), Inference for clustered data using the independence log-likelihood, *Biometrika*, *94*, 167-183.

Cleveland, W. S. (1981), LOWESS: A program for smoothing scatterplots by robust locally weighted regression, *The American Statistician*, *35*, 54.

Datta, S., A. W. Neal, T. J. Mohajerin, T. Ocheltree, B. E. Rosenheim, C. D. White, and K. H. Johannesson (2011), Perennial ponds are not an important source of water or dissolved organic matter to groundwaters with high arsenic concentrations in West Bengal, India, *Geophys. Res. Lett.*, *38*, L20404.

Davison, A. C. (2003), *Statistical Models, Cambridge Series in Statistical and Probabilistic Mathematics*, Cambridge University Press, Cambridge.

DPHE (1999), Groundwater studies for Arsenic contamination in Bangladesh, Rapid Investigation Phase, Final Report, British Geological Survey (BGS) and Mott MacDonald Ltd (UK).

Harvey, C. F., et al. (2006), Groundwater dynamics and arsenic contamination in Bangladesh, *Chem. Geol.*, *228*, 112-136.

Harvey, C. F., et al. (2002), Arsenic mobility and groundwater extraction in Bangladesh, *Science*, *298*, 1602-1606.

Helsel, D. R. (2005), *Nondetects and Data Analysis: Statistics for Censored Environmental Data*, John Wiley and Sons, New York.

Klump, S., R. Kipfer, O. A. Cirpka, C. F. Harvey, M. S. Brennwald, K. N. Ashfaque, A. B. M. Badruzzaman, S. J. Hug, and D. M. Imboden (2006), Groundwater Dynamics and Arsenic Mobilization in Bangladesh Assessed Using Noble Gases and Tritium, *Environ. Sci. Technol.*, *40*(1), 243-250.

Lee, L., and D. R. Helsel (2007), Statistical analysis of water-quality data containing multiple detection limits II: S-language software for nonparametric distribution modeling and hypothesis testing, *Computers & Geosciences 33*, 696-704.

McArthur, J. M., et al. (2004), Natural organic matter in sedimentary basins and its relation to arsenic in anoxic groundwater: the example of West Bengal and its worldwide implications, *Appl. Geochem.*, *19*(8), 1255-1293.

Meharg, A. A., C. Scrimgeour, S. A. Hossain, K. Fuller, K. Cruickshank, P. N. Williams, and D. G. Kinniburgh (2006), Codeposition of Organic Carbon and Arsenic in Bengal Delta Aquifers, *Environ. Sci. Technol.*, *40*(16), 4928-4935.

Ravenscroft, P. (2001), Distribution of groundwater arsenic in the Bangladesh related to geology, in *Groundwater arsenic contamination in the Bengal Delta Plain of Bangladesh*, edited by P. Bhattacharya, G. Jacks and A. A. Khan, pp. 4-56, Proc KTH-Dhaka University Seminar, KTH Special Publication.

Ravenscroft, P., W. G. Burgess, K. M. Ahmed, M. Burren, and J. Perrin (2005), Arsenic in groundwater of the Bengal Basin, Bangladesh: Distribution, field relations, and hydrogeological setting, *Hydrogeol. J.*, *13*, 727–751.

Sengupta, S., J. M. McArthur, A. K. Sarkar, M. Leng, P. Ravenscroft, R. J. Howarth, and D. Banerjee (2008), Do ponds cause arsenic-pollution of groundwater in the Bengal Basin?: an answer from West Bengal, *Environ. Sci. Technol.*, *42*(14), 5156-5164.

Shamsudduha, M. (2007), Spatial Variability and Prediction Modeling of Groundwater Arsenic Distributions in the Shallowest Alluvial Aquifers in Bangladesh, *J. Spat. Hydro.*, *7*(2), 33-46.

Shamsudduha, M., L. J. Marzen, A. Uddin, M.-K. Lee, and J. A. Saunders (2009), Spatial relationship of groundwater arsenic distribution with regional topography and water-table fluctuations in the shallow aquifers in Bangladesh, *Environ. Geol.*, *57*, 1521-1535.

Stute, M., Y. Zheng, P. Schlosser, A. Horneman, R. K. Dhar, M. A. Hoque, A. A. Seddique, M. Shamsudduha, K. M. Ahmed, and A. van Geen (2007), Hydrological control of As concentrations in Bangladesh groundwater, *Wat. Resour. Res.*, *43*, W09417.

van Geen, A., et al. (2008), Flushing history as a hydrogeological control on the regional distribution of arsenic in shallow groundwater of the Bengal basin, *Environ. Sci. Technol.*, *42*(7), 2283–2288.
